# *ACTB*, *CDKN1B*, *GAPDH*, *GRB2*, *RHOA* and *SDCBP* Were Identified as Reference Genes in Neuroendocrine Lung Cancer via the nCounter Technology

**DOI:** 10.1371/journal.pone.0165181

**Published:** 2016-11-01

**Authors:** Robert Fred Henry Walter, Robert Werner, Claudia Vollbrecht, Thomas Hager, Elena Flom, Daniel Christian Christoph, Jan Schmeller, Kurt Werner Schmid, Jeremias Wohlschlaeger, Fabian Dominik Mairinger

**Affiliations:** 1 Ruhrlandklinik, West German Lung Center, University Hospital Essen, University of Duisburg-Essen, Essen, Germany; 2 Institute of Pathology, University Hospital Essen, University of Duisburg-Essen, Essen, Germany; 3 Institute of Pathology, Helios Klinikum Emil von Behring, Berlin, Germany; 4 German Cancer Consortium (DKTK), partnersite Berlin, Germany; 5 Charité - Universitaetsmedizin Berlin, Institute of Pathology, Berlin, Germany; 6 German Cancer Research Center (DKFZ), Heidelberg, Germany; 7 Department of Medical Oncology, West German Cancer Center, University Hospital Essen, University of Duisburg-Essen, Essen, Germany; 8 Institute of Pathology, Ev.-Luth. Diakonissenkrankenhaus Flensburg, Flensburg, Germany; Universita degli Studi di Napoli Federico II, ITALY

## Abstract

**Background:**

Neuroendocrine lung cancer (NELC) represents 25% of all lung cancer cases and large patient collectives exist as formalin-fixed, paraffin-embedded (FFPE) tissue only. FFPE is controversially discussed as source for molecular biological analyses and reference genes for NELC are poorly establishes.

**Material and methods:**

Forty-three representative FFPE-specimens were used for mRNA expression analysis using the digital nCounter technology (NanoString). Based on recent literature, a total of 91 mRNA targets were investigated as potential tumor markers or reference genes. The geNorm, NormFinder algorithms and coefficient of correlation were used to identify the most stable reference genes. Statistical analysis was performed by using the R programming environment (version 3.1.1)

**Results:**

RNA integrity (RIN) ranged from 1.8 to 2.6 and concentrations from 34 to 2,109 ng/μl. However, the nCounter technology gave evaluable results for all samples tested. *ACTB*, *CDKN1B*, *GAPDH*, *GRB2*, *RHOA* and *SDCBP* were identified as constantly expressed genes with high stability (M-)values according to geNorm, NormFinder and coefficients of correlation.

**Conclusion:**

FFPE-derived mRNA is suitable for molecular biological investigations via the nCounter technology, although it is highly degraded. *ACTB*, *CDKN1B*, *GAPDH*, *GRB2*, *RHOA* and *SDCBP* are potent reference genes in neuroendocrine tumors of the lung.

## Introduction

Neuroendocrine lung cancer (NELC) represents 25% of all lung cancer cases and is divided into four subgroups (typical (TC) and atypical carcinoids (AC), large- (LCNEC) and small-cell (neuroendocrine) cancer (SCLC)) [1].

The majority of these clinical samples are stored as formalin-fixed, paraffin-embedded (FFPE) tissue. FFPE samples can be stored at ambient temperature with minimal logistical effort and costs. The main disadvantage of formalin fixation is the degradation and alteration of nucleic acids by oxidative deamination and cross-linking [2–4]. Besides, temperature shifts, ultraviolet light and reactive oxygen species have a negative impact on nucleic acid stability and integrity [4] leading to highly degraded nucleic acids. Storage of fresh-frozen tissue may help to overcome the mentioned problems, but the costs are high and biobanking is in the early stages of development. An ideal method for the investigation of FFPE is the nCounter technology from NanoString. The technology enables the detection of short target regions (~100 nt) making it an ideal method for analyzing fragmented nucleic acids from FFPE tissue. Fragments of 100 nt were reported to be detectable with nearly 100% efficiency [3, 5–7]. Reis *et al*. compared FFPE versus fresh-frozen samples using the nCounter technique and reported a high comparability of the results [3]. Very short regions of approximately 100 nt are targeted by two specific oligo probes that hybridize to the region of interest [6, 8] without any enzymatic step, hence overcoming limitation of other methods (e.g. PCR), which suffer from enzymatic alterations such as amplification efficiencies [9, 10]. Fluorescent barcodes allow for a simulations detection of up to 800 different targets per sample [6, 8]. To compensate for technical or biological variations, normalization of the raw data should be performed. So far, reference gene normalization seemed to be the best choice for quantitative mRNA analysis, although alternative quantification/normalization methods are under discussion: These alternatives include standardization to cell number and RNA mass quantification (most frequently used in Northern Blot analysis), but these methods are hardly applicable for clinical samples with low RNA quality and unknown cell content [9]. Hence, identification of reference genes is need.

But detectable and reliable expression levels as well as robustness of potential reference genes are discussed controversially, because expression is disease- and tissue-type dependent [9, 11]. Using inappropriate internal references can have a drastic impact as it can lead to errors in data acquisition and analysis [10].

Based on recent literature, a custom CodeSet for expression profiling in NELC was designed and tested on the nCounter platform. To identify potent reference genes in neuroendocrine lung cancer, two common algorithms (geNorm and NormFinder) were applied. Here we present reliable reference genes in NELC that enable a proper normalization, which builds the fundament for good quality data and provides the basis for research approaches such as biomarker detection.

## Material and Methods

Representative samples of each tumor subtype (15 TC, 9 AC, 7 LCNEC and 12 SCLC) were used for the mRNA expression analysis using the nCounter platform (NanoString Technologies, Seattle, WA, USA). Specimens from 2005 till 2012 were taken from the tumor bank at the Institute of Pathology, University Hospital Essen, University Duisburg-Essen (Germany). Tumor classification was performed according to the *WHO Classification Of Tumours* guidelines (2004) [12] and TNM-staging was based on the *UICC Classification of Malignant Tumours* [13]. The study was conducted retrospectively for the identification of potential tumor markers and reference genes. The study was approved by the ethical committee of the University Hospital Essen (ID: 13-5382-BO) and conforms to the principles outlined in the declaration of Helsinki.

### RNA Isolation and RNA Integrity Assessment

Three to five paraffin sections with a thickness of 4 μm per sample were deparaffinized with xylene prior to RNA extraction using the RNeasy FFPE kit (Qiagen, Hilden, Germany) according to the manufacturer’s recommendations with slight adjustments as described earlier [14, 15]. Total RNA concentrations were measured using a Nanodrop 1000 instrument (Thermo Fisher Scientific, Waltham, USA) and an Agilent 2100 Bioanalyzer (eukaryote total RNA Nano, version 2.6, Agilent Technologies, Santa Clara, USA). RNA integrity was assessed using the Agilent 2100 bioanalyzer.

### nCounter CodeSet Design and Expression Analysis

Multiple genes involved in tumor-associated pathways and neuroendocrine differentiation were included in a custom CodeSet using the standard chemistry. The CodeSet was designed to contain a total of 91 genes with different signature genes for each subgroup as described elsewhere [7, 14–16]. The CodeSet was designed and synthesized at NanoString Technologies, Seattle, USA (S1 Table for properties of the CodeSet). Total RNA (100 ng) including miRNA was analyzed at the NanoString nCounter Core Facility at the University of Heidelberg, Germany.

### nCounter Data Processing and Statistical Analysis

For each gene, raw NanoString counts were subjected to a technical background correction. Therefore, the average of the negative controls counts plus two-times the standard deviation was calculated and subtracted from each target count. Afterwards, either the geNorm or NormFinder algorithm was used to calculate the gene expression stability measure (M-value) for all tested genes. In brief, the M-value of a certain gene, calculated by the geNorm algorithm, is the arrhythmic mean of all pairwise variations of potential control genes [9]. The NormFinder algorithm estimates a) the intra- and inter-sample variation leading to a distribution, which is then b) transformed to a one-dimensional value by defining a value of the mean plus one standard deviation [17]. Genes with average and median counts below 500 were not considered as potential reference genes, because mean and median counts <500 indicate that a fraction of the samples present with absent expression for a certain gene. All statistical and graphical analyses were performed using the R statistical programming environment (v 3.1.1). For dichotomous factors such as gender and expression level the Wilcoxon Mann-Whitney rank sum test was applied. The Kruskal-Wallis test was used to correlate tumor type and gene expression. Correlations between gene expression and TNM-stages were analyzed by Spearman’s rank correlation test. Scatterplots were generated and coefficients of determination (R^2^) were calculated for pairwise comparison of potential reference genes. As control, two previously identified tumor markers (*CDK6* and *TYMS* [14, 15]) were correlated with the reference genes as described above.

The level of statistical significance was defined as p≤0.05.

## Results

Forty-three tumor samples were investigated (16 TC (27%) and 13 AC (22%), 16 LCNEC (27%) and 15 SCLC (25%)). Twenty-five female (58%) and 16 male patients (37%) were analyzed. For two patients the gender remained inconclusive after anonymization.

**Fig 1:**
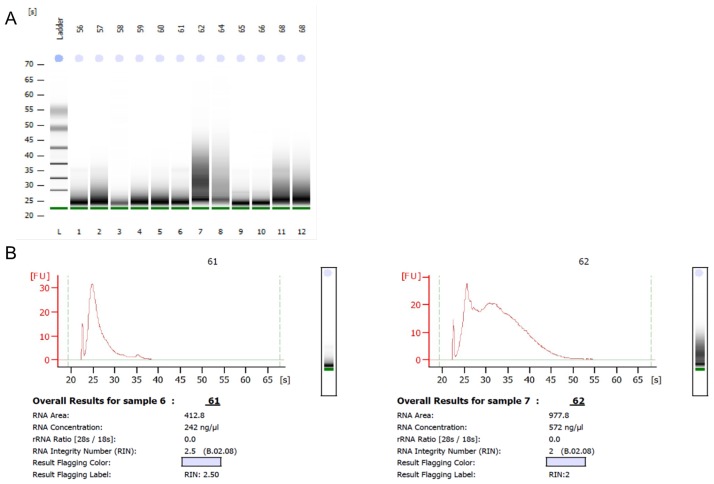
A and B show gel-smear analysis to assess the RNA quantity and quality (RIN) in total RNA derived from formalin-fixed, paraffin-embedded tissue. **Fig 1A** depicts a representative smear gel analysis of twelve samples. A ladder was included to allow size calculation. The microfluid analysis shows that RNA from FFPE is highly degraded giving no distinct size patterns.**Fig 1B** depicts the electropherogram of two representative samples. The rRNA Ratio (28s/18s) is used to calculate the RNA quality according to an algorithm supplied by the manufacturer. Neither 28s nor 18s bands can be found for FFPE-derived RNA leading to considerably low RNA integrity numbers (RIN). RNA concentration is calculated from the area under the curve.

RNA integrity (RIN) ranged from 1.8 to 2.6 and concentrations from 34 to 2,109 ng/μl. Although RIN was considerably low, all tested specimens gave evaluable results with respect to the nCounter expression analysis. Representative results are summarized in S2 Table. Besides, Fig 1A and 1B presents exemplary data of the smear analysis.

The NormFinder algorithm identified the following genes as most stably expressed: *GRB2* (M-value = 0.422), *CDKN1B* (M-value = 0.4787), *GAPDH* (M-value = 0.5228), *SDCBP* (M-value = 0.6232), *PNN* (M-value = 0.6563) and *ACTB* (M-value = 0.6646).

Whereas, the geNorm algorithm identified these stable reference genes: *CDKN1B* (M-value = 4.45), *GRB2* (M-value = 4.45), *RHOA* (M-value = 4.48), *SDCBP* (M-value = 4.45), *GAPDH* (M-value = 4.58) and *LDHB* (M-value = 4.59).

With respect to the 20 most stable reference genes identified by both algorithms, a consensus for 17 genes (85%) was found. The results for mean and median expression as well as M-values for both algorithms are summarized in S3 Table.

Statistical analysis for TNM-criteria revealed significant correlations of tumor type and gene expression of *ACTB* (p = 0.016), *CAT* (p = 0.041), *LDHB* (p = 0.024) and *SDCBP* (p = 0.044), which would render insignificant after a multivariate analysis correction (e.g. Bonferroni correction or false discovery rate adjustment (FDR)) (S1 Fig). Similarly, *LDHB* (p = 0.023) and *PNN* (p = 0.0.44) were significantly associated with grade of the tumor (data not shown). For age of the FFPE sample and gender no significant correlations were noted.

**Fig 2:**
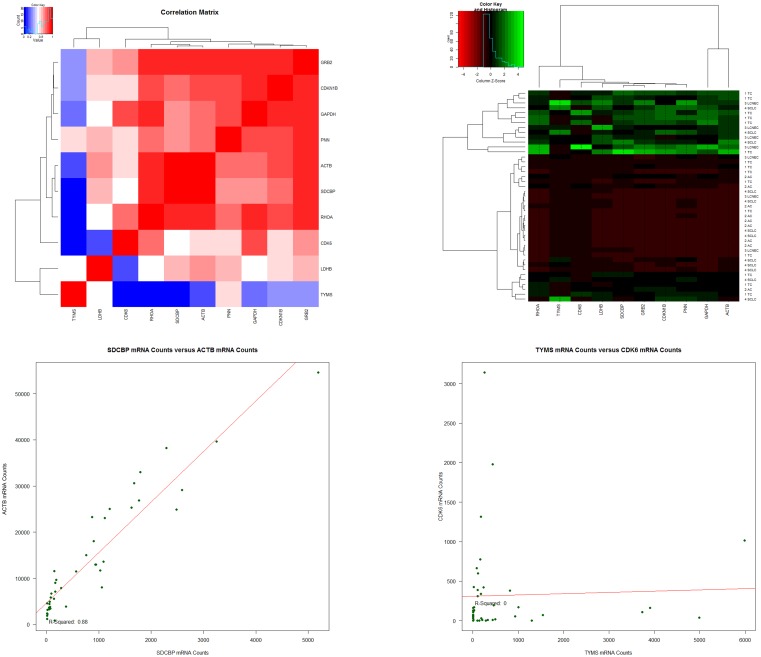
A to D show a correlation matrix for gene expression (A), a heatmap for tumor type versus gene expression (B), scatterplots (C and D) for gene versus gene correlations and R^2^ calculation. **Fig 2A** depicts a correlation matrix of genes that were identified as potential reference genes by geNorm and NormFinder algorithms and previously identified tumor markers (*CDK6* and *TYMS*). High correlations are outlined by red colored squares. Between *CDKN1B*, *GRB2* and *GAPDH* as well as between *ACTB*, *SDCBP* and *RHOA* a high correlation was identified. Low correlations are indicated by blue squares and were found for tumor markers (*CDK6* and *TYMS*) versus reference gene. **Fig 2B** displays a heatmap. On the x-axis the potential reference genes and tumor markers *CDK6* and *TYMS* are shown. On the y-axis the investigated tumor types are depicted. Differential expression was found between tumor types. Though, the reference genes show a constant expression cluster (either low or high) between the samples investigated. The tumor markers present with differential expression between all samples without showing a specific cluster. **Fig 2C** and **2D** are exemplary scatterplots of gene versus gene correlation, which were created to calculate the coefficient of determination (R^2^). **Fig 2C** depicts the highest correlation identified (R^2^ = 0.88) between two potential reference genes (*ACTB* and *SDCBP*). In **D**, the weakest correlation is depicted, which was found between the two tumor markers (*CDK6* and *TYMS*).

After identification of the six most stable reference genes for each algorithm, the coefficient of determination (R^2^) was generated for *ACTB*, *CDKN1B*, *GAPDH*, *GRB2*, *LDHB*, *PNN*, *RHOA* and *SDCBP*. Considering the top-10 of the R^2^-values, *GRB2* (5 times), *RHOA* (4 times), *ACTB* (3 times), *GAPDH* (3 times) and *SDCBP* (3 times) showed the highest correlation frequency. *CDKN1B* and *PNN* showed only one correlation with another gene. Then a correlation matrix was created that confirmed the previous results and also identified *CDKN1B* to have a considerable correlation with *GRB2* and *GAPDH* expression (Fig 2A).

As control, correlation of potential reference gene and suspected tumor markers (*CDK6* and *TYMS*) was performed revealing very low R^2^-values, as expected. The R^2^-values and top-10 ranking are summarized in Table 1. Fig 2A–2C depicts the correlation matrix, a heat map and two exemplary scatterplots of the correlation analysis. The scatterplots for all gene correlations are summarized in S2 Fig.

**Table 1:** Summarizes coefficients of correlation (R-squared) for tested genes and top-10 correlations are highlighted in white.

Ranking	Gene A	Gene B	R-squared
1	*ACTB*	*SDCBP*	0.88
2	*GRB2*	*RHOA*	0.84
3	*ACTB*	*GRB2*	0.84
4	*GRB2*	*SDCBP*	0.82
5	*ACTB*	*RHOA*	0.81
6	*RHOA*	*SDCBP*	0.8
7	*GRB2*	*PNN*	0.8
8	*GAPDH*	*GRB2*	0.79
9	*GAPDH*	*RHOA*	0.78
10	*GAPDH*	*CDKN1B*	0.77
11	*CDKN1B*	*GRB2*	0.76
12	*CDKN1B*	*PNN*	0.74
13	*GAPDH*	*PNN*	0.72
14	*CDKN1B*	*RHOA*	0.71
15	*ACTB*	*CDKN1B*	0.66
16	*GAPDH*	*CDK6*	0.66
17	*ACTB*	*GAPDH*	0.63
18	*CDKN1B*	*SDCBP*	0.63
19	*ACTB*	*PNN*	0.6
20	*RHOA*	*CDK6*	0.59
21	*PNN*	*RHOA*	0.58
22	*ACTB*	*LDHB*	0.53
23	*PNN*	*SDCBP*	0.53
24	*GAPDH*	*SDCBP*	0.52
25	*GRB2*	*CDK6*	0.48
26	*GRB2*	*LDHB*	0.44
27	*LDHB*	*SDCBP*	0.43
28	*LDHB*	*PNN*	0.38
29	*ACTB*	*CDK6*	0.36
30	*PNN*	*CDK6*	0.36
31	*CDKN1B*	*CDK6*	0.34
32	*PNN*	*TYMS*	0.34
33	*CDKN1B*	*LDHB*	0.29
34	*LDHB*	*RHOA*	0.28
35	*LDHB*	*TYMS*	0.26
36	*GAPDH*	*LDHB*	0.25
37	*SDCBP*	*CDK6*	0.25
38	*CDKN1B*	*TYMS*	0.11
39	*GRB2*	*TYMS*	0.09
40	*GAPDH*	*TYMS*	0.07
41	*LDHB*	*CDK6*	0.04
42	*ACTB*	*TYMS*	0.03
43	*SDCBP*	*TYMS*	0.01
44	*CDK6*	*TYMS*	0
45	*RHOA*	*TYMS*	0

Taking the presented results into consideration, *ACTB*, *CDKN1B*, *GAPDH*, *GRB2*, *RHOA* and *SDCBP* can be considered as reliable and stably expressed reference genes in neuroendocrine lung cancer.

## Discussion

Degradation of nucleic acids in FFPE tissue leads to highly degraded RNA and represents an eminent problem for routine diagnostic and research. Formalin fixation leads to RNA integrity numbers <3 [18]. Nevertheless, a large proportion of RNA fragments of ≥100 nt remain intact and can be detected reliably [2, 5]. This fragment length is sufficient to analyze such samples with the nCounter technology, which uses probe pairs binding to ~100 nt of the target regions [3, 5–7]. This approach makes it an ideal method for the analysis of degraded archival FFPE tissue. In the present study, we were able to specifically and sensitively analyze 91 mRNA-target transcripts in 43 FFPE samples by using 100 ng of total RNA per sample. Tumor specimens from 2005 till 2012 were investigated and age of the FFPE sample did not show a statistically significant influence on the results.

Housekeeping genes are no longer referred to as reliable reference genes for normalization [9–11]. For several reference genes the expression is tissue- and disease-specific, therefore leading to heterogeneous ratings and controversial discussions which genes are suitable as internal references [9–11]. According to our results, NormFinder [17] and/or geNorm [9] algorithms plus calculation of the coefficient of determination and matrix correlation identified *ACTB*, *CDKN1B*, *GAPDH*, *GRB2*, *RHOA* and *SDCBP* to be reliable and stably expressed reference genes in neuroendocrine lung cancer. *ACTB* and *GAPDH* were detectable in 100% of cases and the remaining genes were detected in all but one case. As control, the coefficient of determination was also calculated for the reference genes and tumor markers *CDK6* [15] and *TYMS* [14] and showed considerably low correlations between them.

A shortcoming of both algorithms is that they prefer low counts over high counts. Higher counts may lead to a considerably higher deviation. Therefore, very low counts (mean and median <500 mRNA counts) result in M-values indicating high stability/constant expression. But in fact, most of the genes identified as stably expressed ones showed very low counts in the majority of cases (S3 Table). *ACTB* and *GAPDH* gave high counts in all samples, but showed a higher variability. This leads to slightly weaker stability values than found for low expressed genes. Still, *ACTB* and *GAPDH* can be considered reliable reference genes due to the ubiquitous and quiet stable expression as well as high-ranked M-values.

To overcome the limitations of both algorithms, calculation of the coefficient of determination was performed and a correlation matrix was created. The additional tests showed that the six identified reference genes have a high conformity with respect to their expression profiles. Usage of multiple robustly expressed internal references is highly encouraged, because a single reference could lead to false-negative/-positive results due to varying expression [10, 11].

Alternative quantification/normalization methods are under discussion including standardization to cell number and RNA mass quantification (most frequently used in Northern Blot analysis), but these methods are hardly applicable for clinical samples with low RNA quality and unknown cell content [9]. Therefore, normalization to reference genes seems to be the best choice for quantitative mRNA analysis until alternative approaches are found. In the present study, *ACTB*, *CDKN1B*, *GAPDH*, *GRB2*, *RHOA* and *SDCBP* were identified as suitable reference genes in neuroendocrine tumors of the lung.

Based on the presented choice of reference genes, we have identified diagnostic, prognostic and therapy-relevant biomarkers in NELC. With respect to apoptosis and cell cycle, NELC show a distinct expression and mutation pattern. Carcinoids use *CDK4/6* and *CCND1* to drive cell cycle progression by controlling RB1 phosphorylation. On the other hand, carcinomas control this signaling cascade by using the opposite way (*CDK2* and *CCNE1*) [15]. Of note, *TP53* mutations are exclusively found in carcinomas, but not in carcinoids [19] and on the miRNA level NELC subtypes showed significantly different expression of miRNAs regulating proliferation and apoptosis [20].

Besides, our results revealed why the large randomized phase III clinical trial GALES (global analysis of pemetrexed in SCLC extensive stage) failed. Low expression of *FOLR1* and *FPGS*, but high expression of *TYMS* is the predominant phenotype found in SCLC rendering pemetrexed therapy useless in this entity [14]. Similar results were found in squamous cell carcinoma of the lung where high expression of TYMS precludes response to pemetrexed [21].

Furthermore, poor outcome correlated with elevated expression of the transcription factors *SOX11* and *PAX6* [16]. Mediators of angiogenesis (*HIF1A*, *CRHR2*, *KDR* and *FIGF*) correlated with decreased progression-free survival, poor outcome and more aggressive clinical behavior of NELC revealing potential therapy targets for tyrosine-kinase inhibitors and antibodies [7]. Another potent therapy target could be the 26S proteasome, because it showed significantly higher expression in NELC than in benign controls [22]. Several potent proteasome inhibitors are currently tested in clinical phase trials or have received clinical clearance [23].

## Conclusion

FFPE-derived mRNA can be reliably used for expression analysis using the nCounter technology (NanoString). The method uses two specific oligo-probes, which hybridize to ~100 nt in the region of interest, enabling the analysis of the highly degraded nucleic acids. *ACTB*, *CDKN1B*, *GAPDH*, *GRB2*, *RHOA* and *SDCBP* were identified as constantly expressed genes with high stability (M-)values according to geNorm and NormFinder and coefficients of correlation making them suitable reference genes for expression analysis/biological normalization in neuroendocrine tumors of the lung.

## Supporting Information

S1 Fig Boxplots for gene expression versus tumor subtype. S1 Fig shows boxplots for gene expression of potential reference genes, which were identified by either geNorm or NormFinder algorithm, in correlation with the four investigated tumor subtypes. On the x-axis the four investigated tumor subtypes are shown. The y-axis depicts the mRNA counts for each gene. A Kruskal-Wallis test was performed and significantly differential expression is outlined by p-values. In most cases, atypical carcinoids showed much lower expression than the other three entities. Also SCLC showed relatively low counts.
(PDF)

S2 Fig Scatterplots and coefficients of correlation for gene versus gene correlations. S2 Fig depicts scatterplots and the coefficient of correlation for gene versus gene correlation. Potential reference genes were identified by applying the geNorm and NormFinder algorithm. Potential reference genes were correlated and the coefficient of determination (R^2^) was calculated to identify the conformity between them. As control, tumor markers (*CDK6* and *TYMS*) were included in the analysis and showed very low correlation with the potential reference genes.
(PDF)

S1 Table The gene name, gene ID and properties of the custom CodeSet that was used for the nCounter analysis. (XLS)

S2 Table Patient data, RNA concentration and quality (RIN) and exemplarily gene mRNA counts. (XLSX)

S3 Table Summarizes gene names, calculated stability values by NormFinder or geNorm algorithm, mean and median mRNA counts per gene. Grey stained rows indicate genes that show mean and/or median expression of >500 mRNA counts. Median for all genes is 99 counts and mean for all genes is 1,132 counts.
(XLSX)
