# Supplementary material for: ACTB, CDKN1B, GAPDH, GRB2, RHOA and SDCBP Were Identified as Reference Genes in Neuroendocrine Lung Cancer via the nCounter Technology
Source: PLoS One. 2016 Nov 1;11(11):e0165181. doi: 10.1371/journal.pone.0165181 (PMC5089548; doi:10.1371/journal.pone.0165181)
Supplement: S1 Fig — shows boxplots for gene expression of potential reference genes, which were identified by either geNorm or NormFinder algorithm, in correlation with the four investigated tumor subtypes. On the x-axis the four investigated tumor subtypes are shown. The y-axis depicts the mRNA counts for each gene. A Kruskal-Wallis test was performed and significantly differential expression is outlined by p-values. In most cases, atypical carcinoids showed much lower expression than the other three entities. Also SCLC showed relatively low counts. (PDF) [file pone.0165181.s001.pdf]

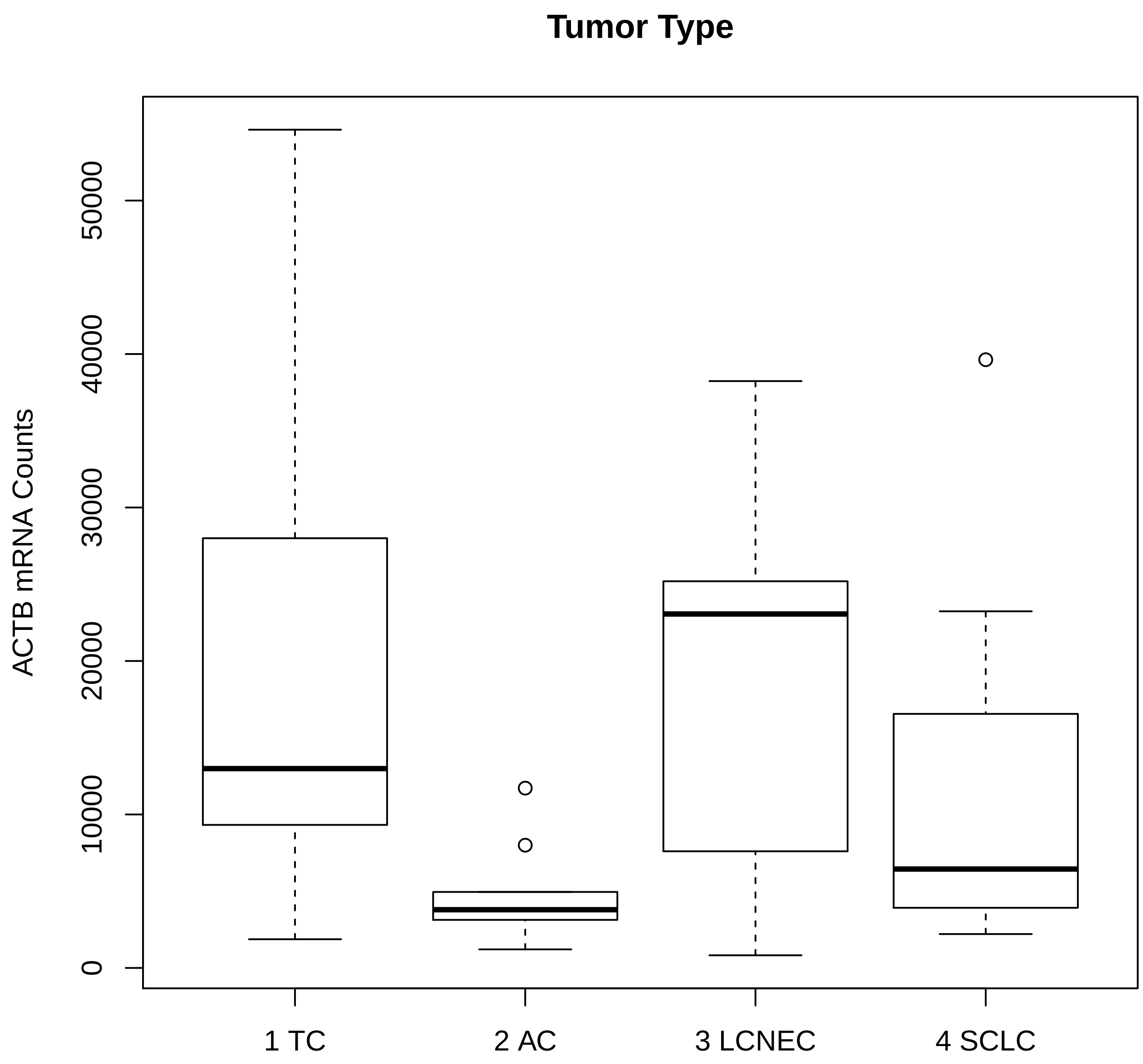
Tumor Type
ACTB mRNA Counts
0
10000
20000
30000
40000
50000
1 TC
2 AC
3 LCNEC
4 SCLC

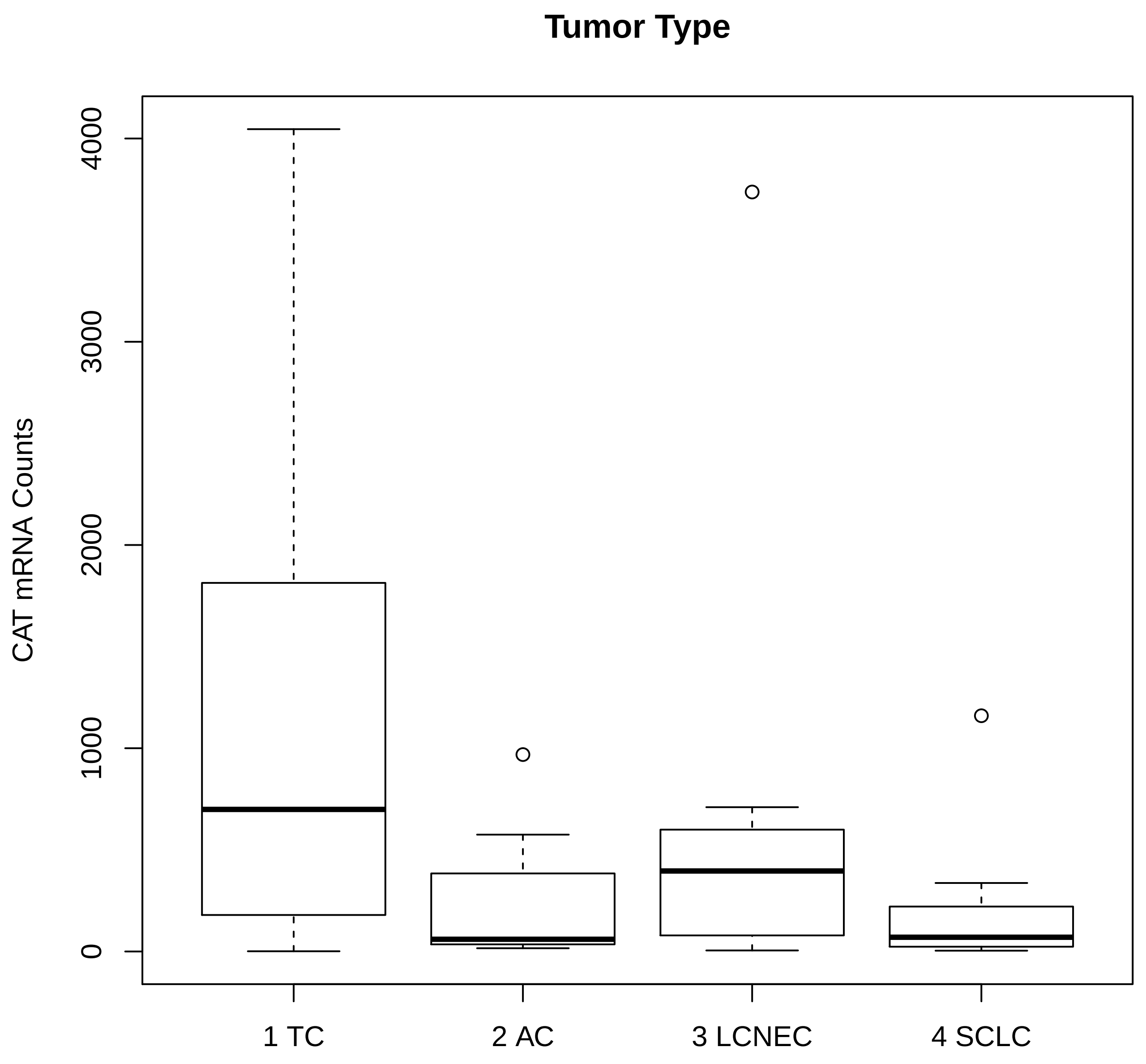

Tumor Type
CAT mRNA Counts
0
1000
2000
3000
4000
1 TC
2 AC
3 LCNEC
4 SCLC

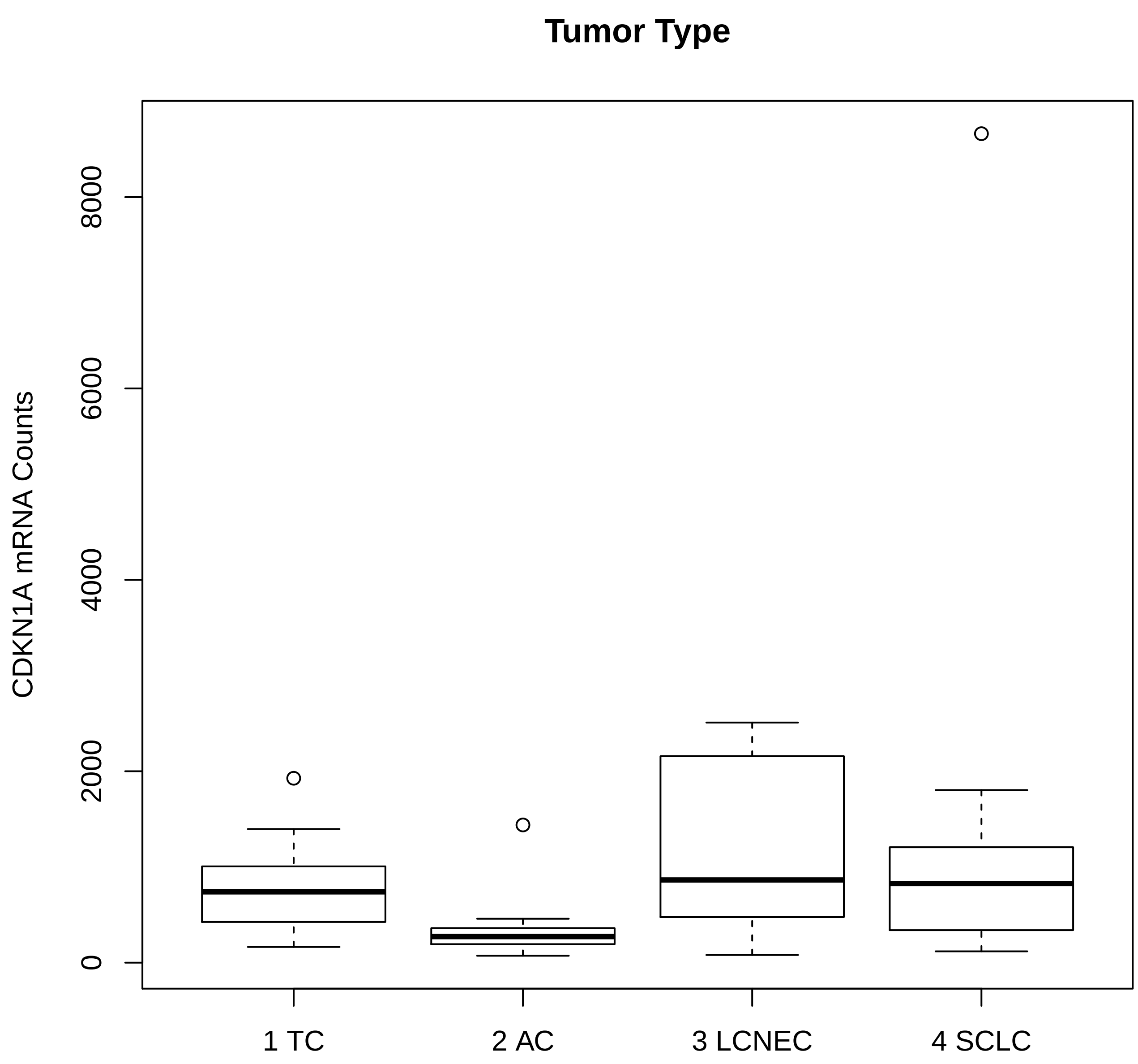
Tumor Type
CDKN1A mRNA Counts
0
2000
4000
6000
8000
1 TC
2 AC
3 LCNEC
4 SCLC

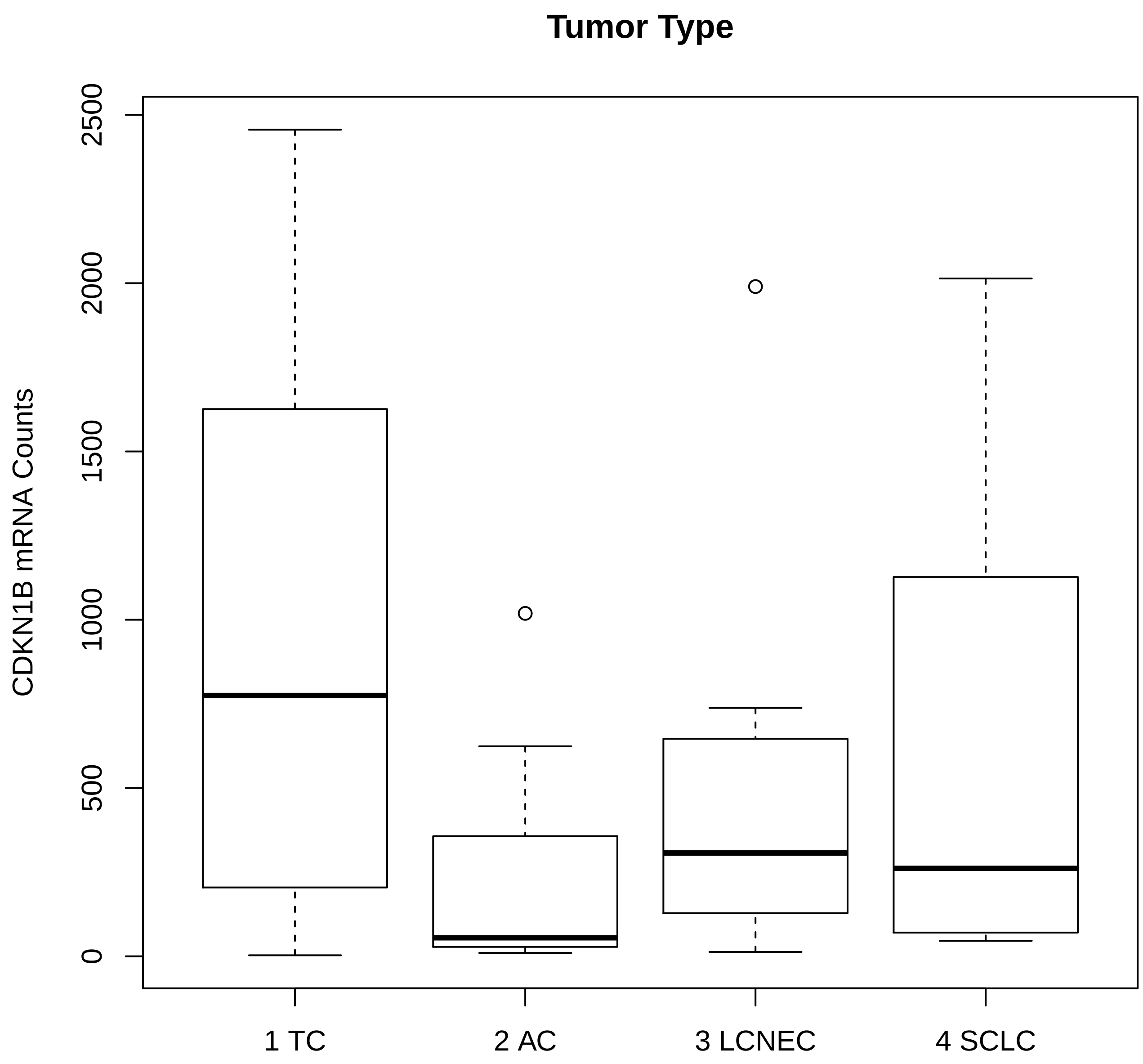
Tumor Type
CDKN1B mRNA Counts
0
500
1000
1500
2000
2500
1 TC
2 AC
3 LCNEC
4 SCLC

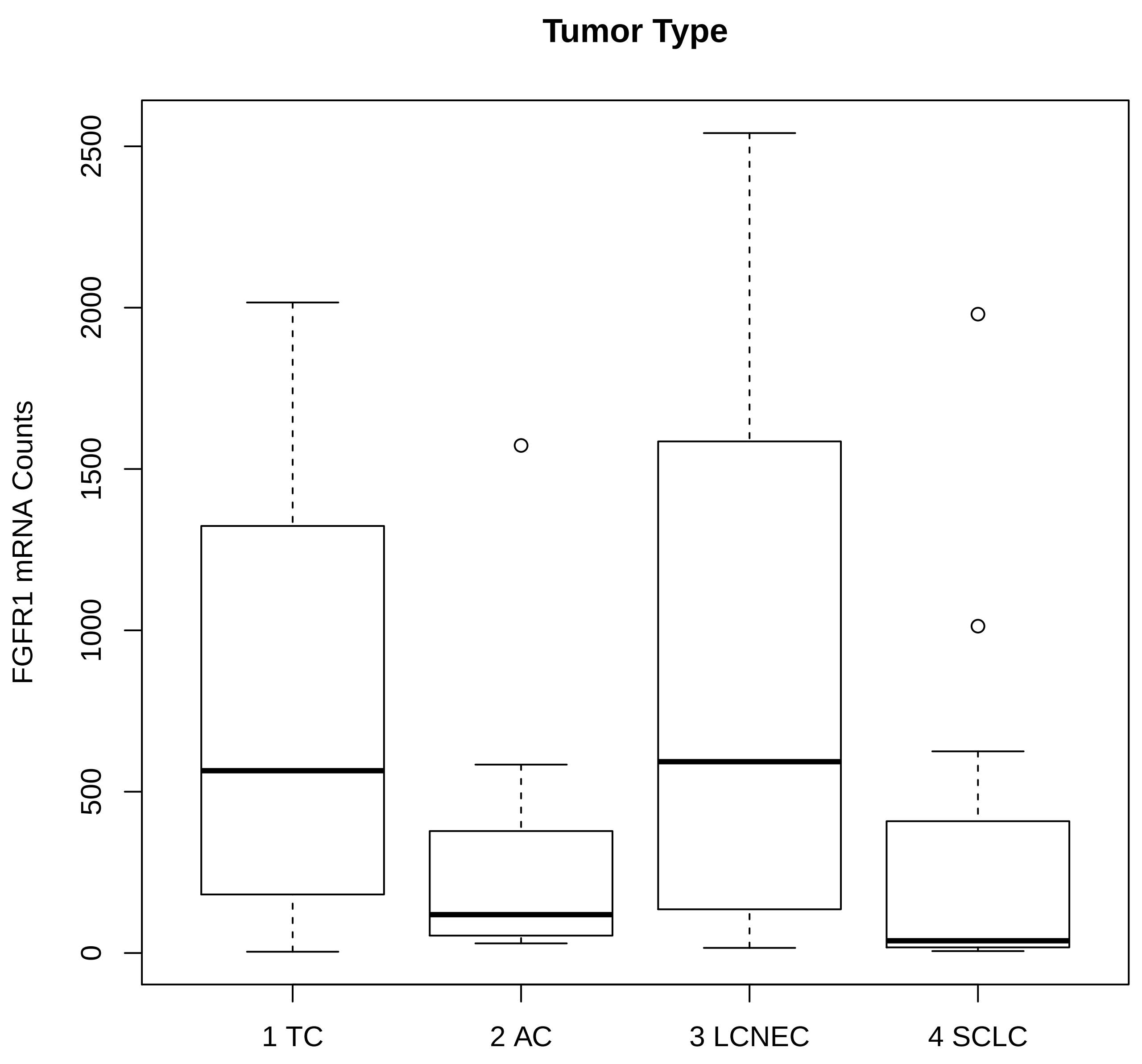
Tumor Type
FGFR1 mRNA Counts
0
500
1000
1500
2000
2500
1 TC
2 AC
3 LCNEC
4 SCLC

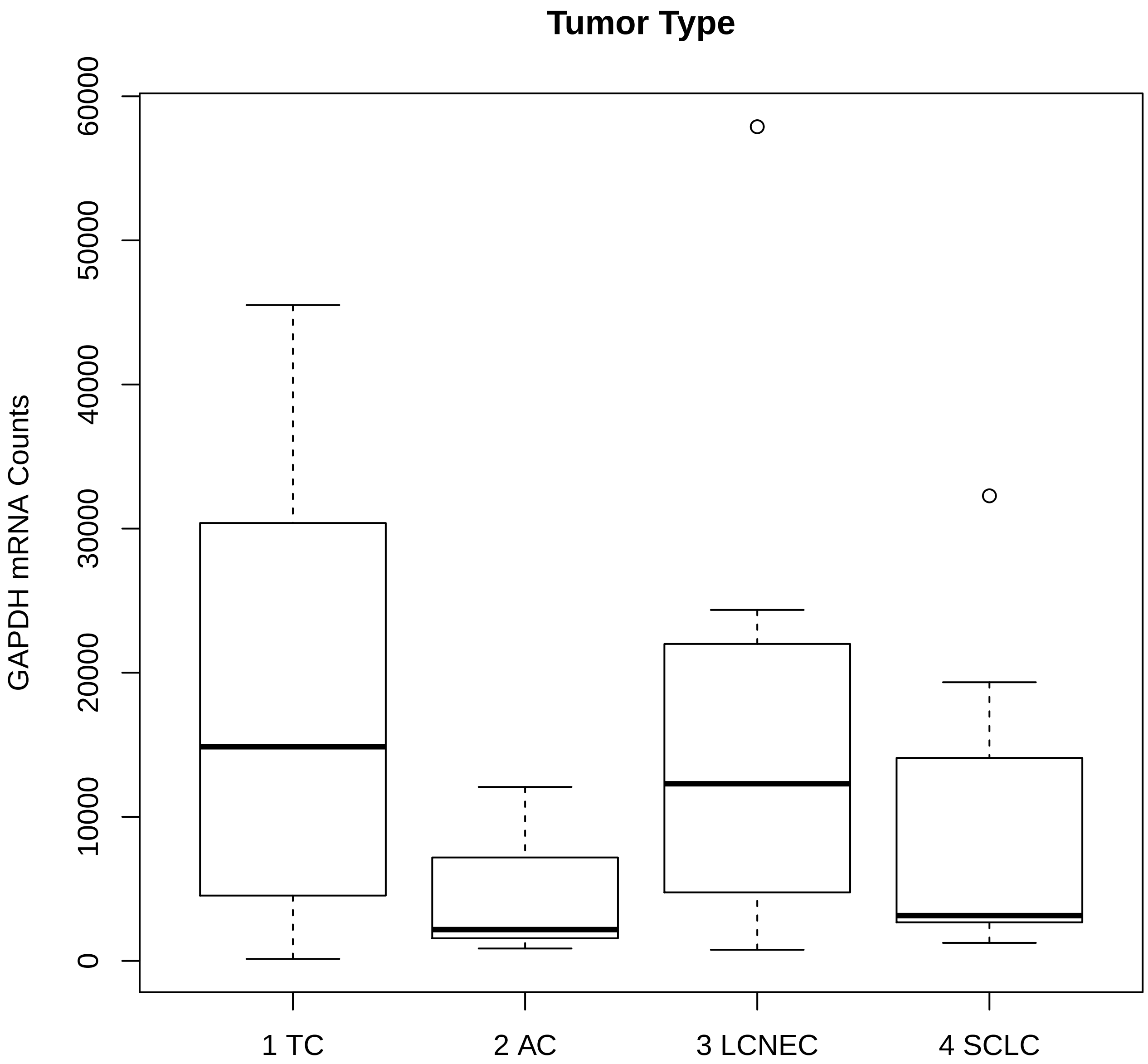
Tumor Type
GAPDH mRNA Counts
0
10000
20000
30000
40000
50000
60000
1 TC
2 AC
3 LCNEC
4 SCLC

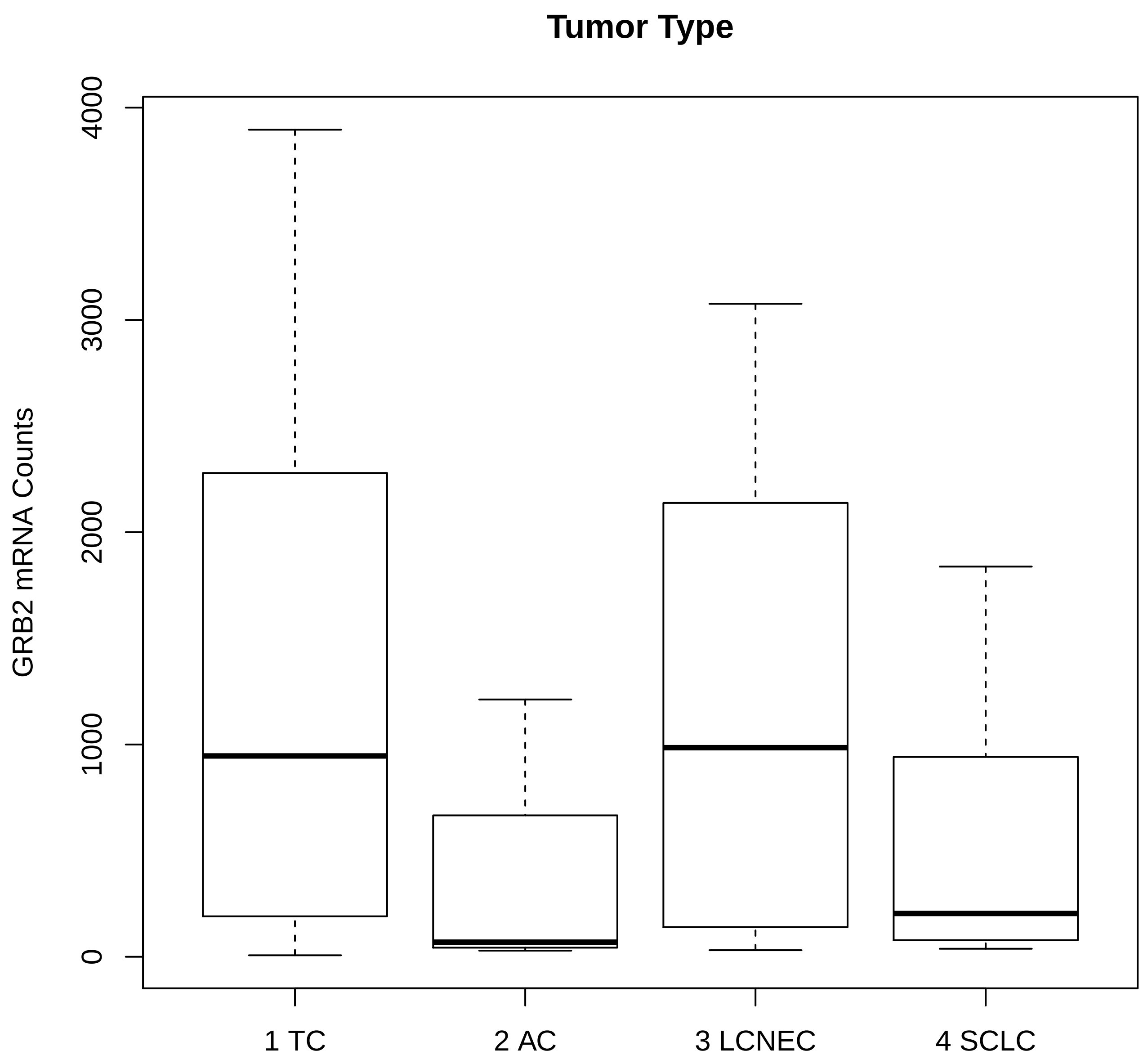

Tumor Type
GRB2 mRNA Counts
0
1000
2000
3000
4000
1 TC
2 AC
3 LCNEC
4 SCLC

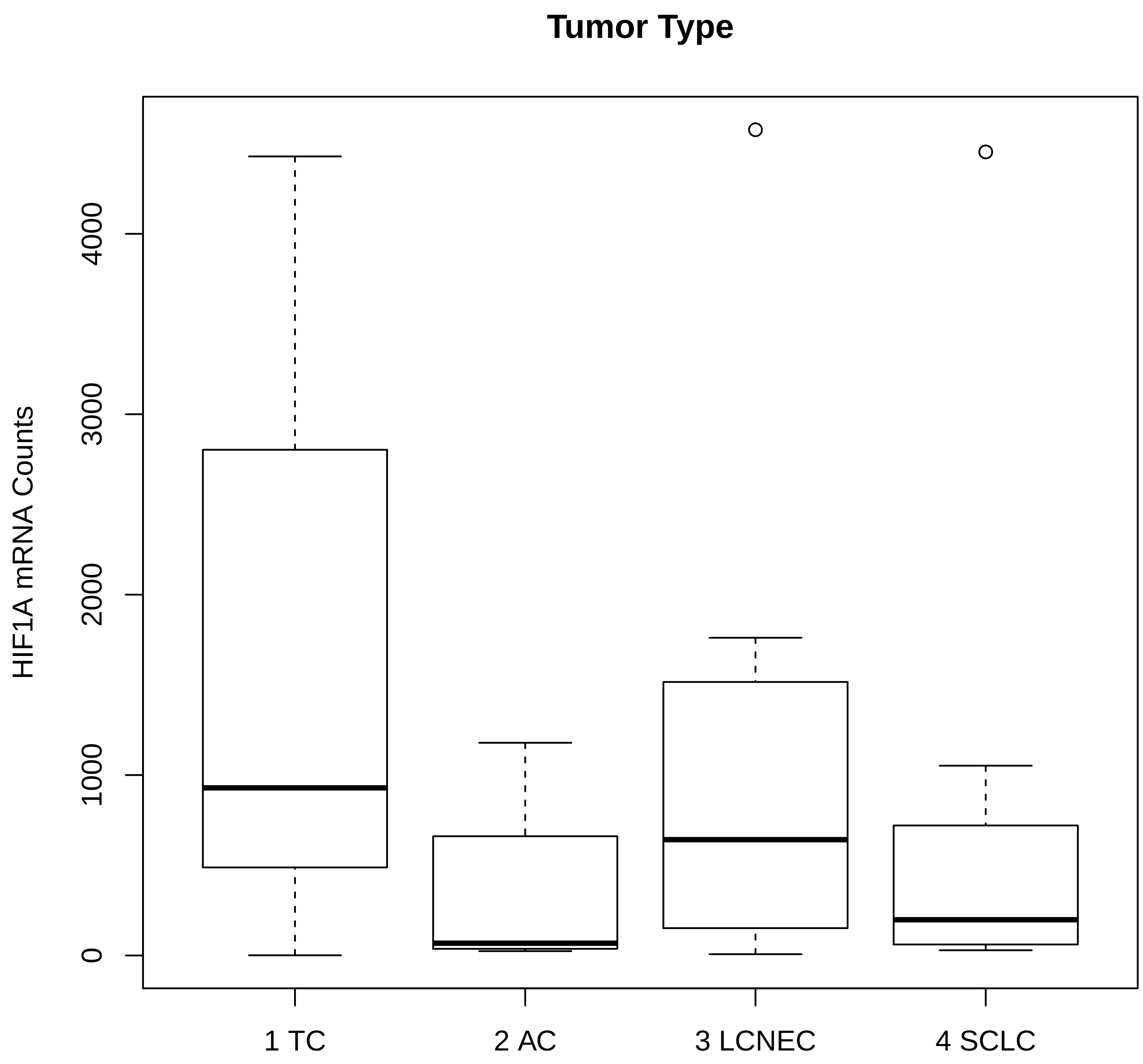
Tumor Type
HIF1A mRNA Counts
0
1000
2000
3000
4000
1 TC
2 AC
3 LCNEC
4 SCLC

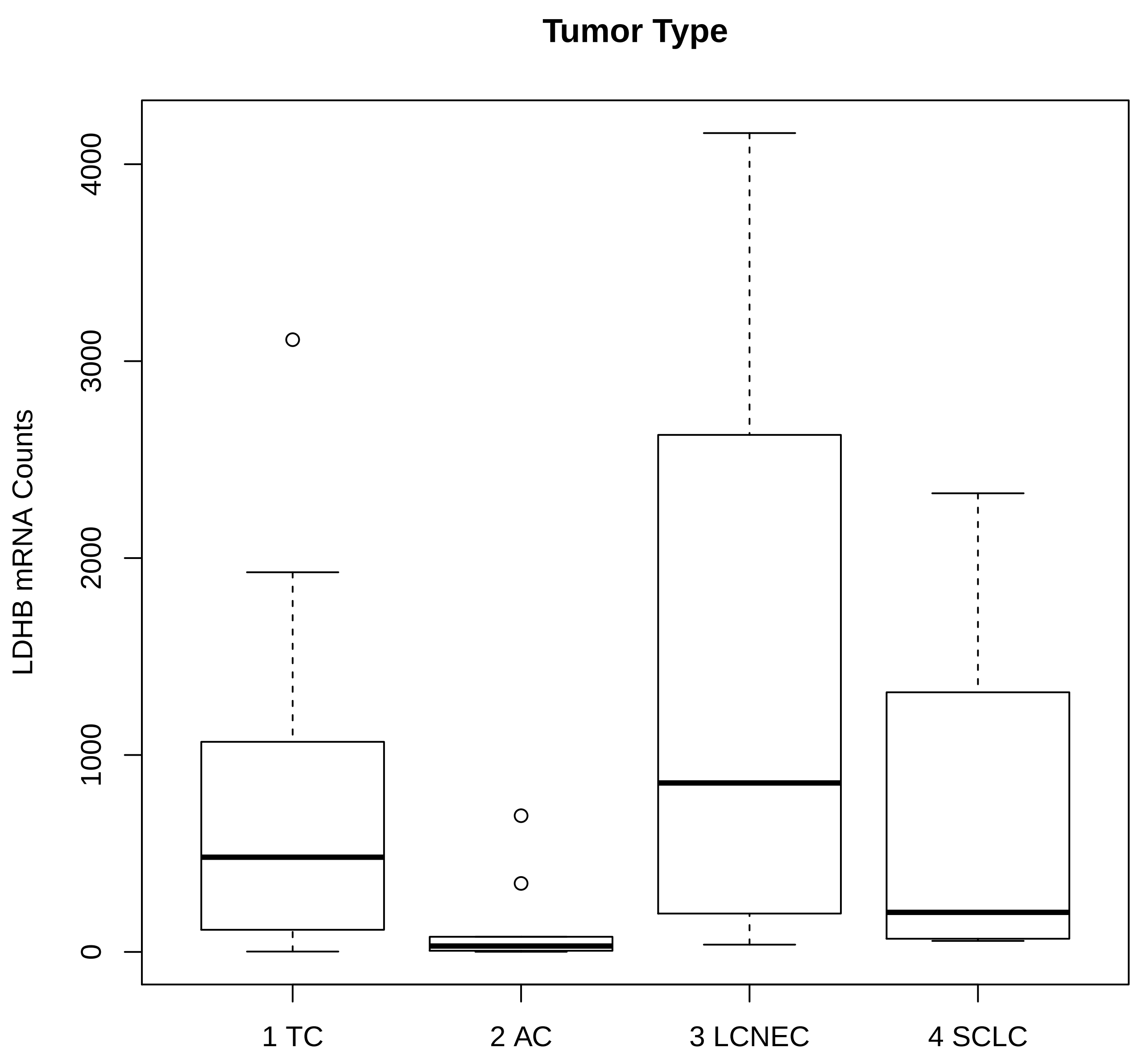
Tumor Type
LDHB mRNA Counts
0
1000
2000
3000
4000
1 TC
2 AC
3 LCNEC
4 SCLC

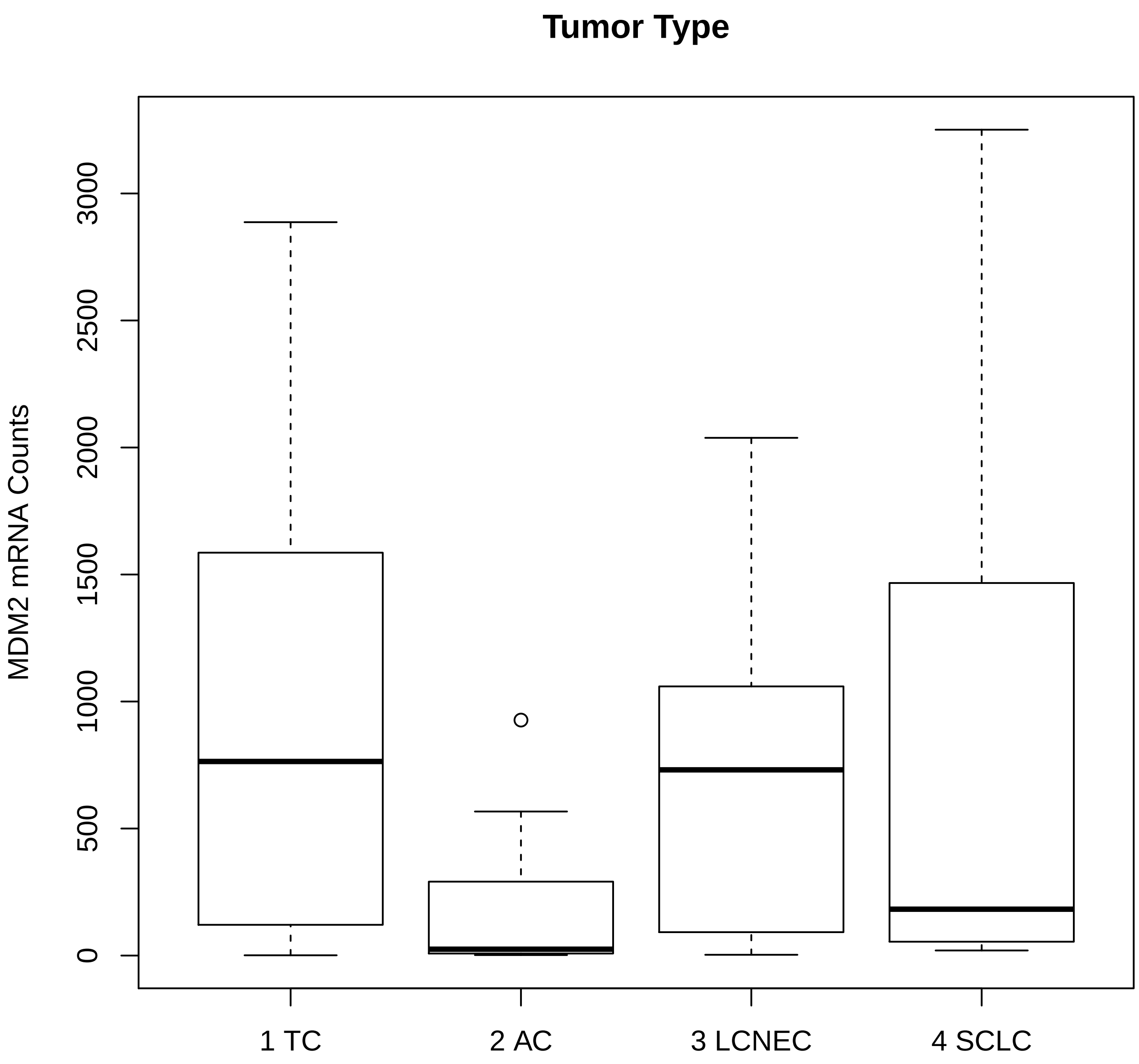

Tumor Type
MDM2 mRNA Counts
0
500
1000
1500
2000
2500
3000
1 TC
2 AC
3 LCNEC
4 SCLC

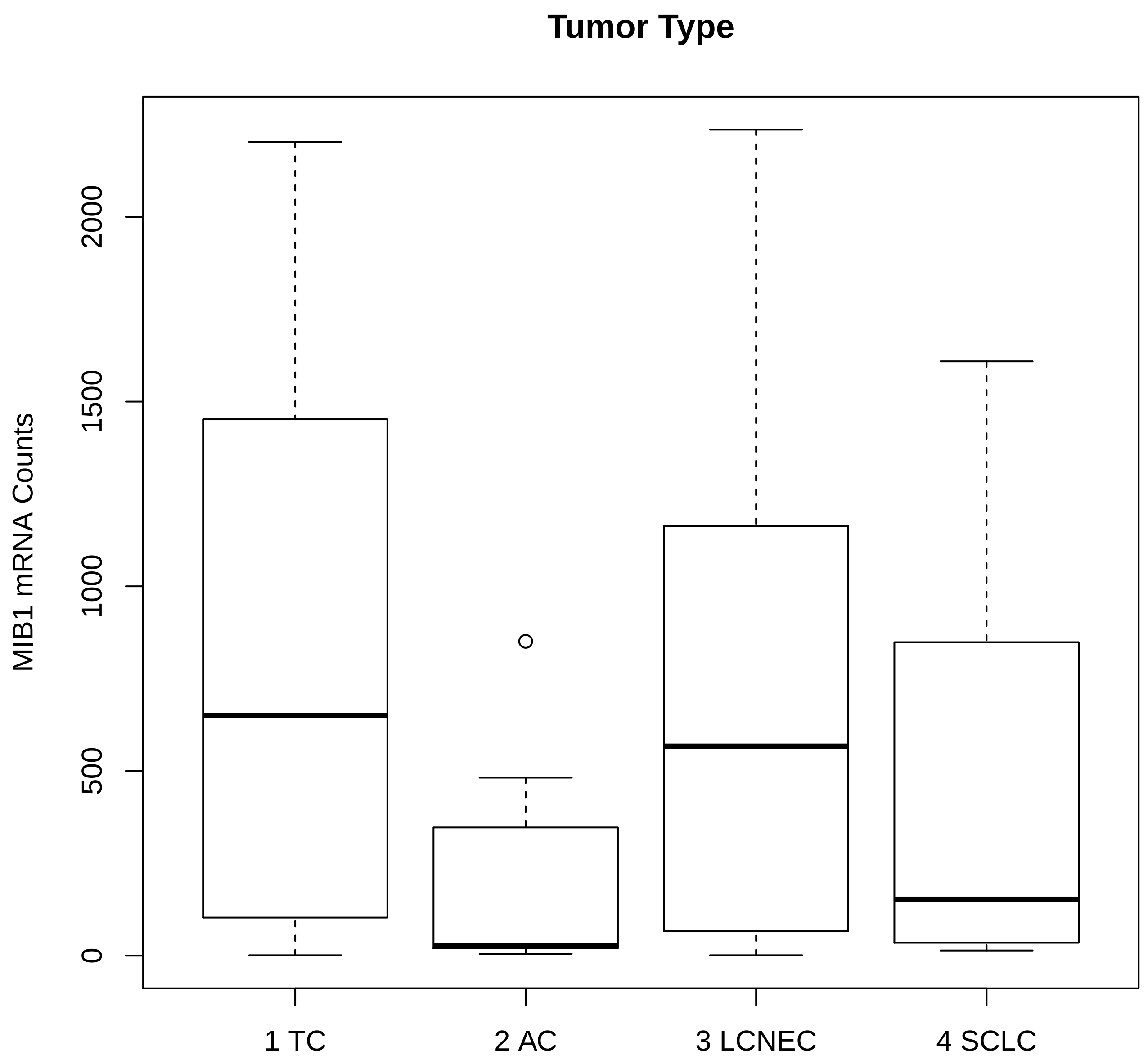
Tumor Type
MIB1 mRNA Counts
0
500
1000
1500
2000
1 TC
2 AC
3 LCNEC
4 SCLC

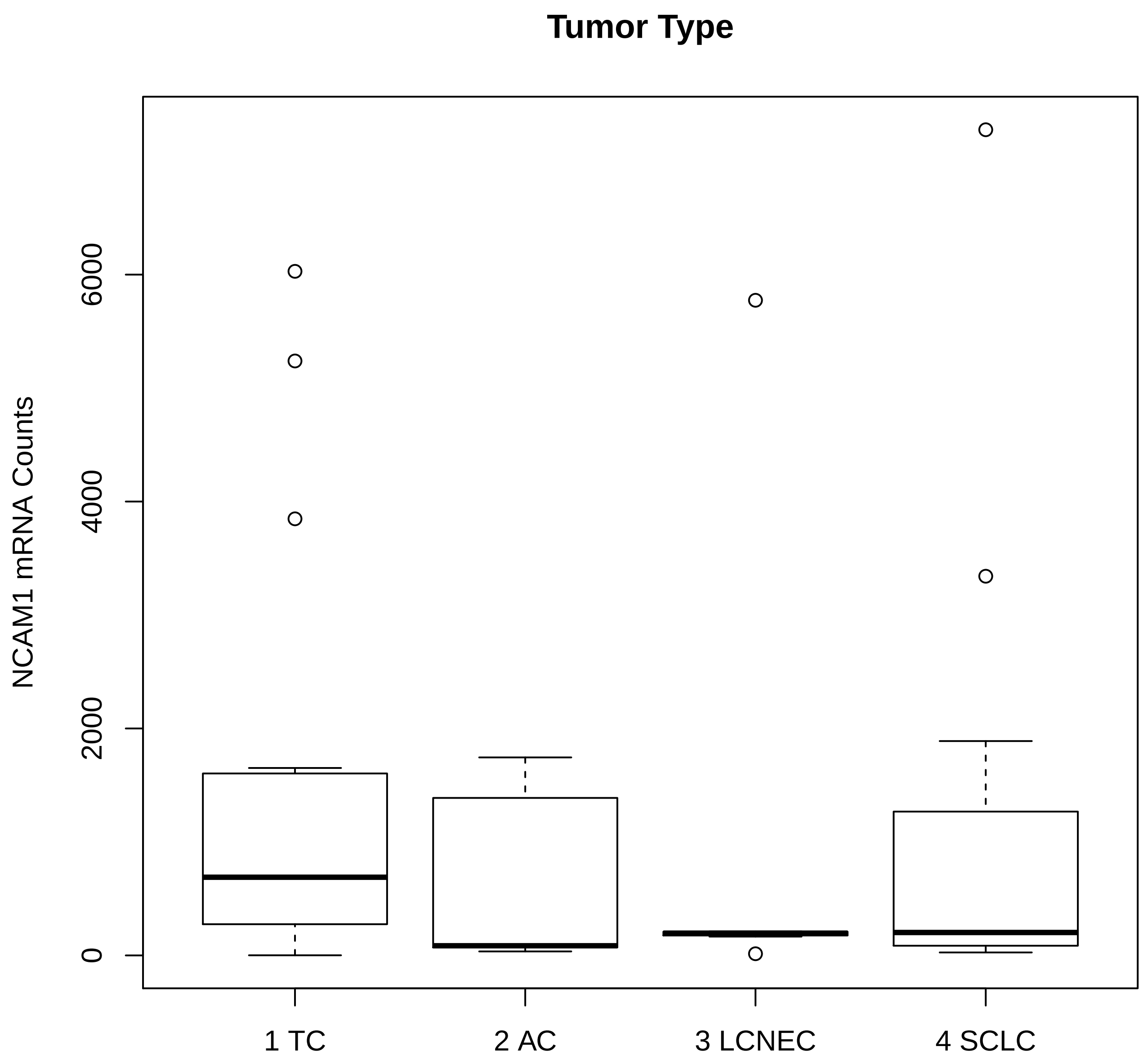

Tumor Type
NCAM1 mRNA Counts
0
2000
4000
6000
1 TC
2 AC
3 LCNEC
4 SCLC

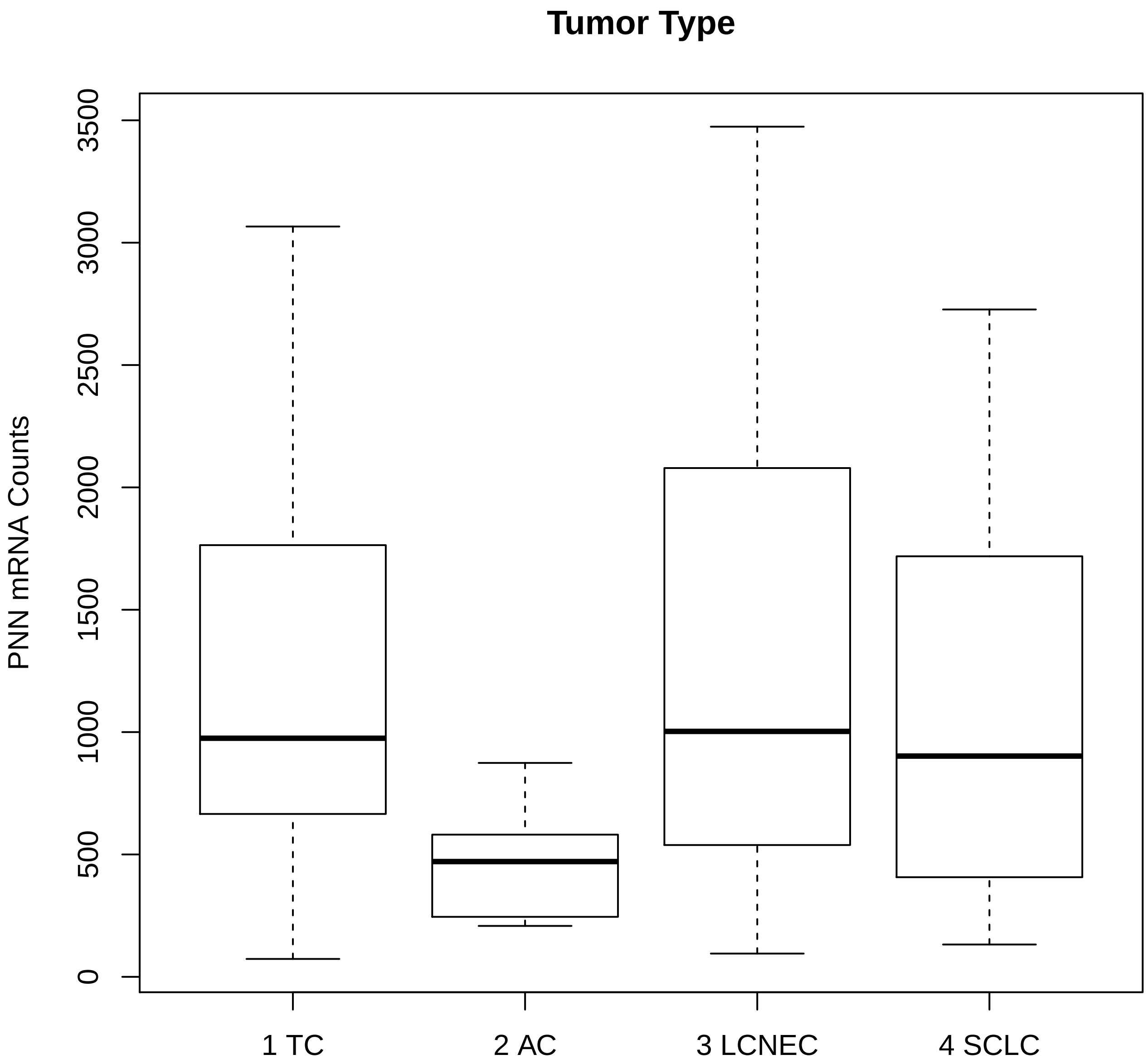

Tumor Type
PNN mRNA Counts
0
500
1000
1500
2000
2500
3000
3500
1 TC
2 AC
3 LCNEC
4 SCLC

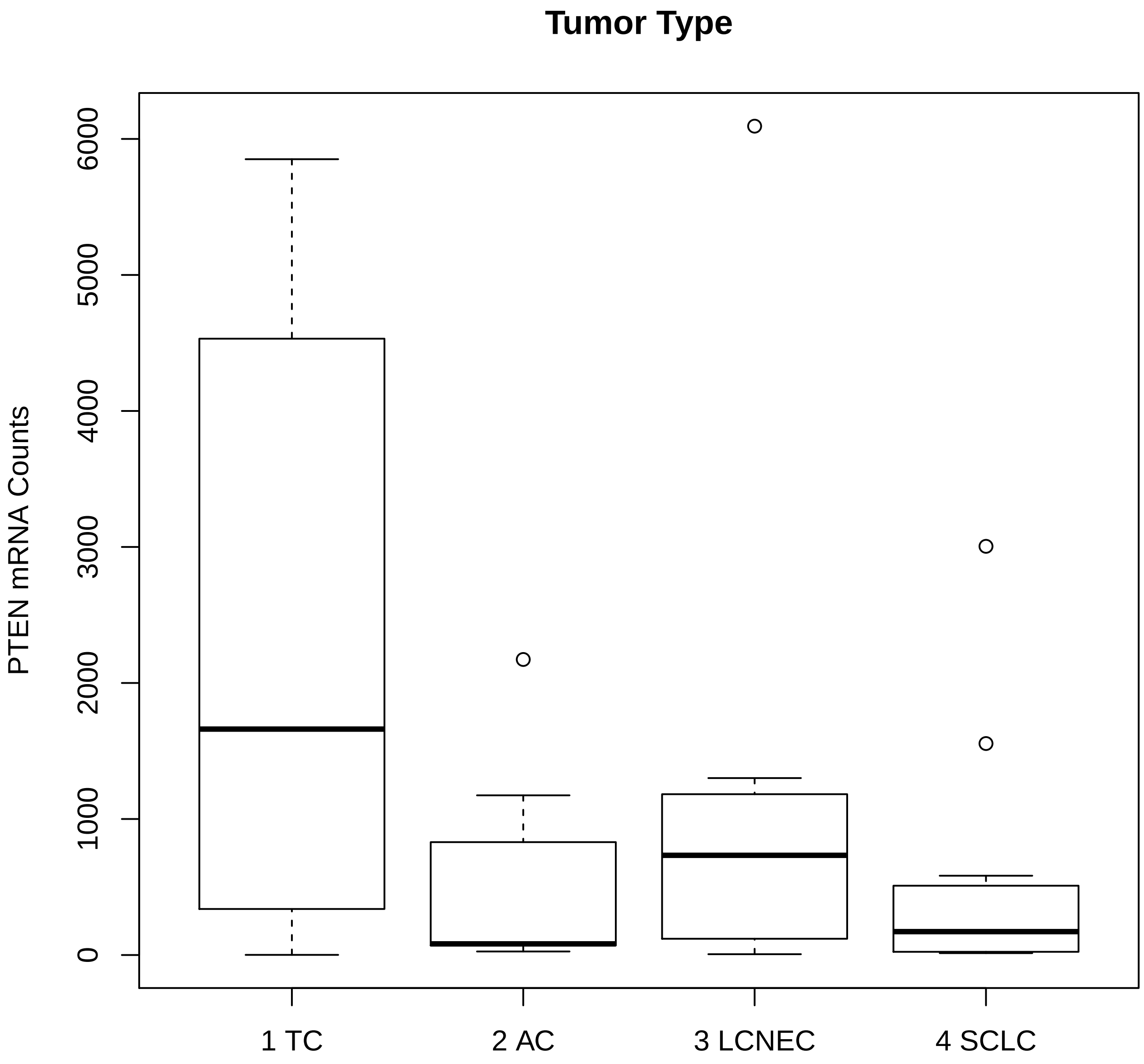

Tumor Type
PTEN mRNA Counts
0
1000
2000
3000
4000
5000
6000
1 TC
2 AC
3 LCNEC
4 SCLC

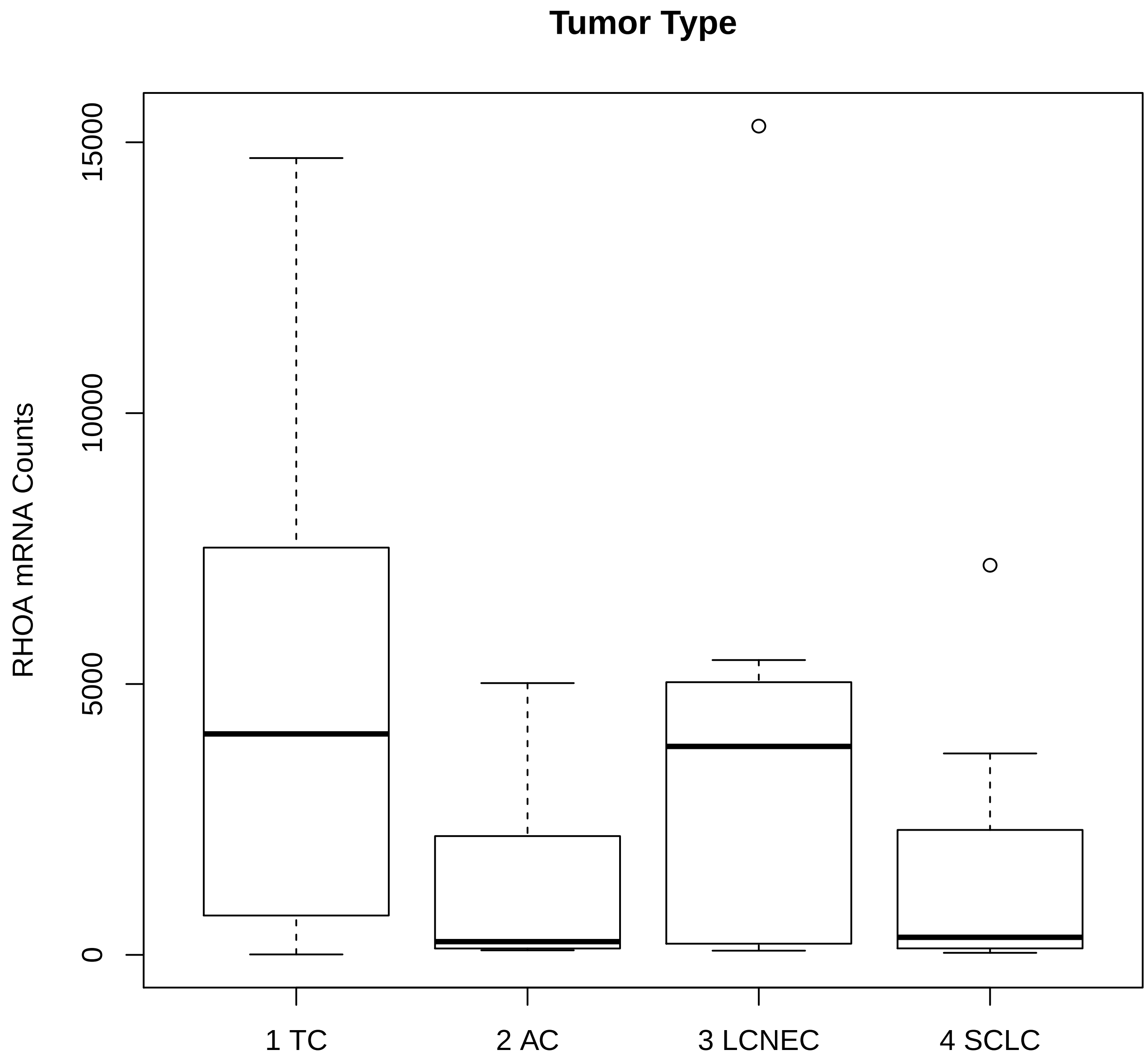
Tumor Type
RHOA mRNA Counts
15000
10000
5000
0
1 TC
2 AC
3 LCNEC
4 SCLC

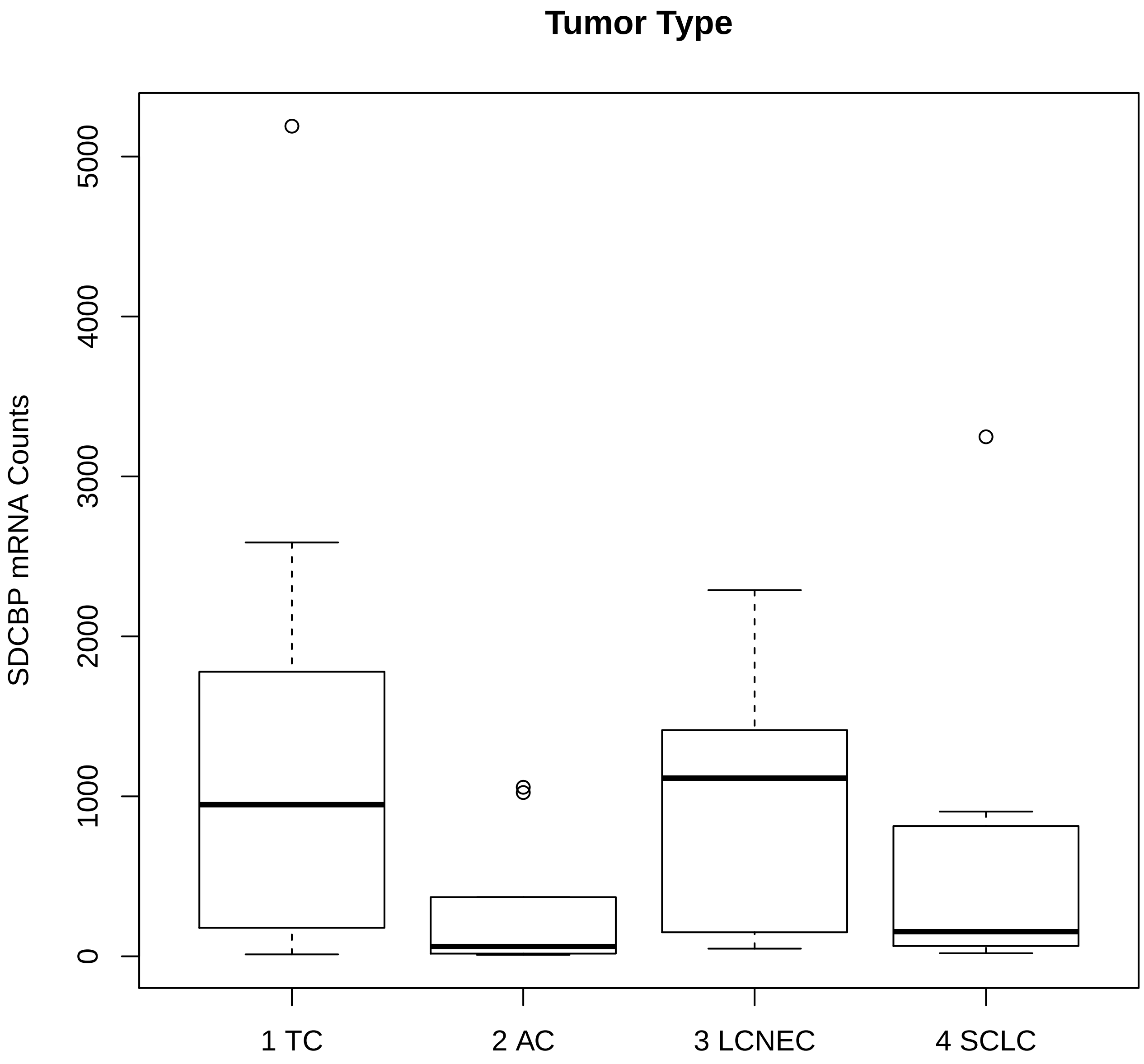
Tumor Type
SDCBP mRNA Counts
0
1000
2000
3000
4000
5000
1 TC
2 AC
3 LCNEC
4 SCLC

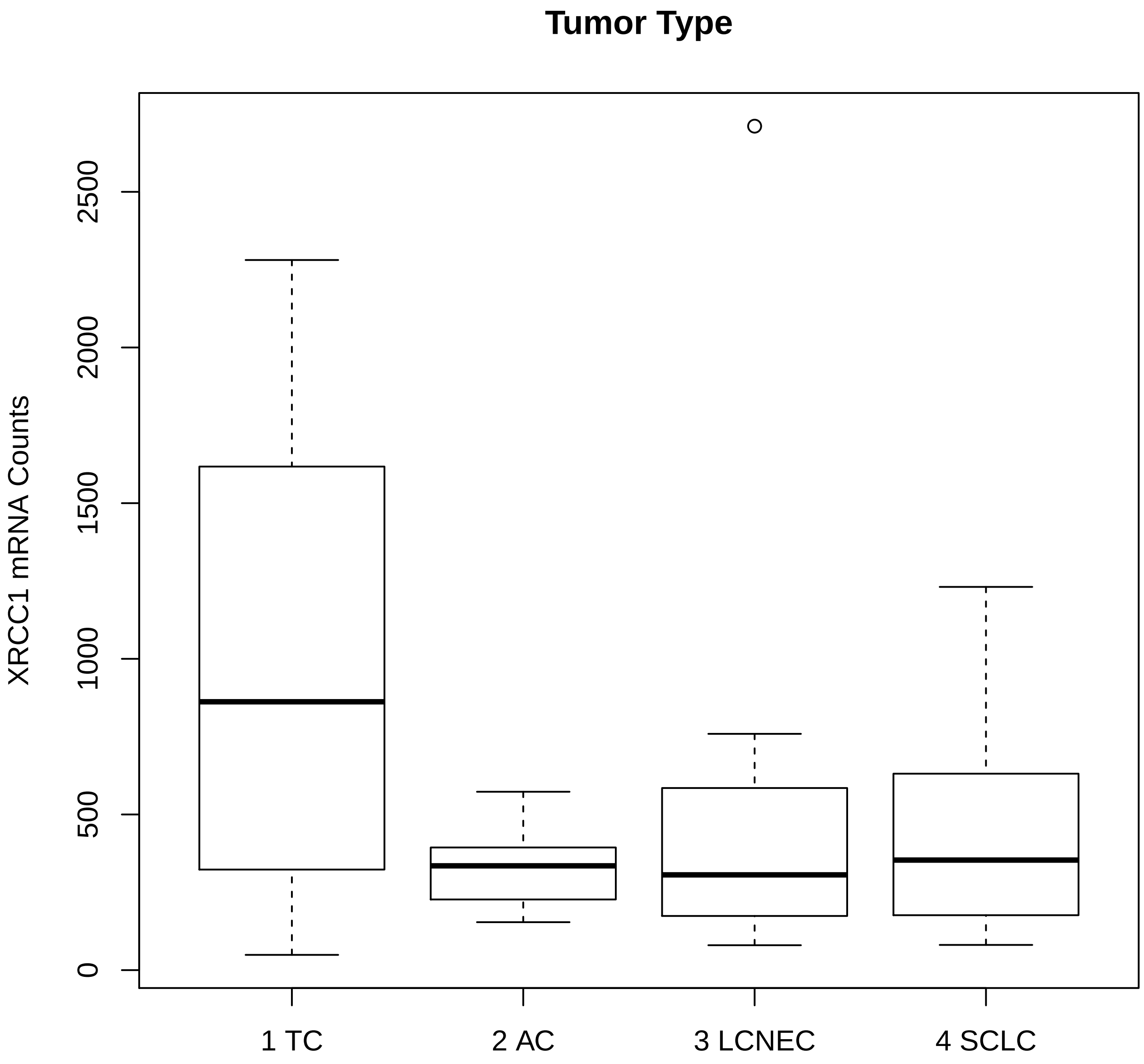
Tumor Type
XRCC1 mRNA Counts
0
500
1000
1500
2000
2500
1 TC
2 AC
3 LCNEC
4 SCLC
