# Supplementary material for: ACTB, CDKN1B, GAPDH, GRB2, RHOA and SDCBP Were Identified as Reference Genes in Neuroendocrine Lung Cancer via the nCounter Technology
Source: PLoS One. 2016 Nov 1;11(11):e0165181. doi: 10.1371/journal.pone.0165181 (PMC5089548; doi:10.1371/journal.pone.0165181)
Supplement: S2 Fig — depicts scatterplots and the coefficient of correlation for gene versus gene correlation. Potential reference genes were identified by applying the geNorm and NormFinder algorithm. Potential reference genes were correlated and the coefficient of determination (R2) was calculated to identify the conformity between them. As control, tumor markers (CDK6 and TYMS) were included in the analysis and showed very low correlation with the potential reference genes. (PDF) [file pone.0165181.s002.pdf]

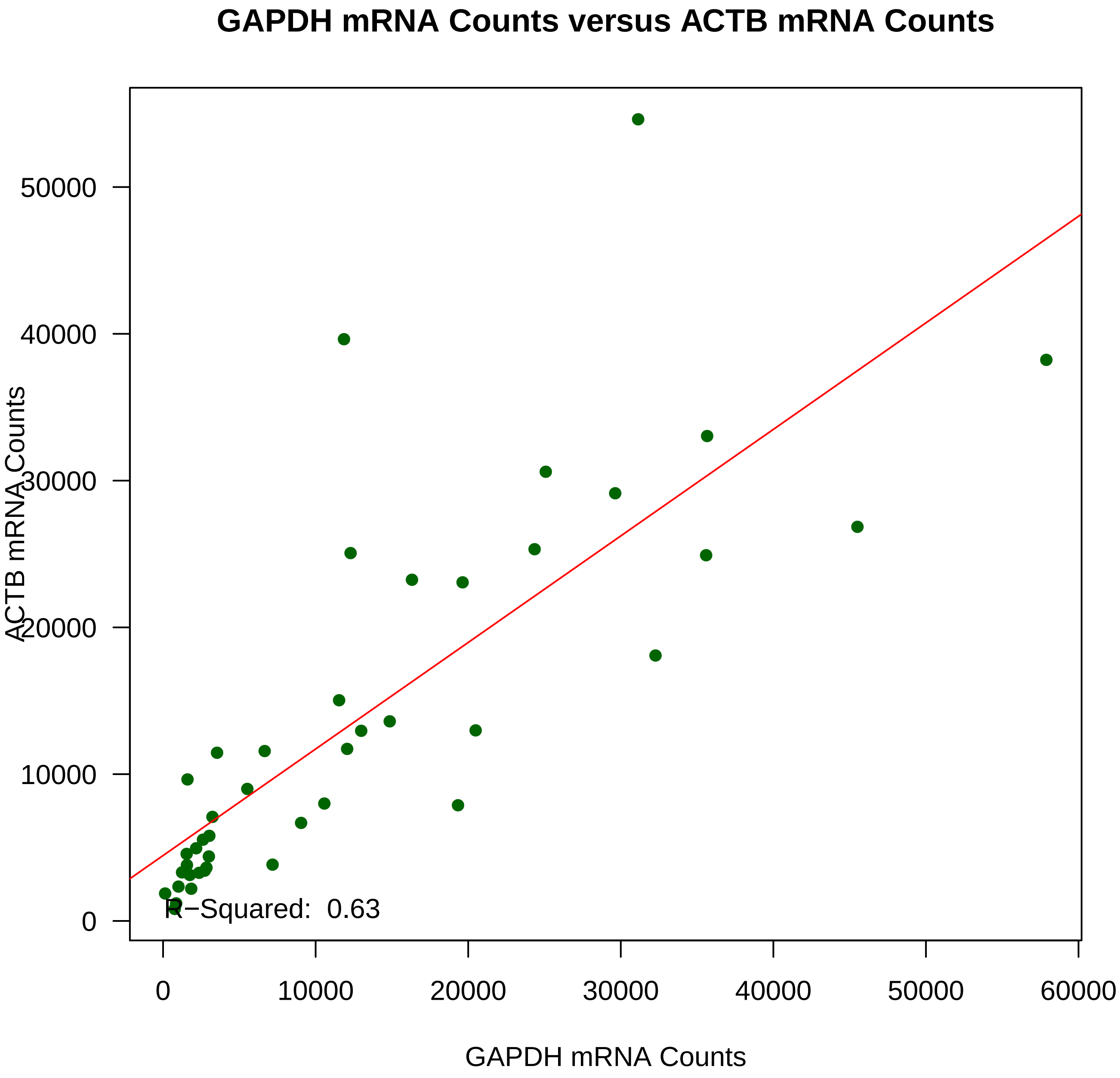
GAPDH mRNA Counts versus ACTB mRNA Counts
ACTB mRNA Counts
GAPDH mRNA Counts
R−Squared: 0.63
0
10000
20000
30000
40000
50000
60000

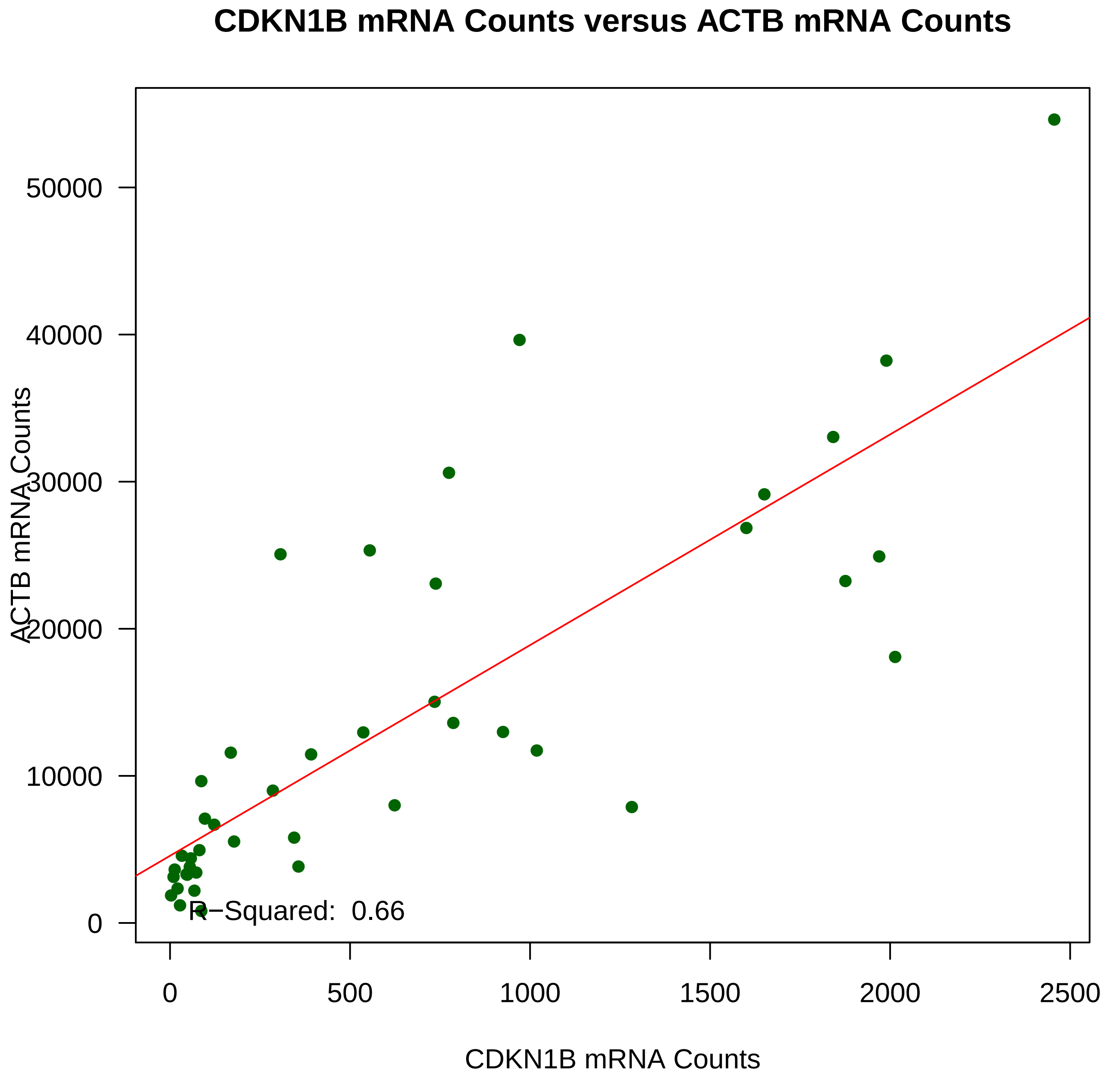

CDKN1B mRNA Counts versus ACTB mRNA Counts
50000
40000
30000
20000
10000
0
ACTB mRNA Counts
R−Squared: 0.66
0
500
1000
1500
2000
2500
CDKN1B mRNA Counts

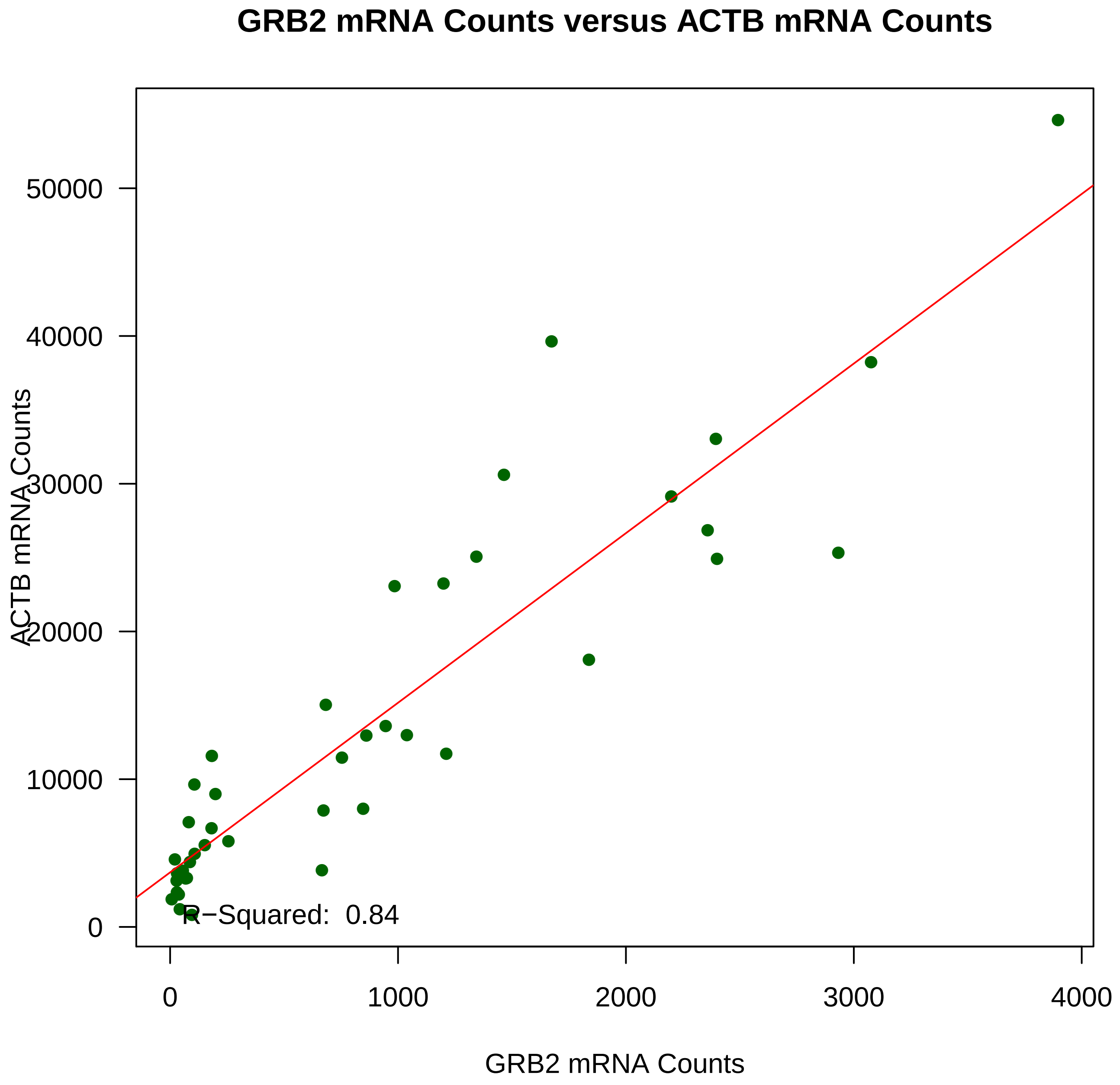

GRB2 mRNA Counts versus ACTB mRNA Counts
ACTB mRNA Counts
0
10000
20000
30000
40000
50000
0
1000
2000
3000
4000
GRB2 mRNA Counts
R−Squared: 0.84

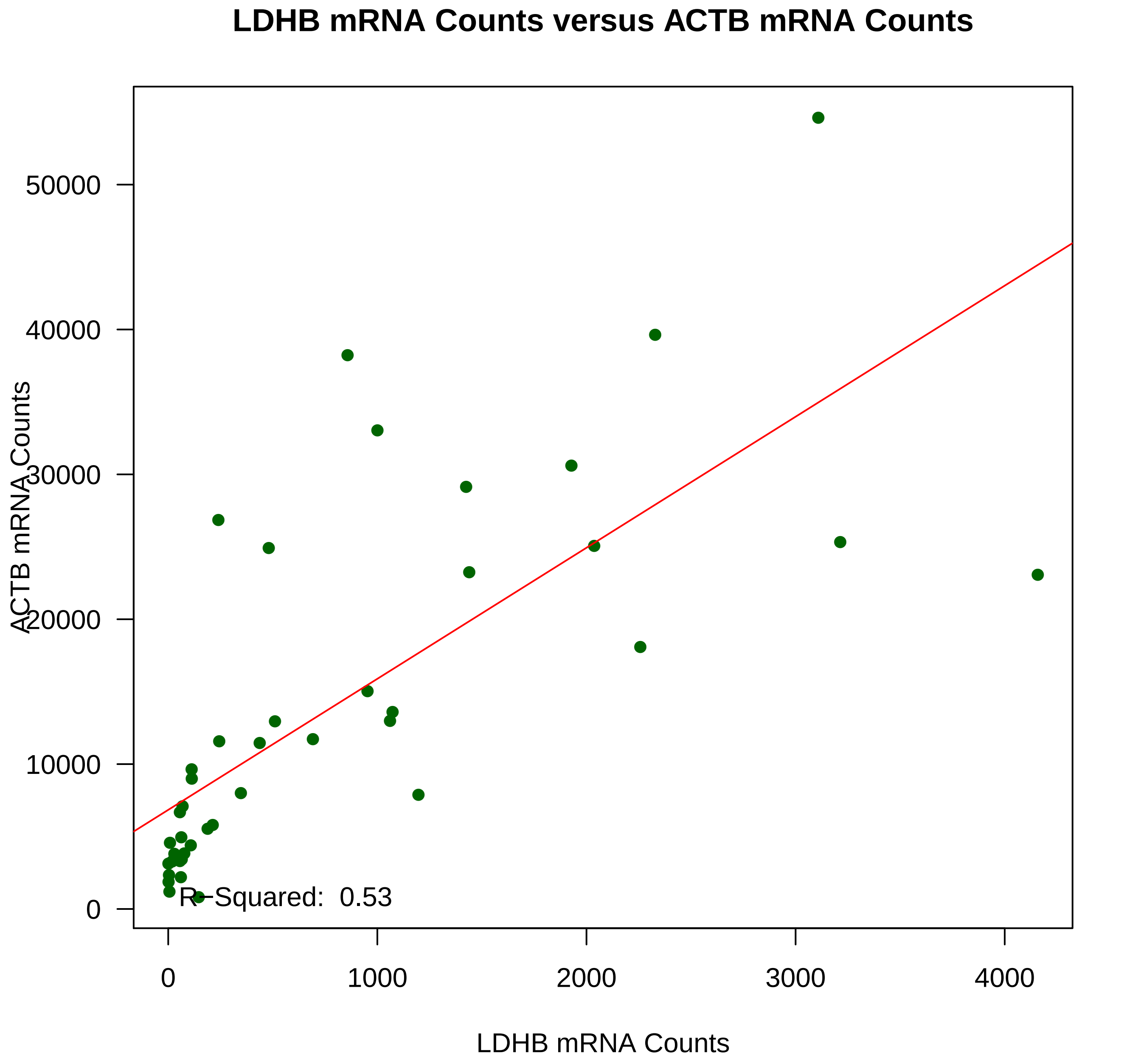

LDHB mRNA Counts versus ACTB mRNA Counts
ACTB mRNA Counts
LDHB mRNA Counts
0
10000
20000
30000
40000
50000
0
1000
2000
3000
4000
R−Squared: 0.53

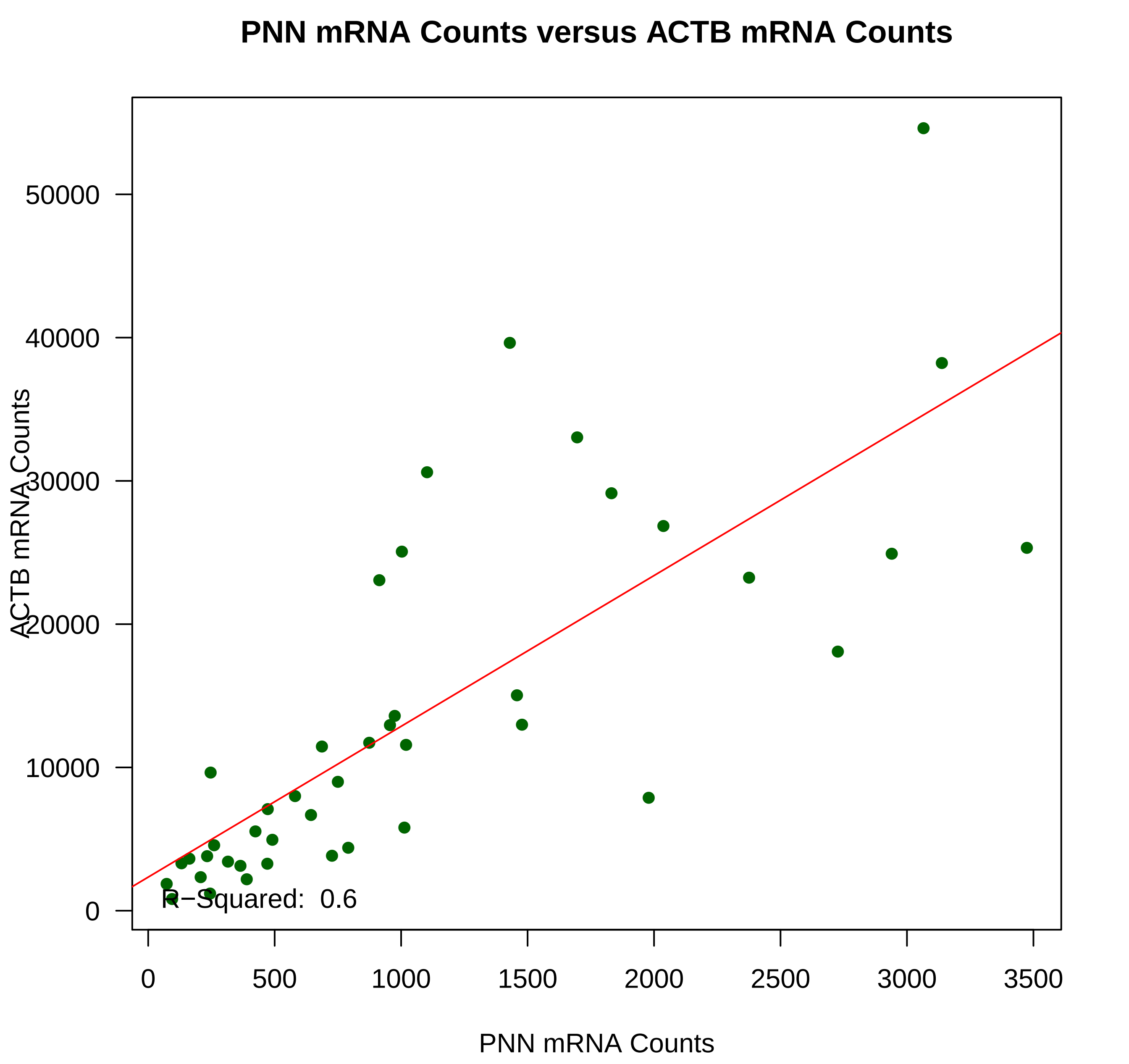
PNN mRNA Counts versus ACTB mRNA Counts
ACTB mRNA Counts
PNN mRNA Counts
0
10000
20000
30000
40000
50000
0
500
1000
1500
2000
2500
3000
3500
R−Squared: 0.6

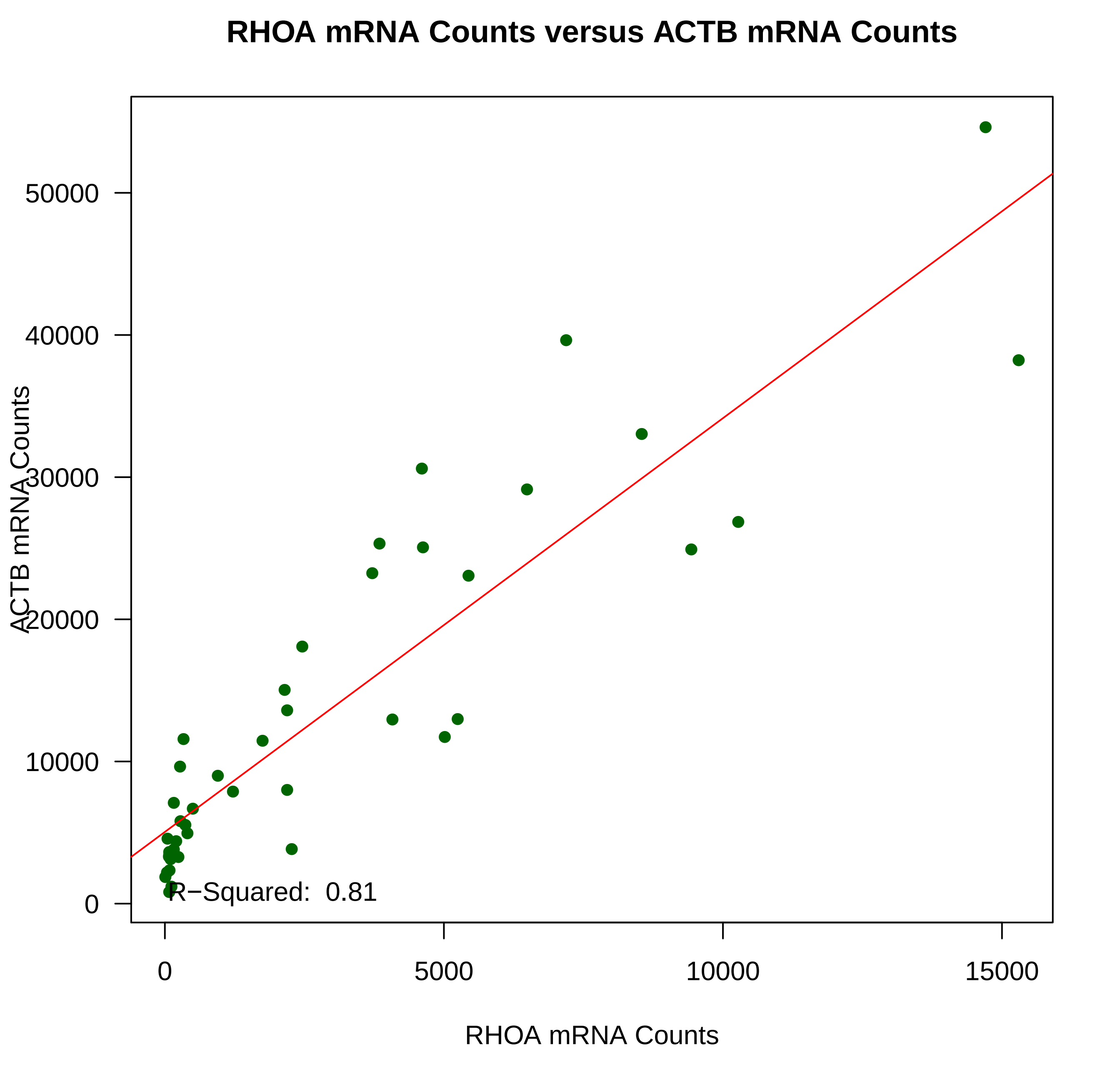
RHOA mRNA Counts versus ACTB mRNA Counts
ACTB mRNA Counts
RHOA mRNA Counts
0
10000
20000
30000
40000
50000
0
5000
10000
15000
R−Squared: 0.81

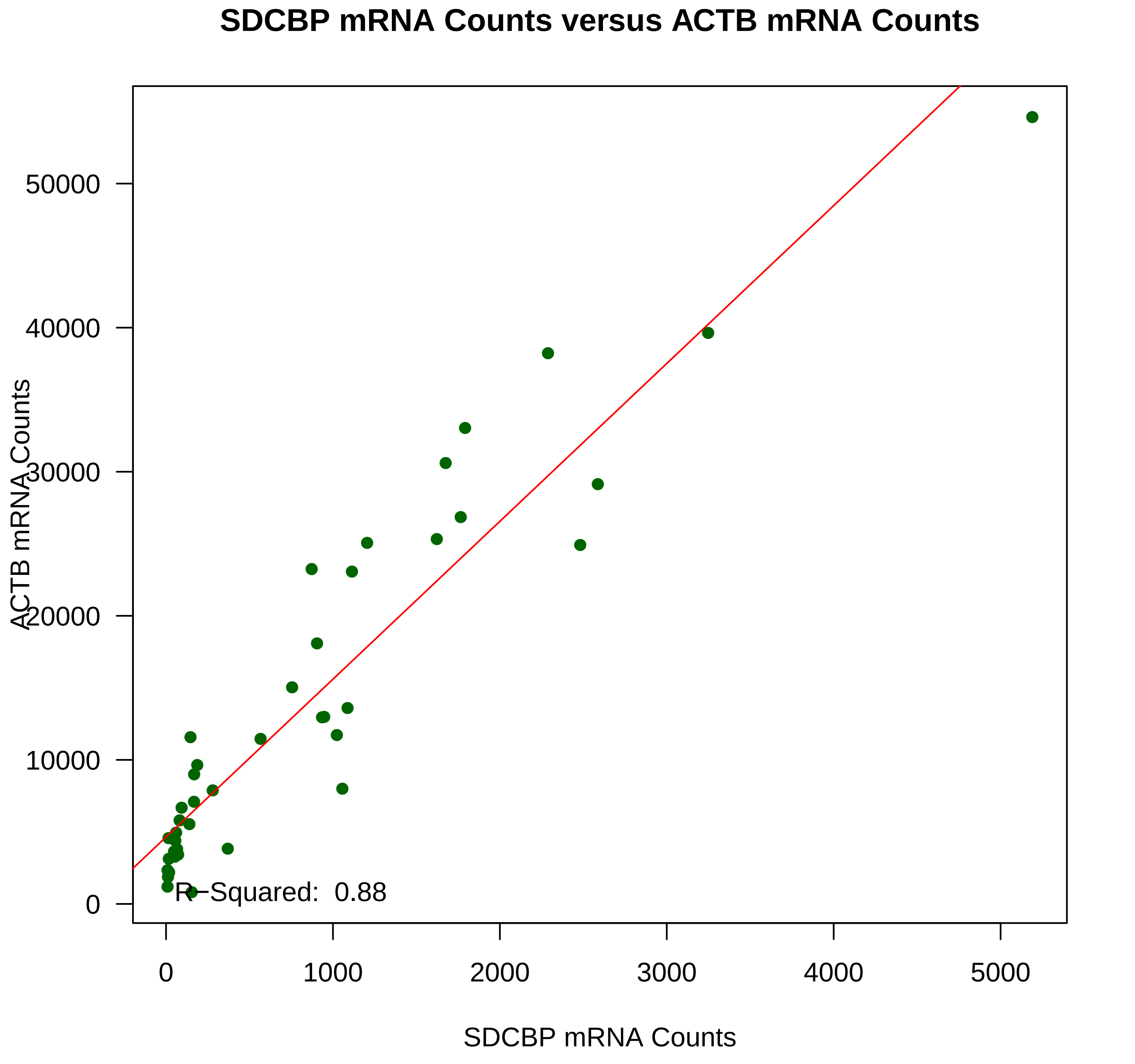
SDCBP mRNA Counts versus ACTB mRNA Counts
ACTB mRNA Counts
50000
40000
30000
20000
10000
0
R−Squared: 0.88
0
1000
2000
3000
4000
5000
SDCBP mRNA Counts

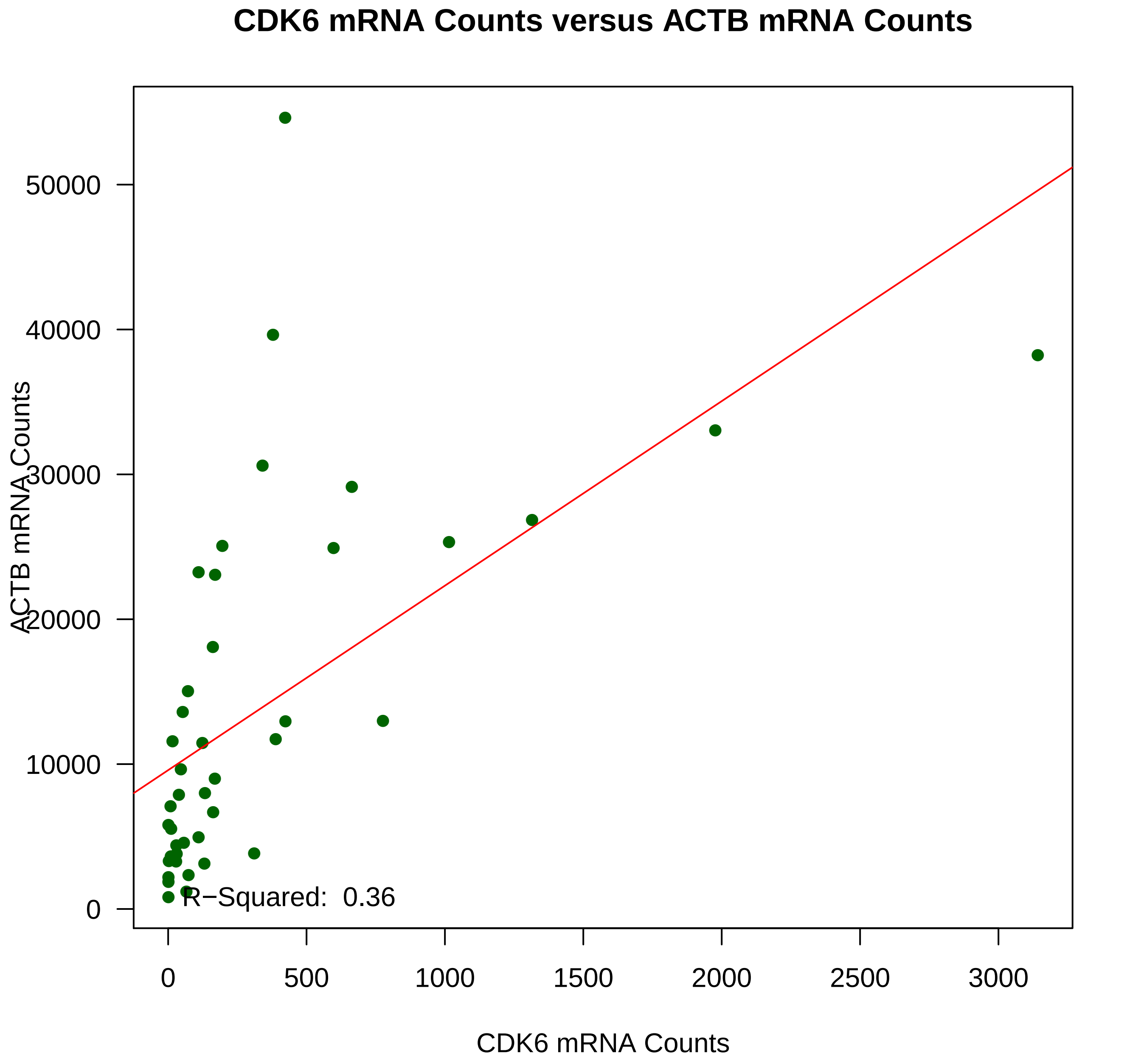
CDK6 mRNA Counts versus ACTB mRNA Counts
ACTB mRNA Counts
50000
40000
30000
20000
10000
0
0
500
1000
1500
2000
2500
3000
CDK6 mRNA Counts
R−Squared: 0.36

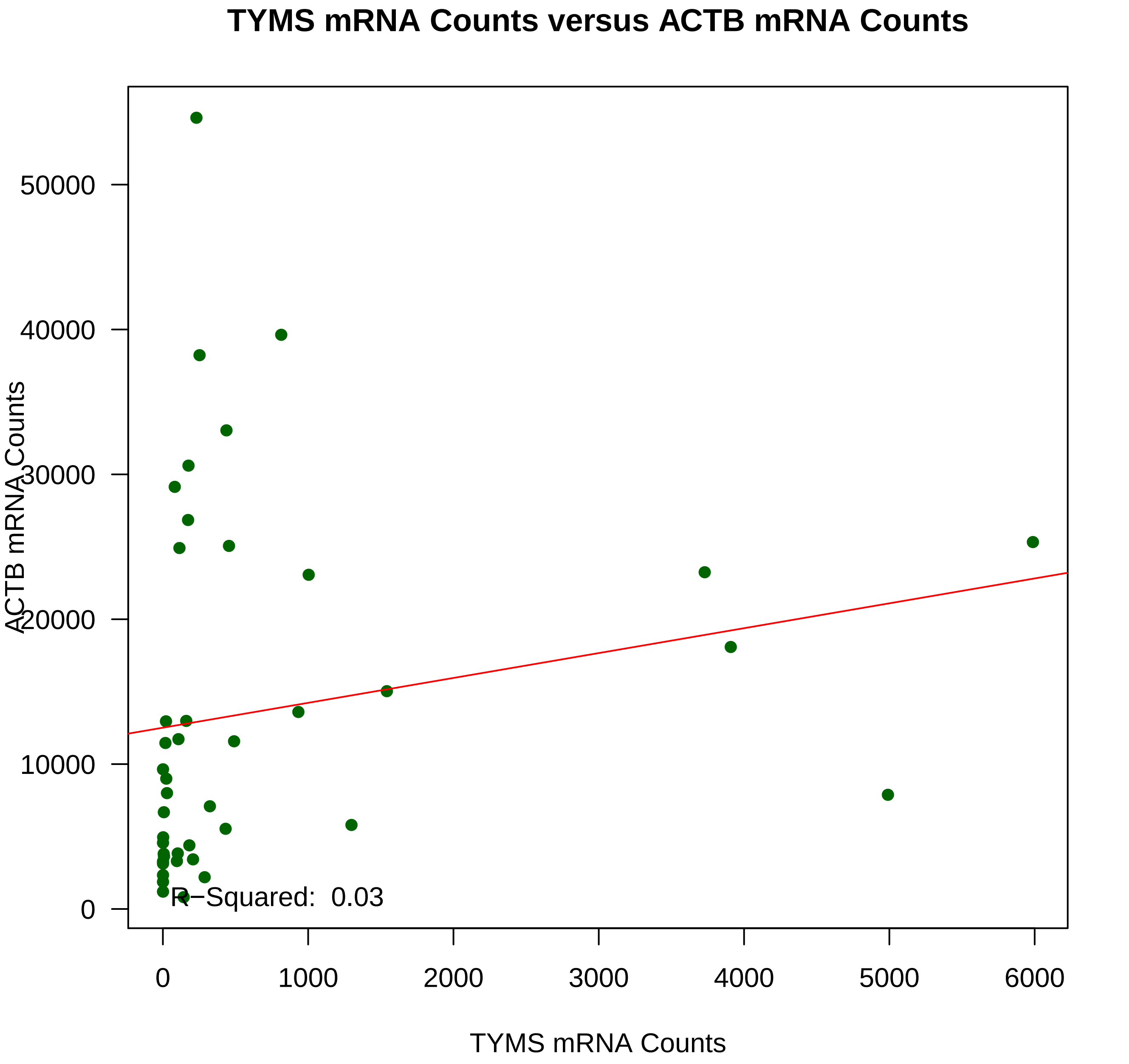

TYMS mRNA Counts versus ACTB mRNA Counts
ACTB mRNA Counts
TYMS mRNA Counts
0
10000
20000
30000
40000
50000
0
1000
2000
3000
4000
5000
6000
R−Squared: 0.03

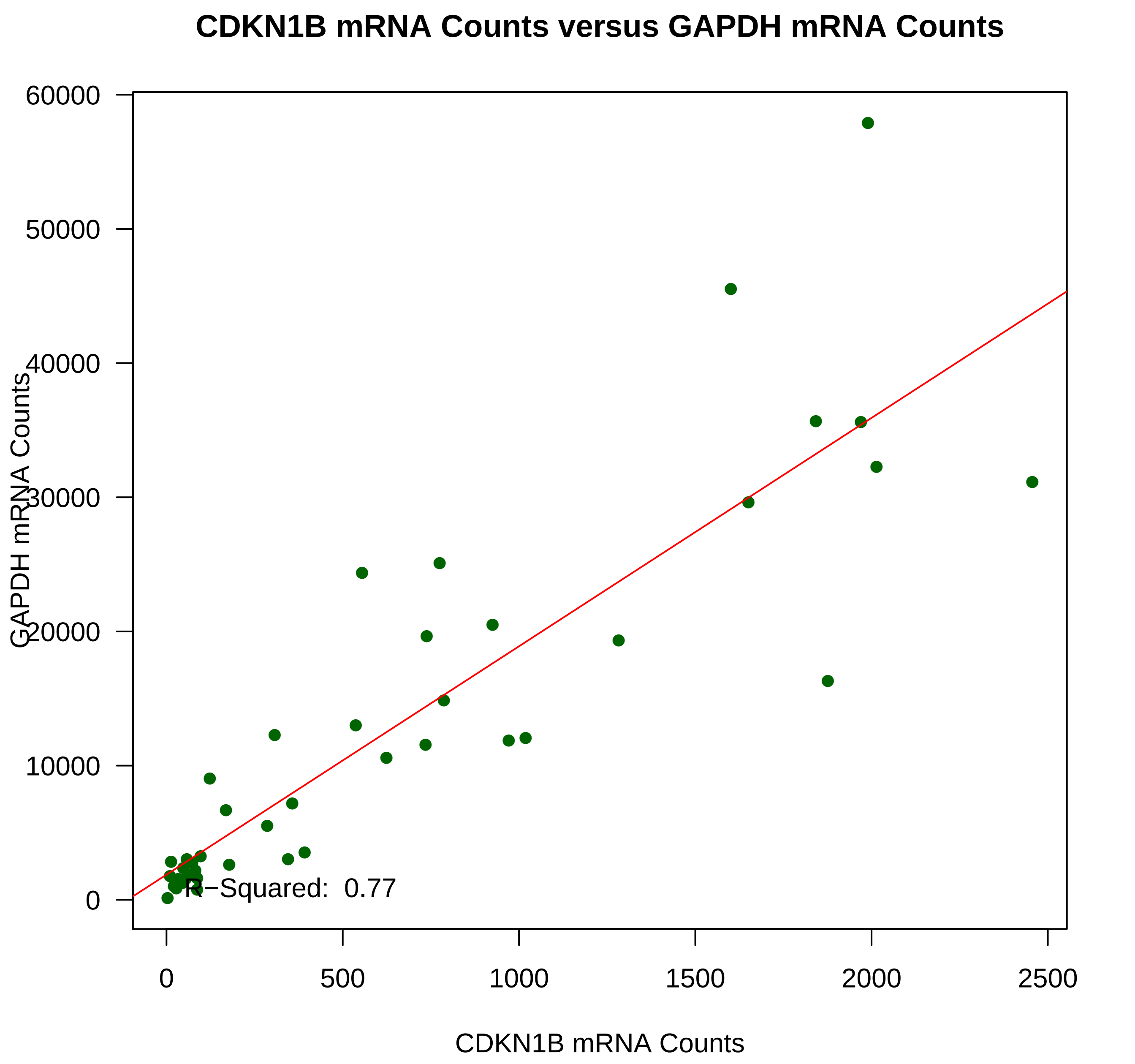
CDKN1B mRNA Counts versus GAPDH mRNA Counts
60000
50000
40000
30000
20000
10000
0
R−Squared: 0.77
0
500
1000
1500
2000
2500
CDKN1B mRNA Counts
GAPDH mRNA Counts

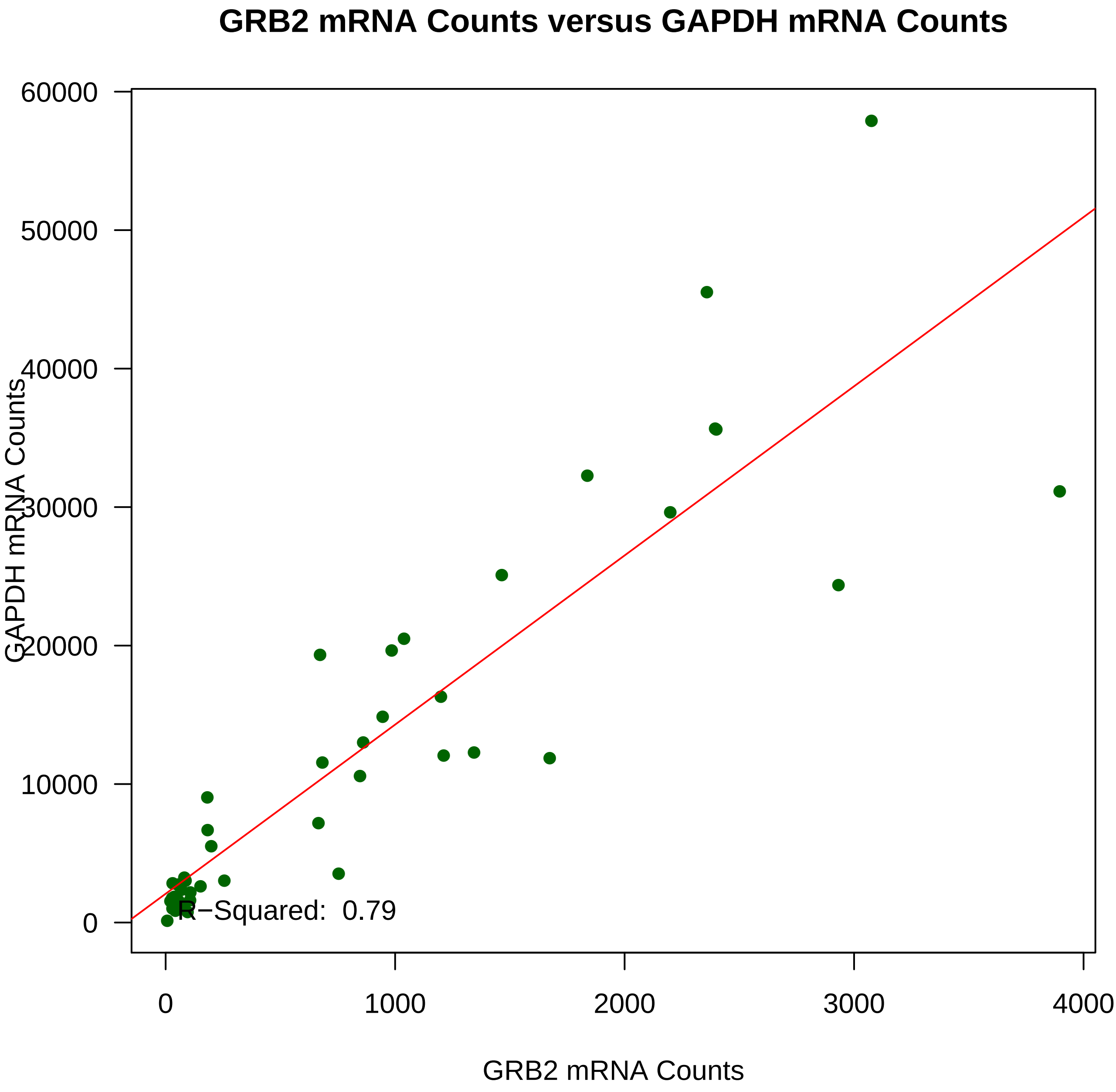
GRB2 mRNA Counts versus GAPDH mRNA Counts
GAPDH mRNA Counts
60000
50000
40000
30000
20000
10000
0
R−Squared: 0.79
0
1000
2000
3000
4000
GRB2 mRNA Counts

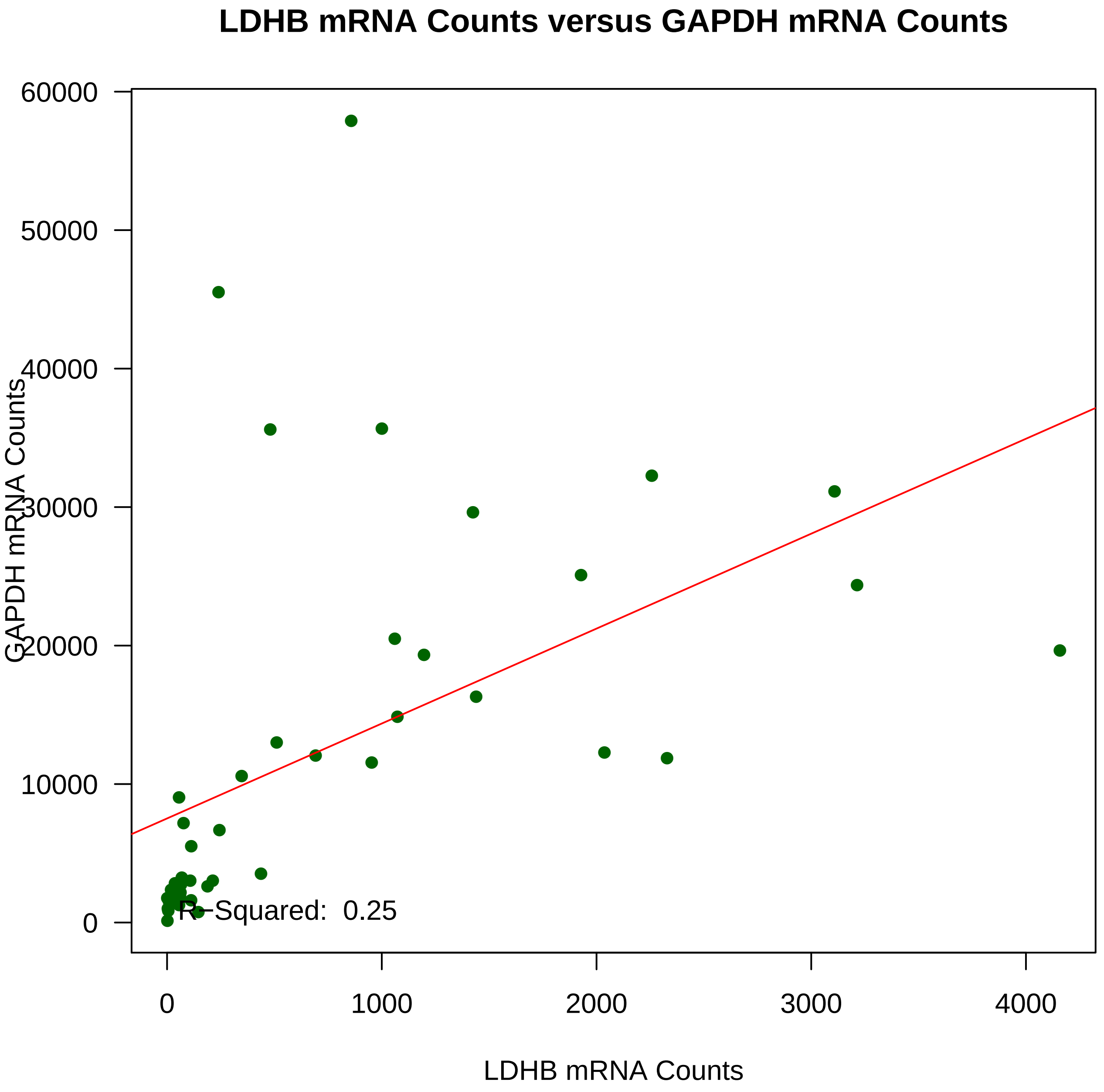
LDHB mRNA Counts versus GAPDH mRNA Counts
60000
50000
40000
30000
20000
10000
0
GAPDH mRNA Counts
R−Squared: 0.25
0
1000
2000
3000
4000
LDHB mRNA Counts

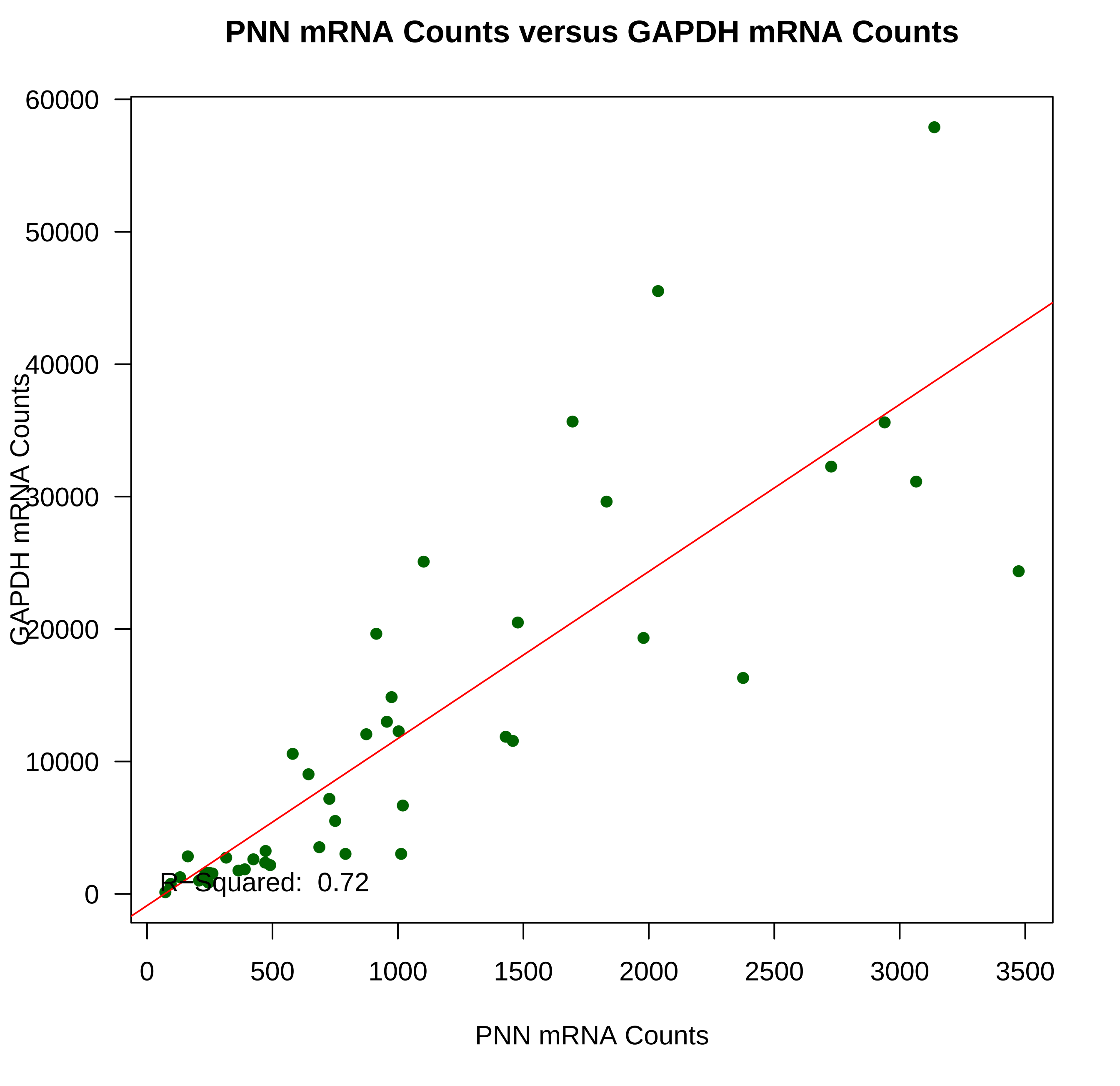
PNN mRNA Counts versus GAPDH mRNA Counts
GAPDH mRNA Counts
PNN mRNA Counts
0
10000
20000
30000
40000
50000
60000
0
500
1000
1500
2000
2500
3000
3500
R−Squared: 0.72

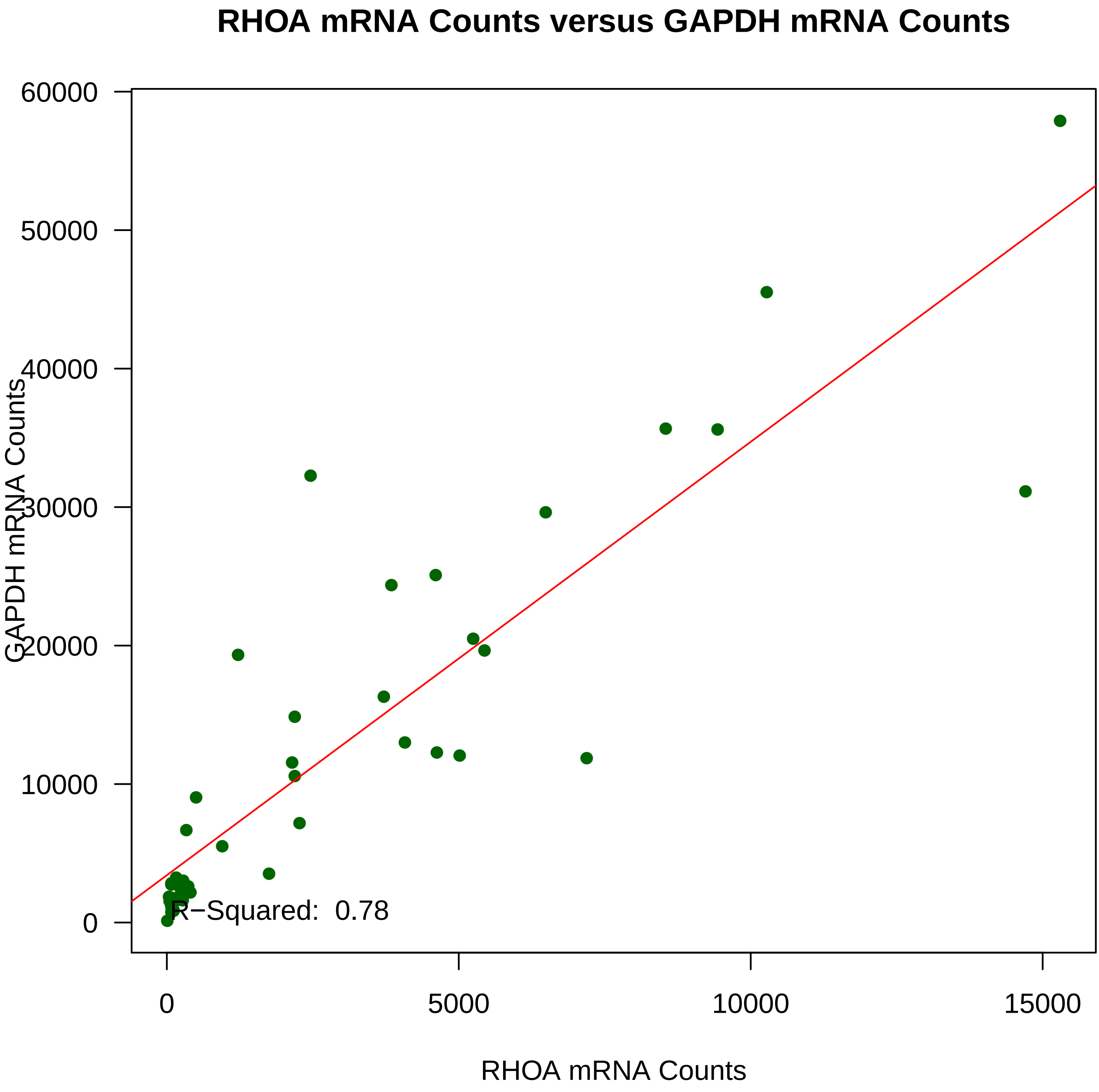
RHOA mRNA Counts versus GAPDH mRNA Counts
GAPDH mRNA Counts
60000
50000
40000
30000
20000
10000
0
R−Squared: 0.78
0
5000
10000
15000
RHOA mRNA Counts

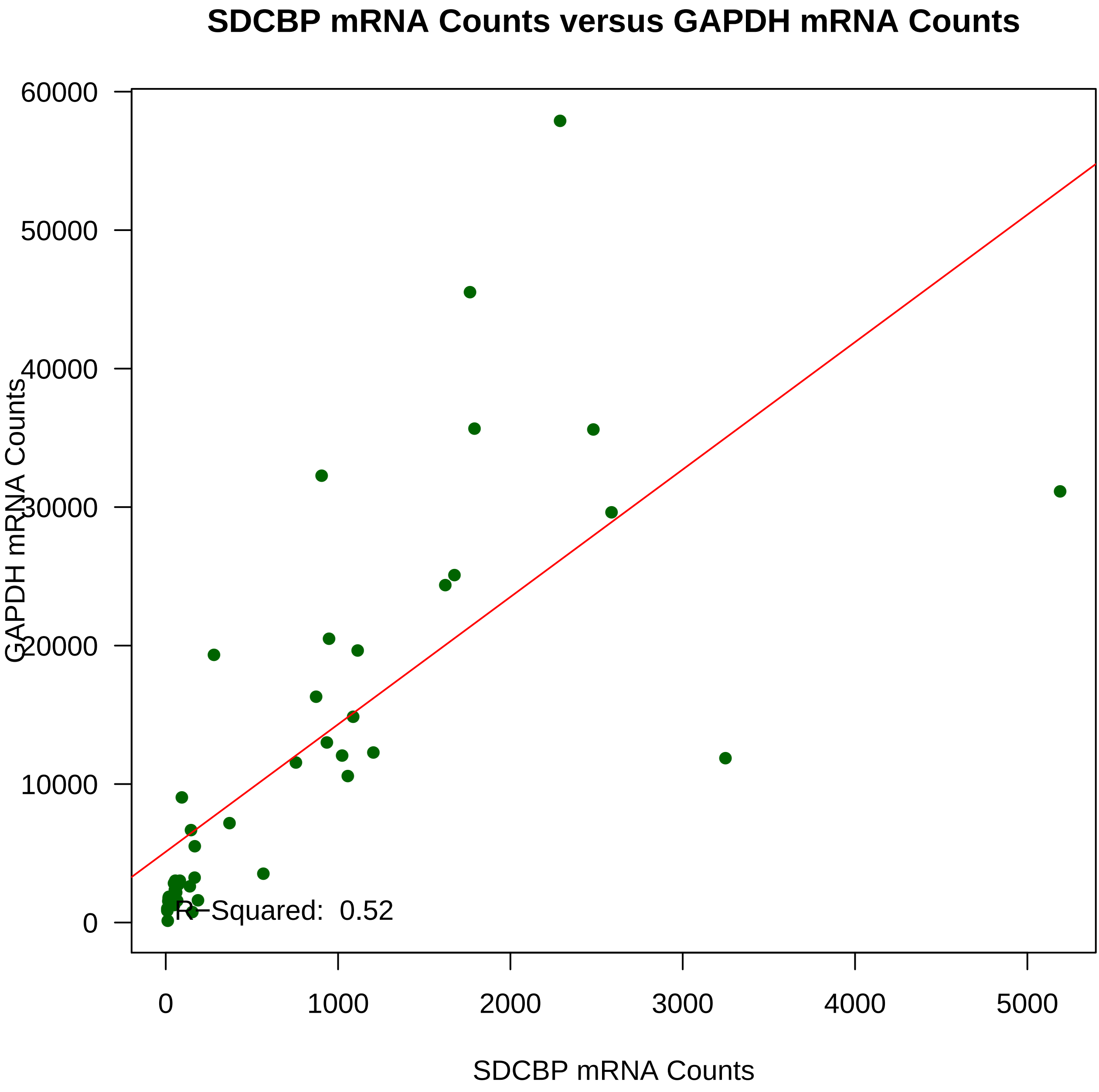
SDCBP mRNA Counts versus GAPDH mRNA Counts
GAPDH mRNA Counts
60000
50000
40000
30000
20000
10000
0
R−Squared: 0.52
0
1000
2000
3000
4000
5000
SDCBP mRNA Counts

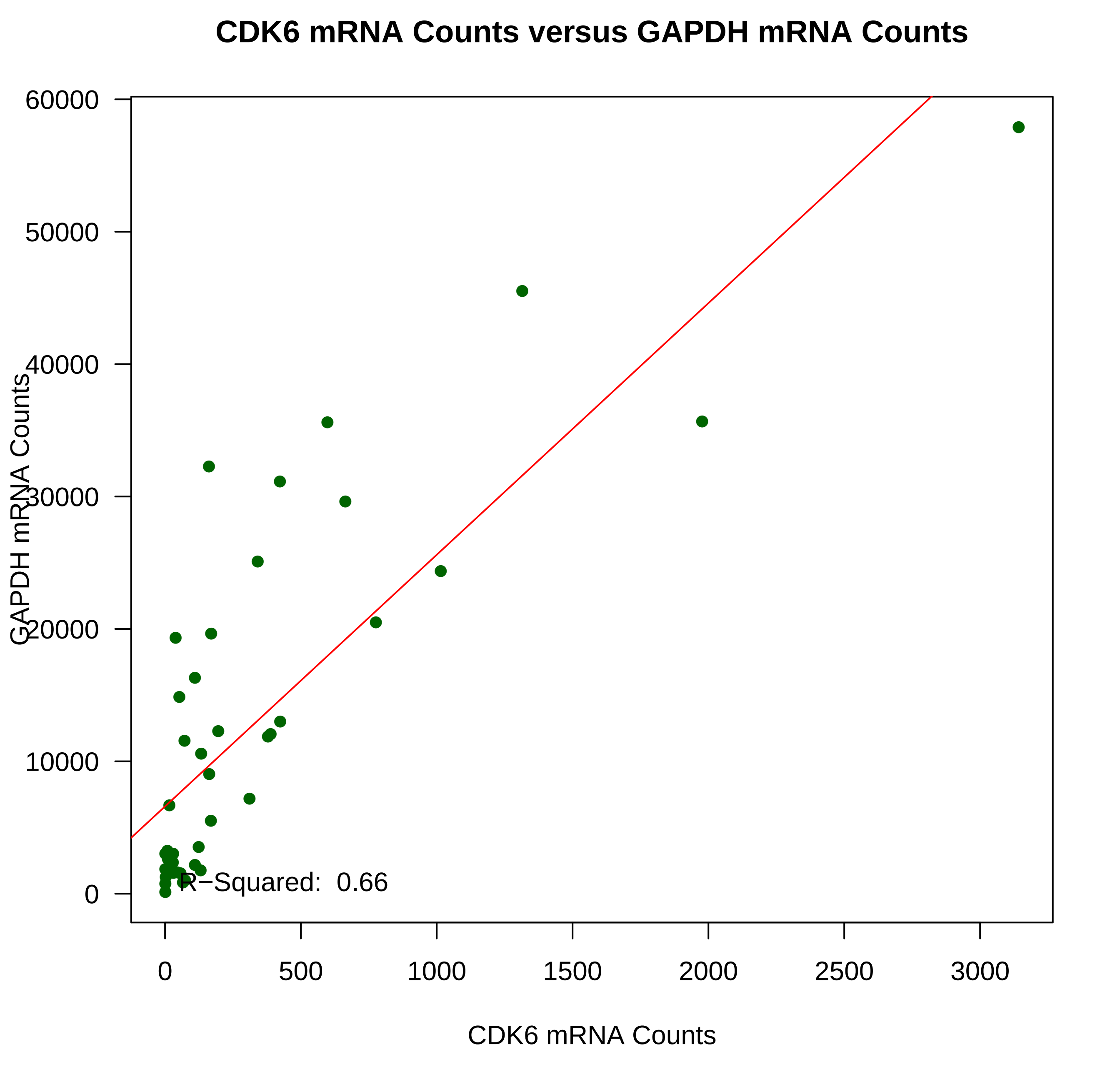

CDK6 mRNA Counts versus GAPDH mRNA Counts
60000
50000
40000
30000
20000
10000
0
GAPDH mRNA Counts
R−Squared: 0.66
0
500
1000
1500
2000
2500
3000
CDK6 mRNA Counts

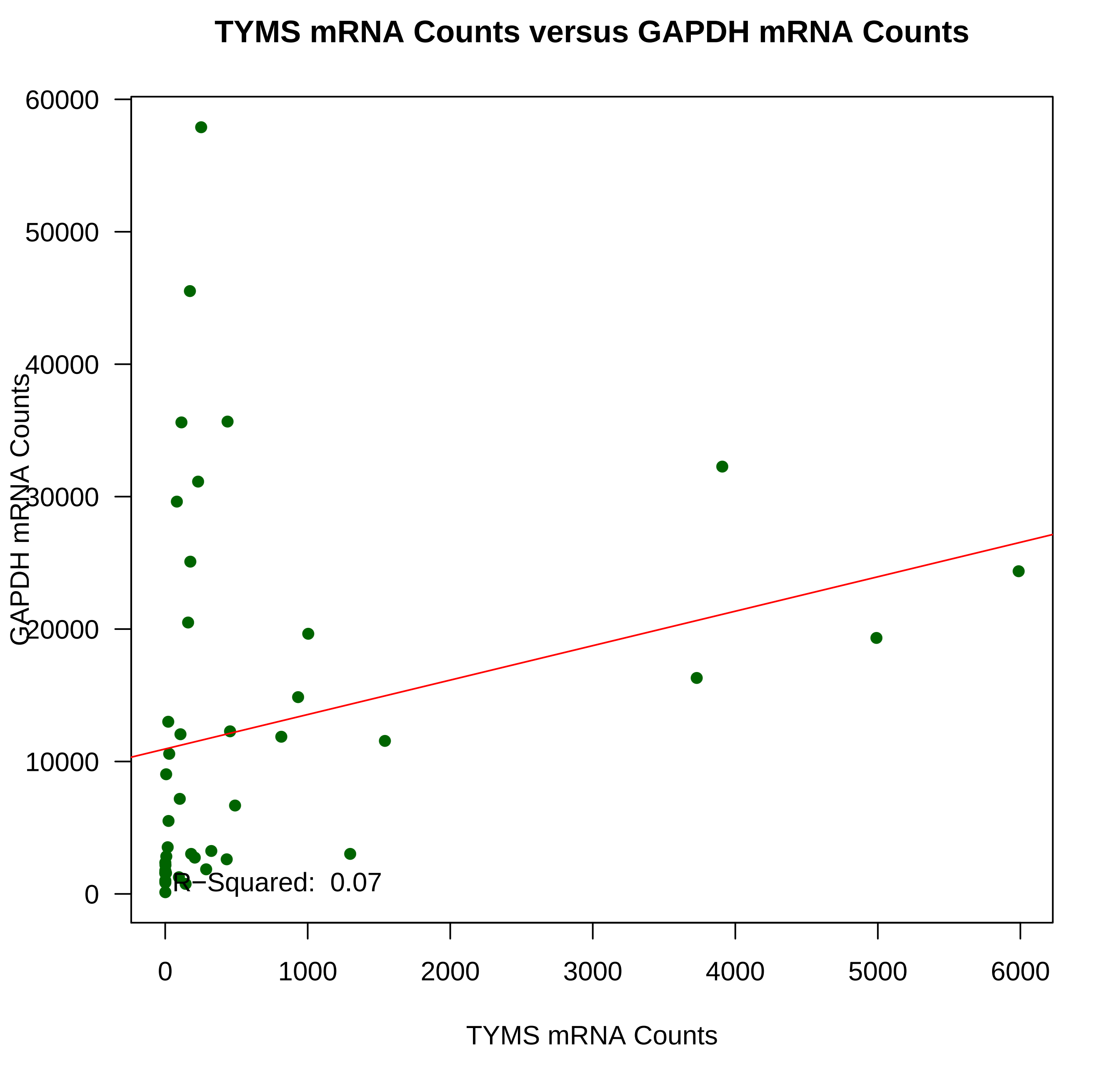

TYMS mRNA Counts versus GAPDH mRNA Counts
60000
50000
40000
30000
20000
10000
0
GAPDH mRNA Counts
R−Squared: 0.07
0
1000
2000
3000
4000
5000
6000
TYMS mRNA Counts

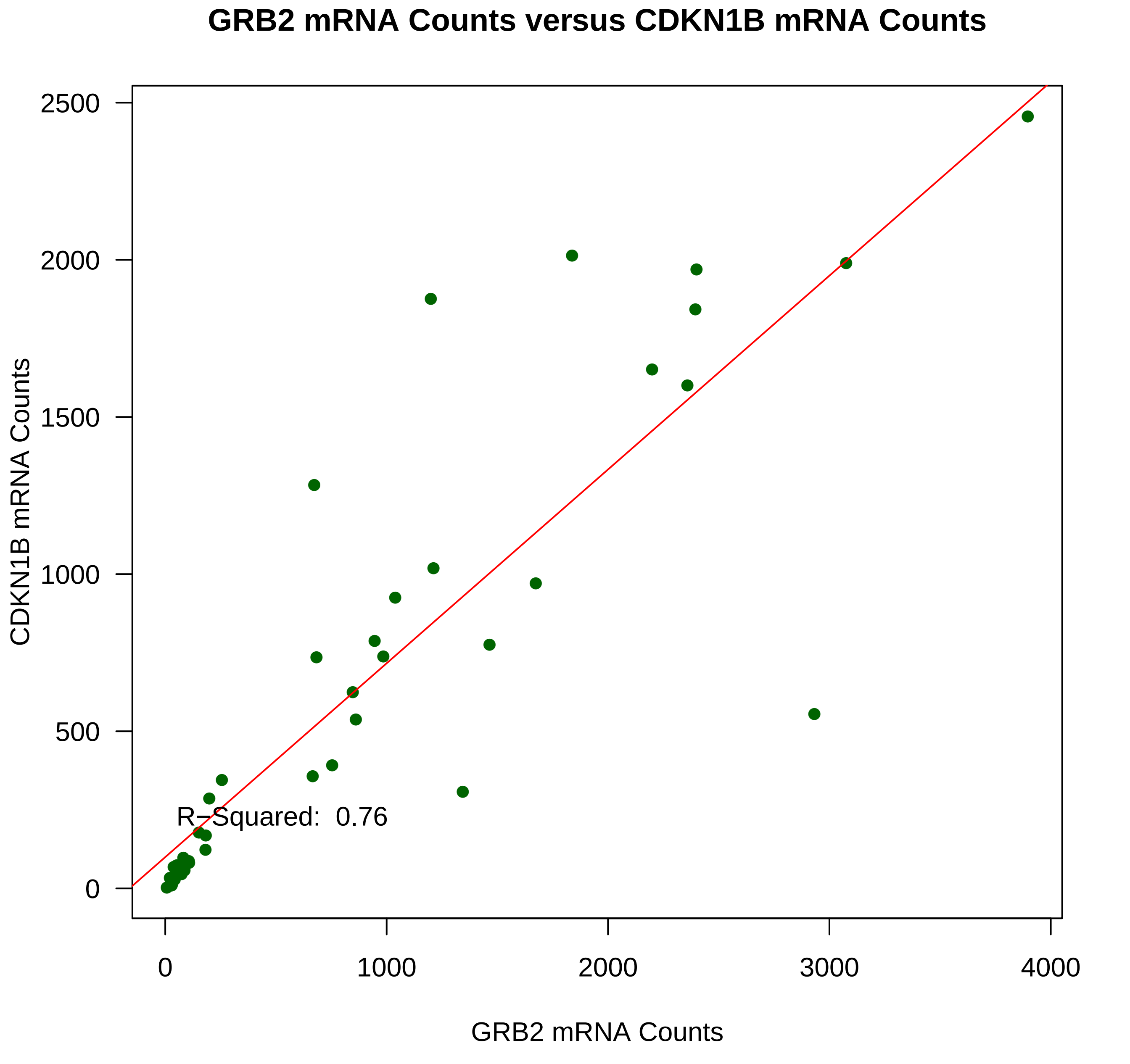
GRB2 mRNA Counts versus CDKN1B mRNA Counts
2500
2000
1500
1000
500
0
CDKN1B mRNA Counts
0
1000
2000
3000
4000
GRB2 mRNA Counts
R−Squared: 0.76

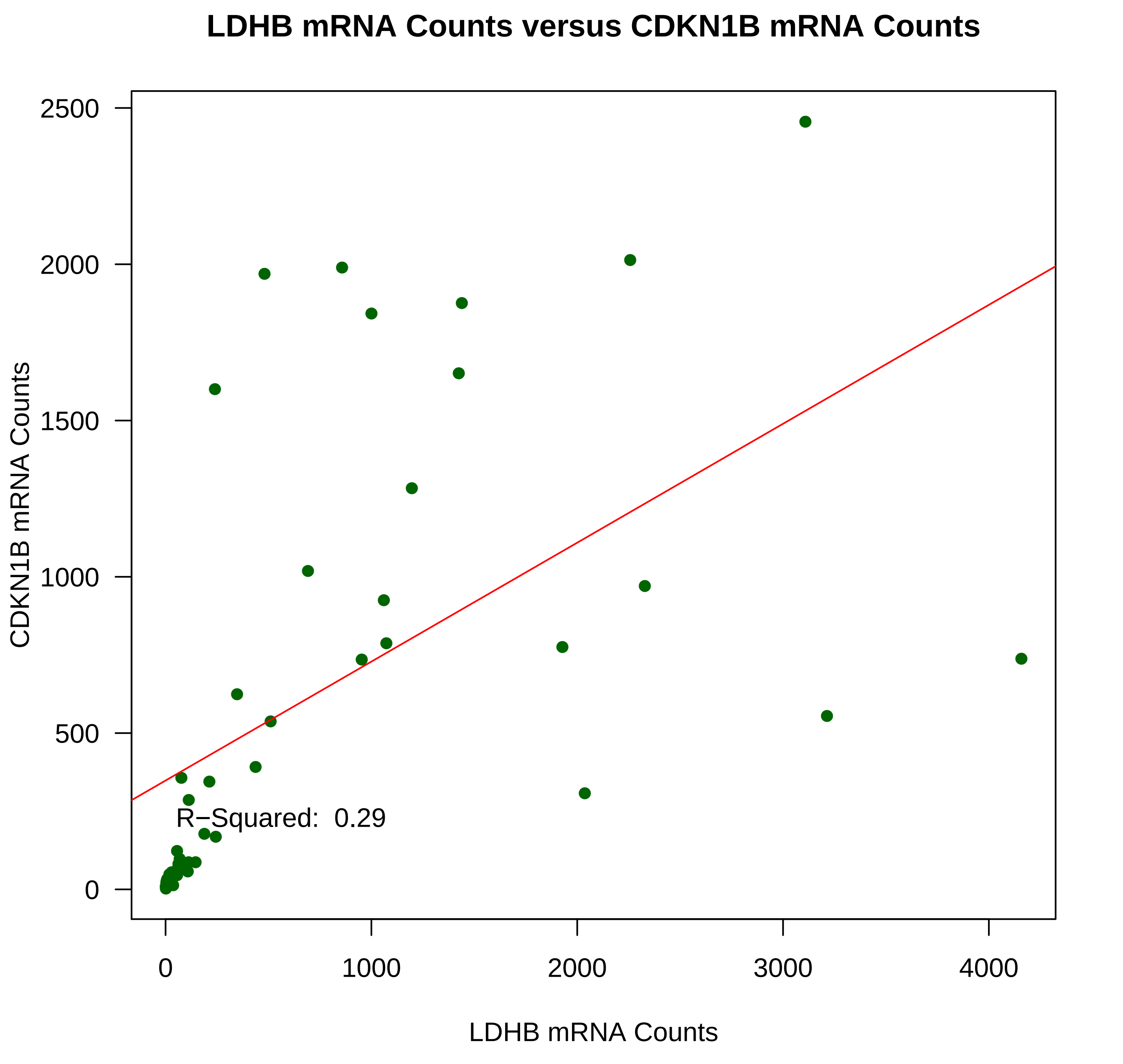
LDHB mRNA Counts versus CDKN1B mRNA Counts
CDKN1B mRNA Counts
LDHB mRNA Counts
0
500
1000
1500
2000
2500
0
1000
2000
3000
4000
R−Squared: 0.29

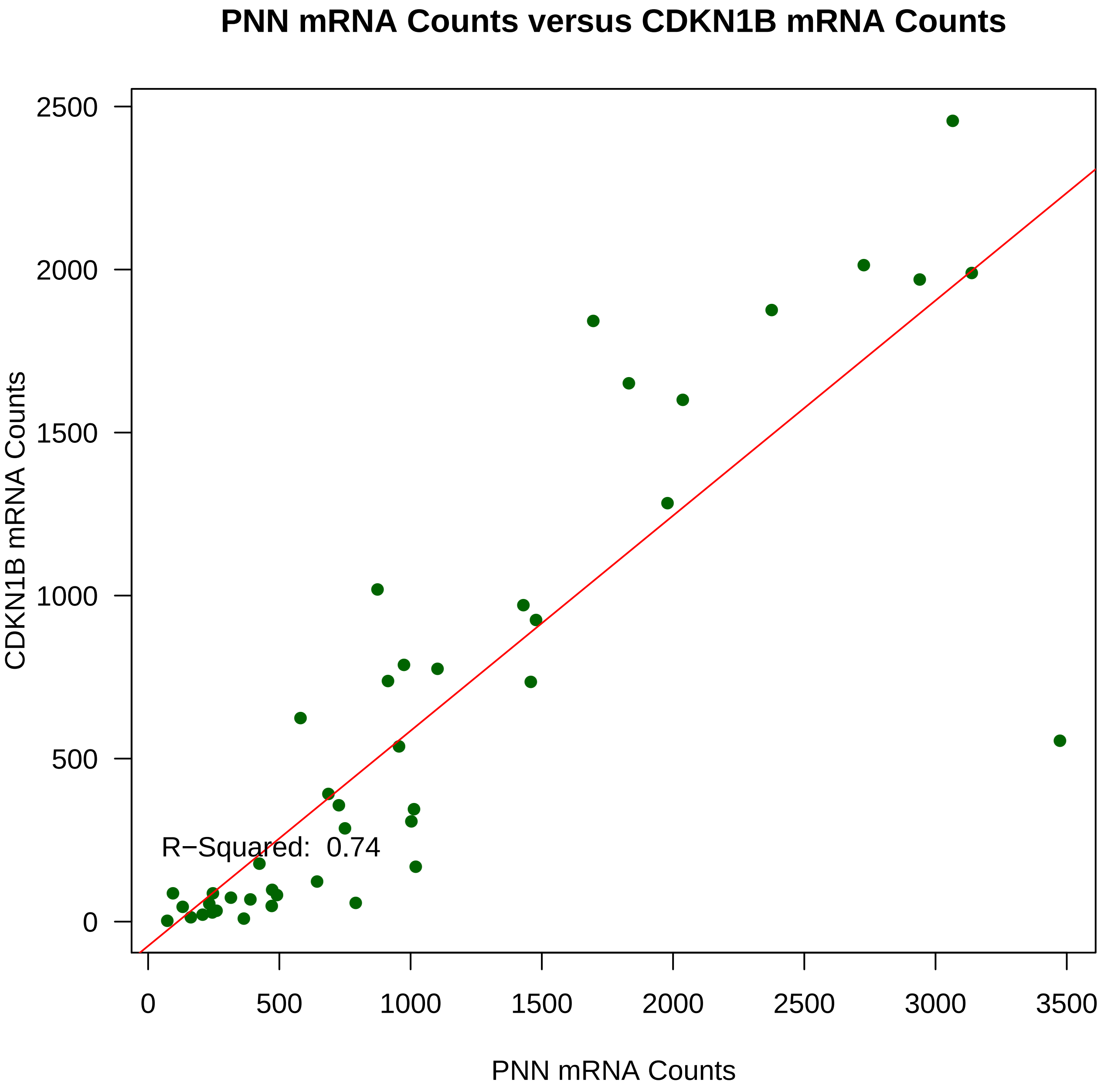

PNN mRNA Counts versus CDKN1B mRNA Counts
CDKN1B mRNA Counts
0
500
1000
1500
2000
2500
R−Squared: 0.74
0
500
1000
1500
2000
2500
3000
3500
PNN mRNA Counts

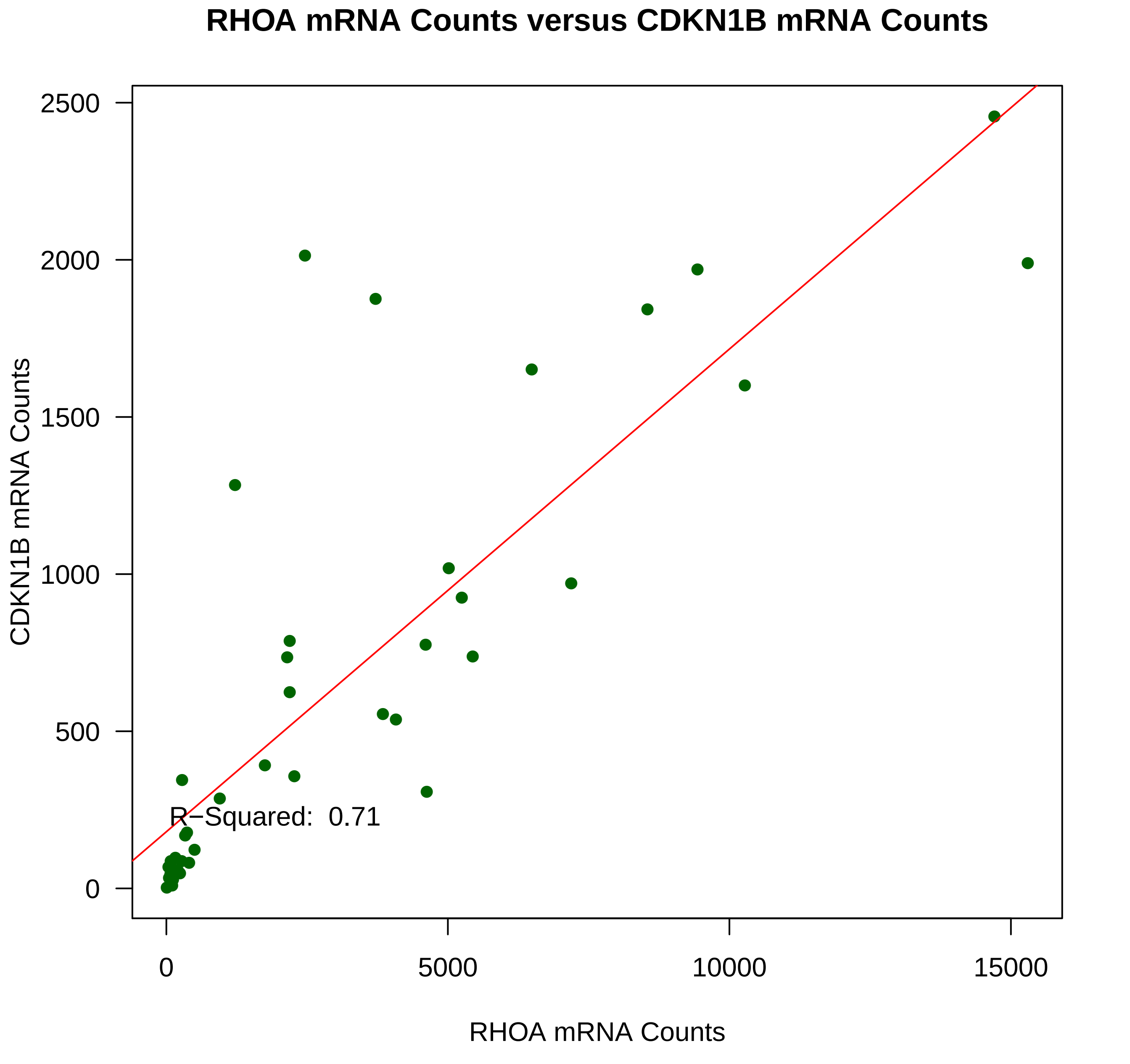

RHOA mRNA Counts versus CDKN1B mRNA Counts
2500
2000
1500
1000
500
0
CDKN1B mRNA Counts
R−Squared: 0.71
0
5000
10000
15000
RHOA mRNA Counts

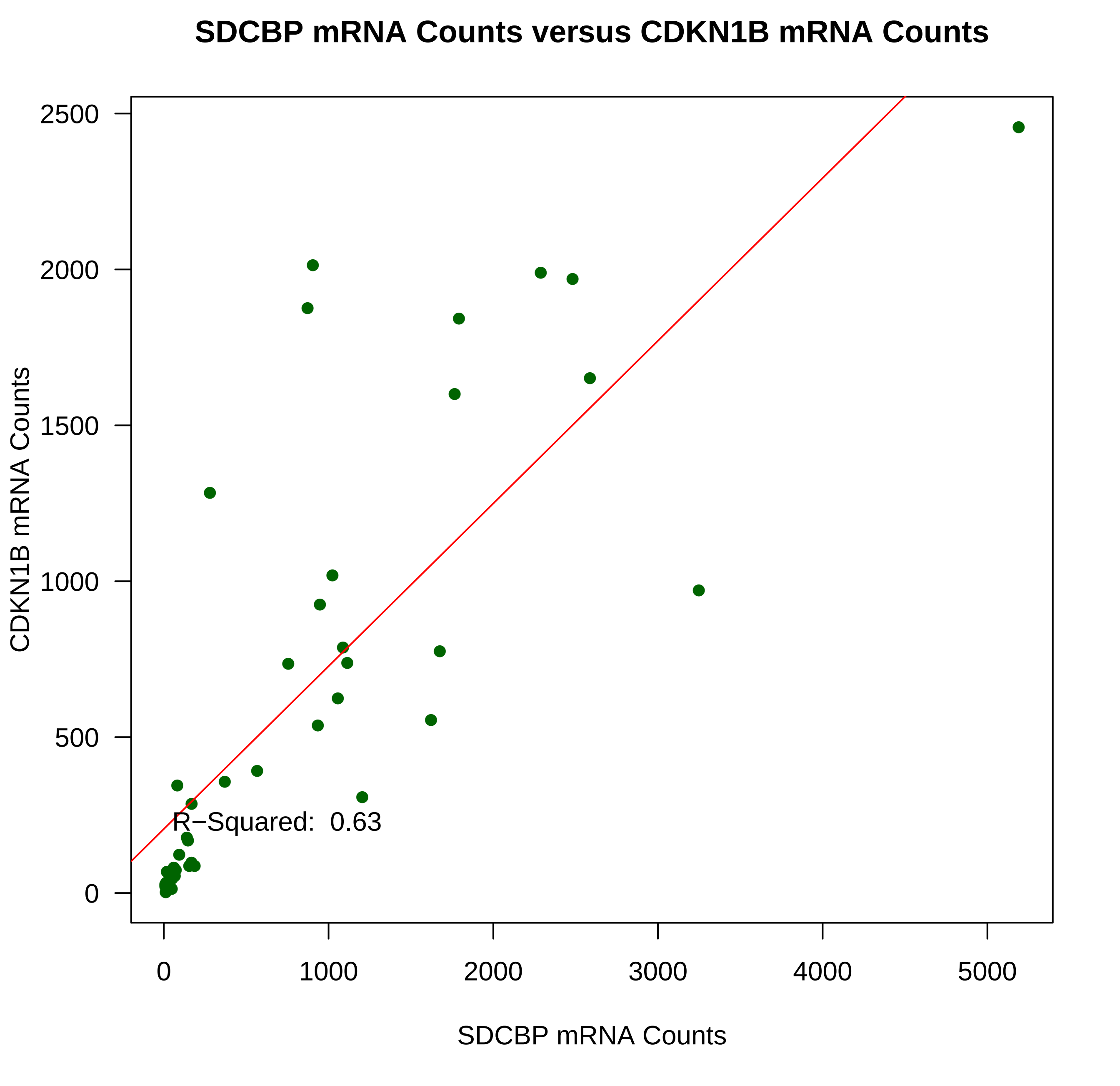
SDCBP mRNA Counts versus CDKN1B mRNA Counts
CDKN1B mRNA Counts
SDCBP mRNA Counts
R−Squared: 0.63
0
500
1000
1500
2000
2500
0
1000
2000
3000
4000
5000

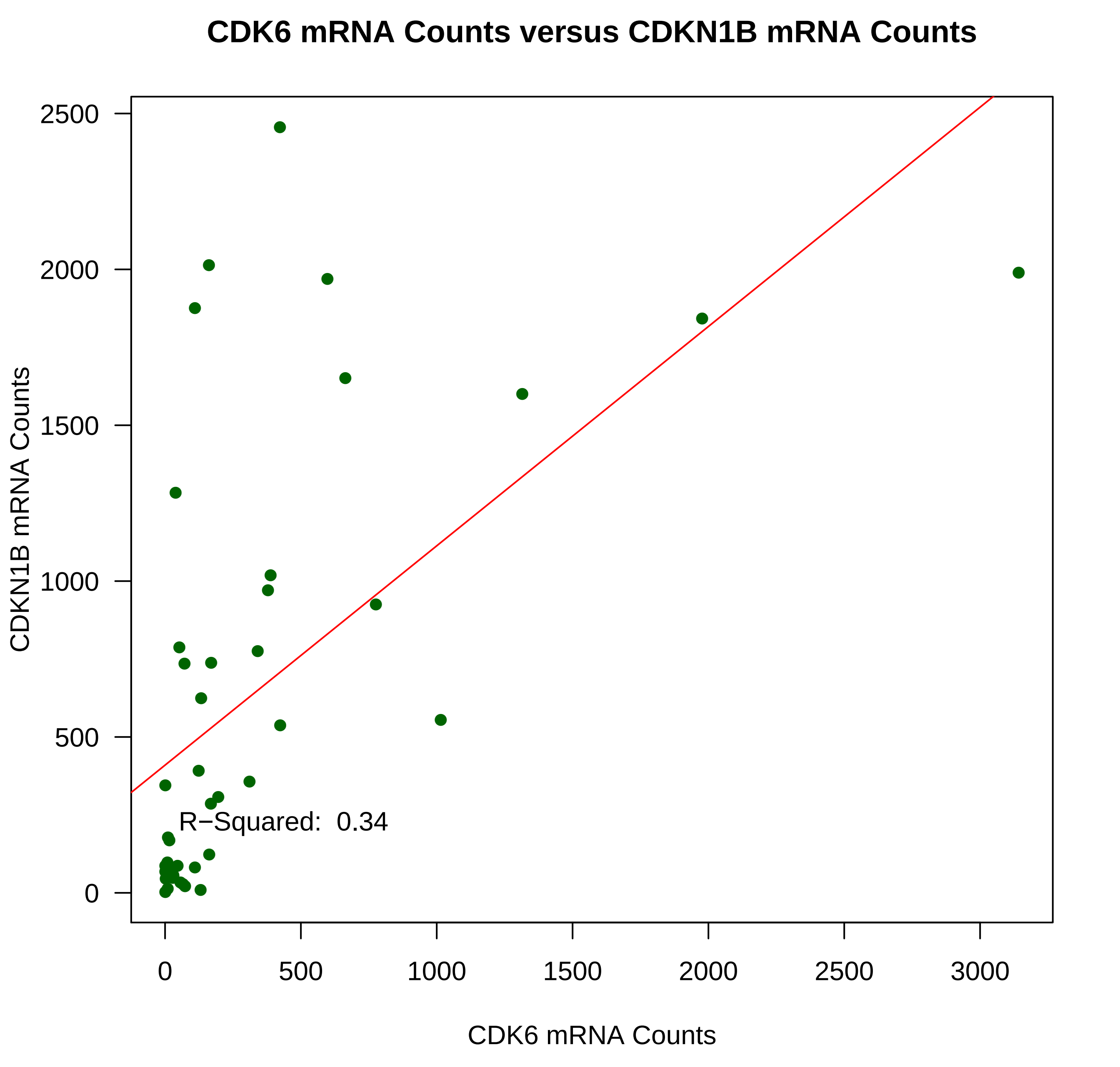
CDK6 mRNA Counts versus CDKN1B mRNA Counts
CDKN1B mRNA Counts
CDK6 mRNA Counts
R−Squared: 0.34
0
500
1000
1500
2000
2500
3000

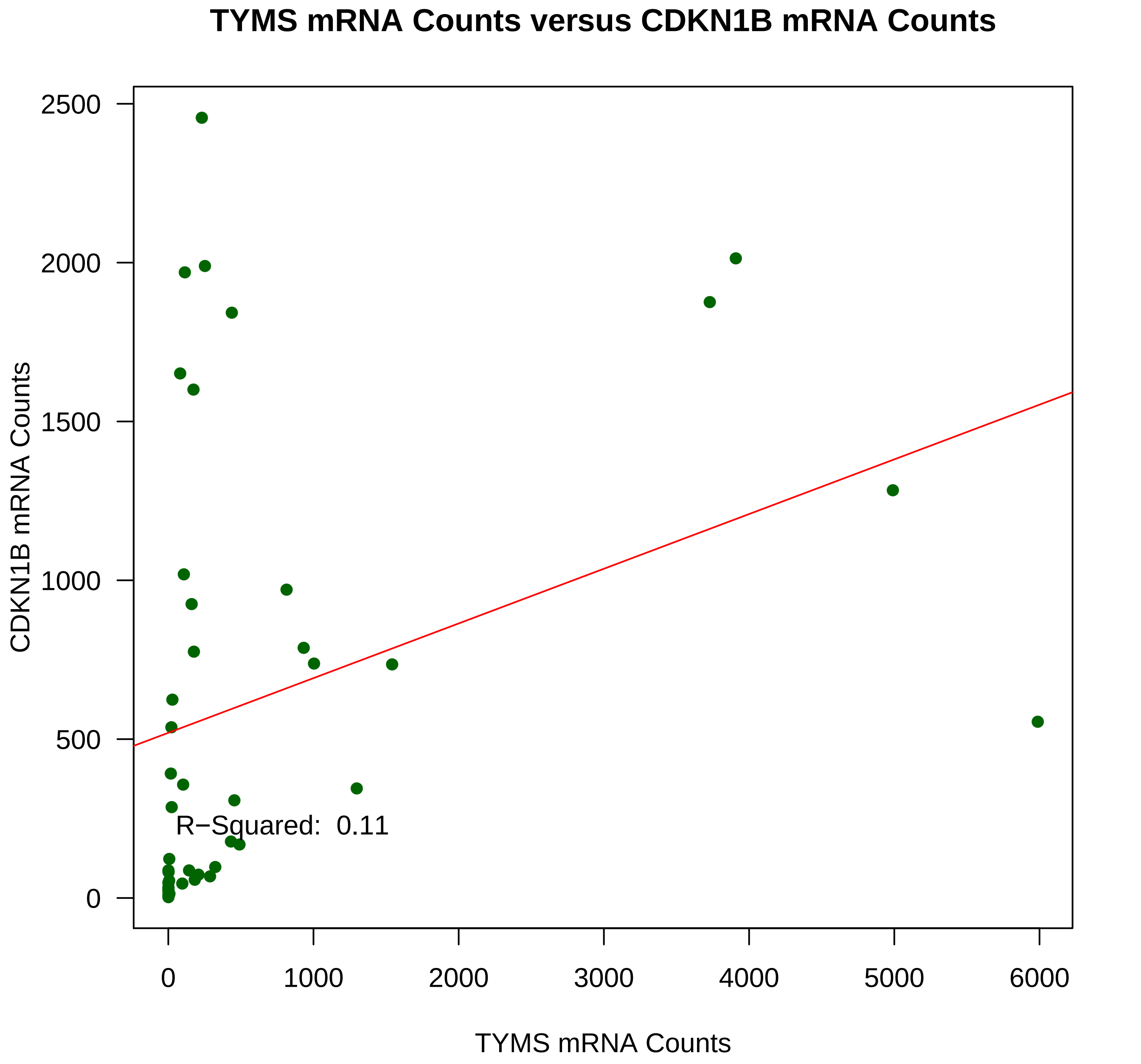

TYMS mRNA Counts versus CDKN1B mRNA Counts
CDKN1B mRNA Counts
0
500
1000
1500
2000
2500
TYMS mRNA Counts
0
1000
2000
3000
4000
5000
6000
R−Squared: 0.11

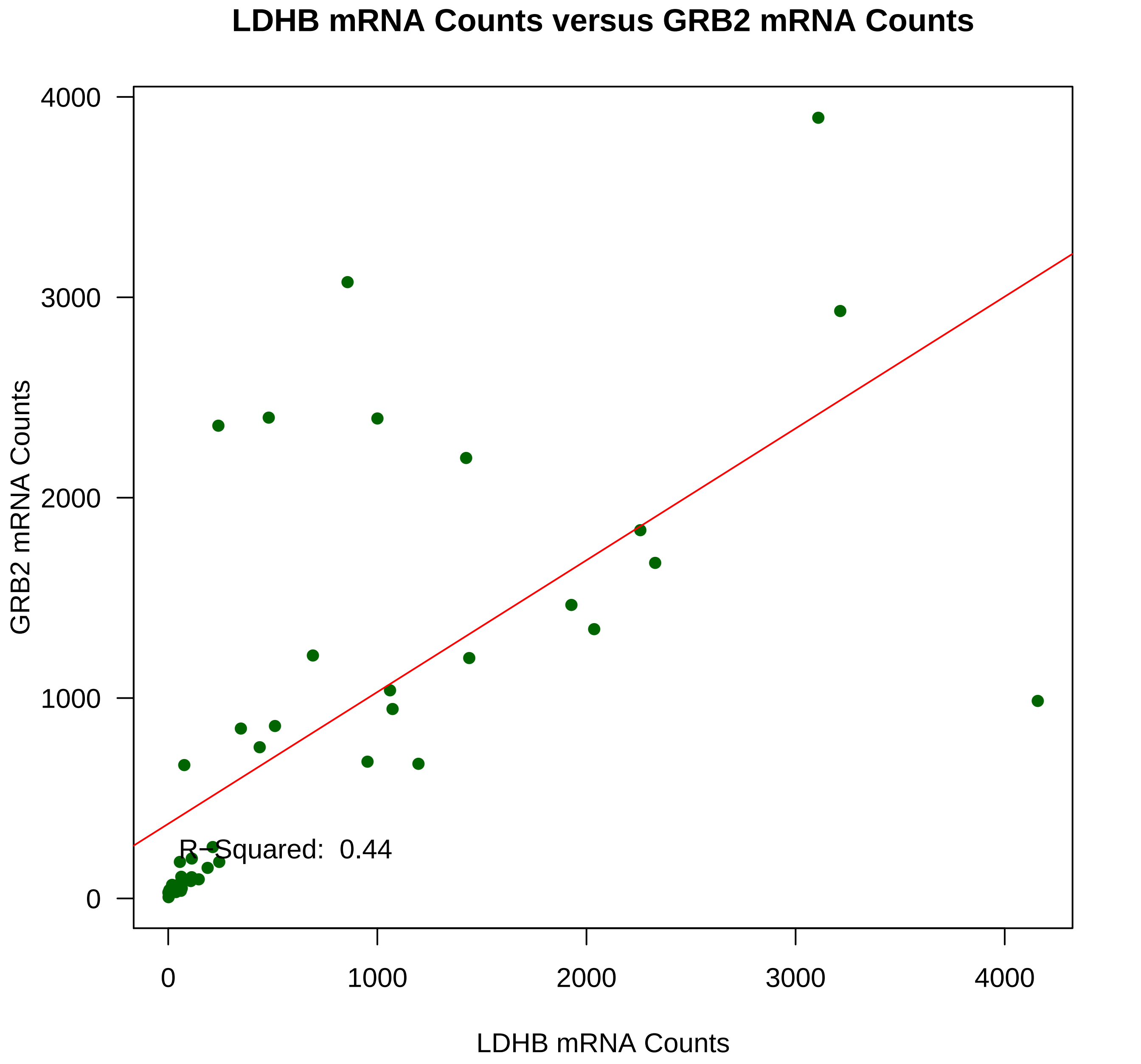
LDHB mRNA Counts versus GRB2 mRNA Counts
4000
3000
2000
1000
0
GRB2 mRNA Counts
R−Squared: 0.44
0
1000
2000
3000
4000
LDHB mRNA Counts

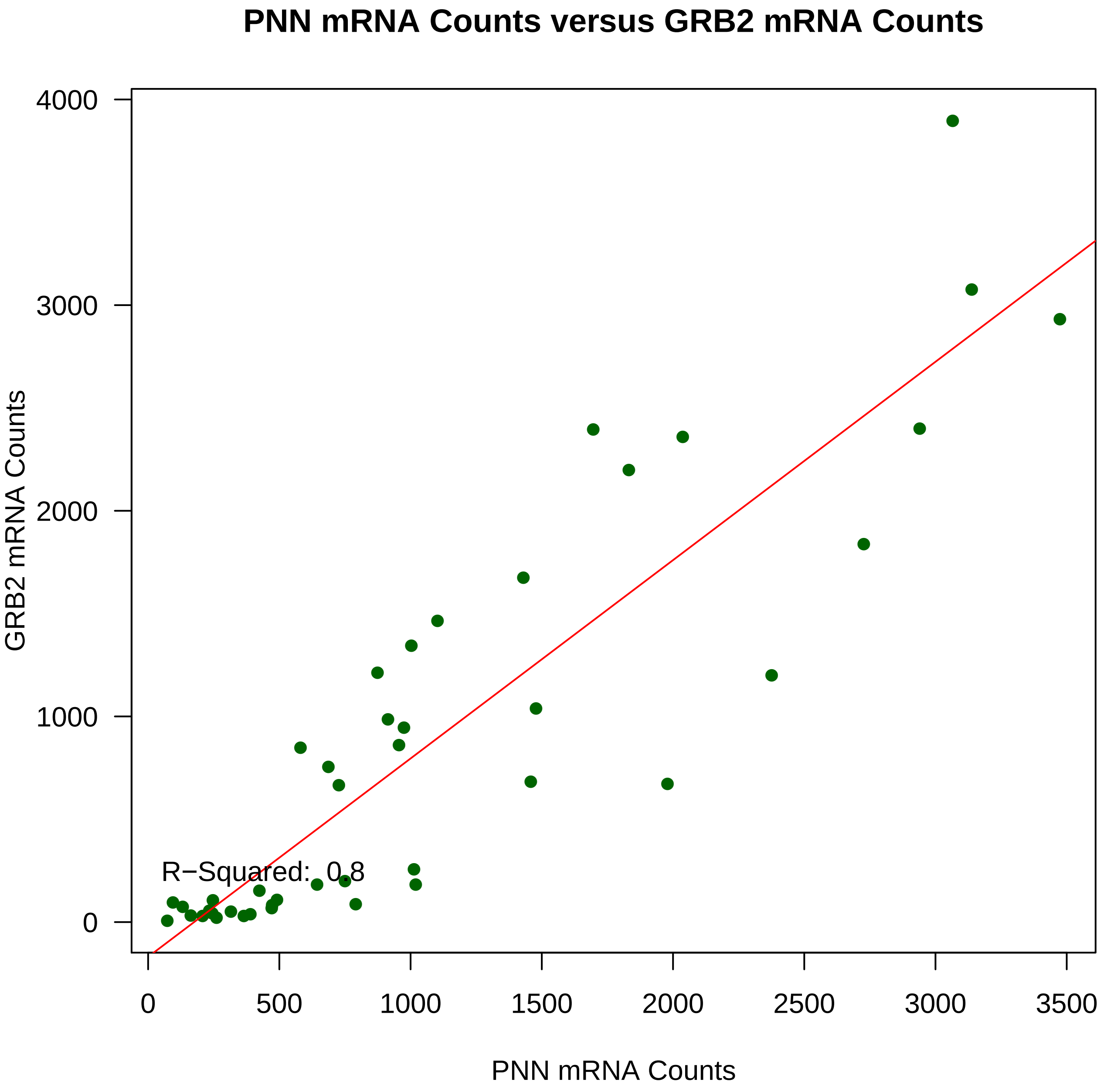
PNN mRNA Counts versus GRB2 mRNA Counts
GRB2 mRNA Counts
4000
3000
2000
1000
0
R−Squared: 0.8
0
500
1000
1500
2000
2500
3000
3500
PNN mRNA Counts

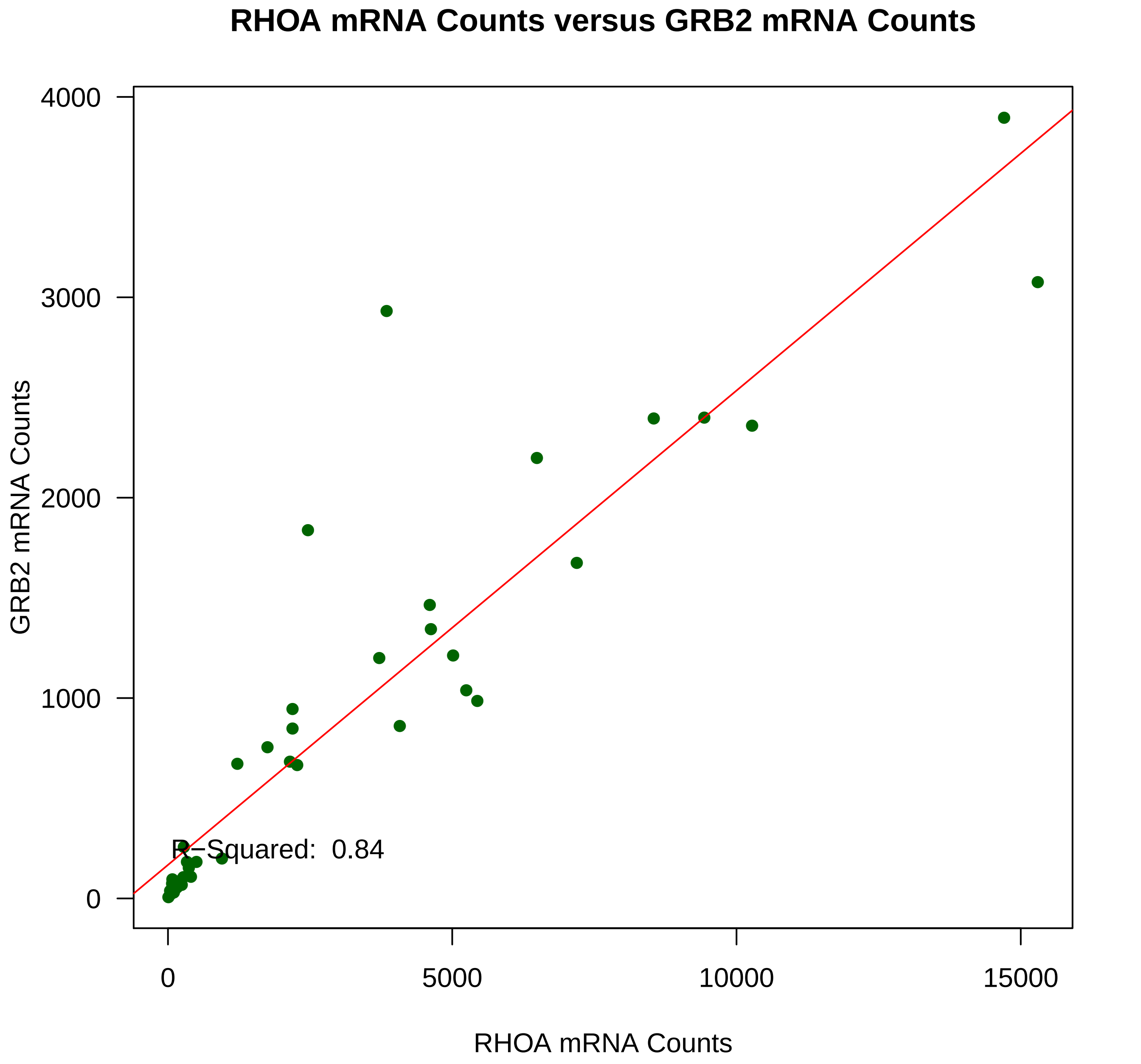

RHOA mRNA Counts versus GRB2 mRNA Counts
GRB2 mRNA Counts
RHOA mRNA Counts
R−Squared: 0.84
0
1000
2000
3000
4000
0
5000
10000
15000

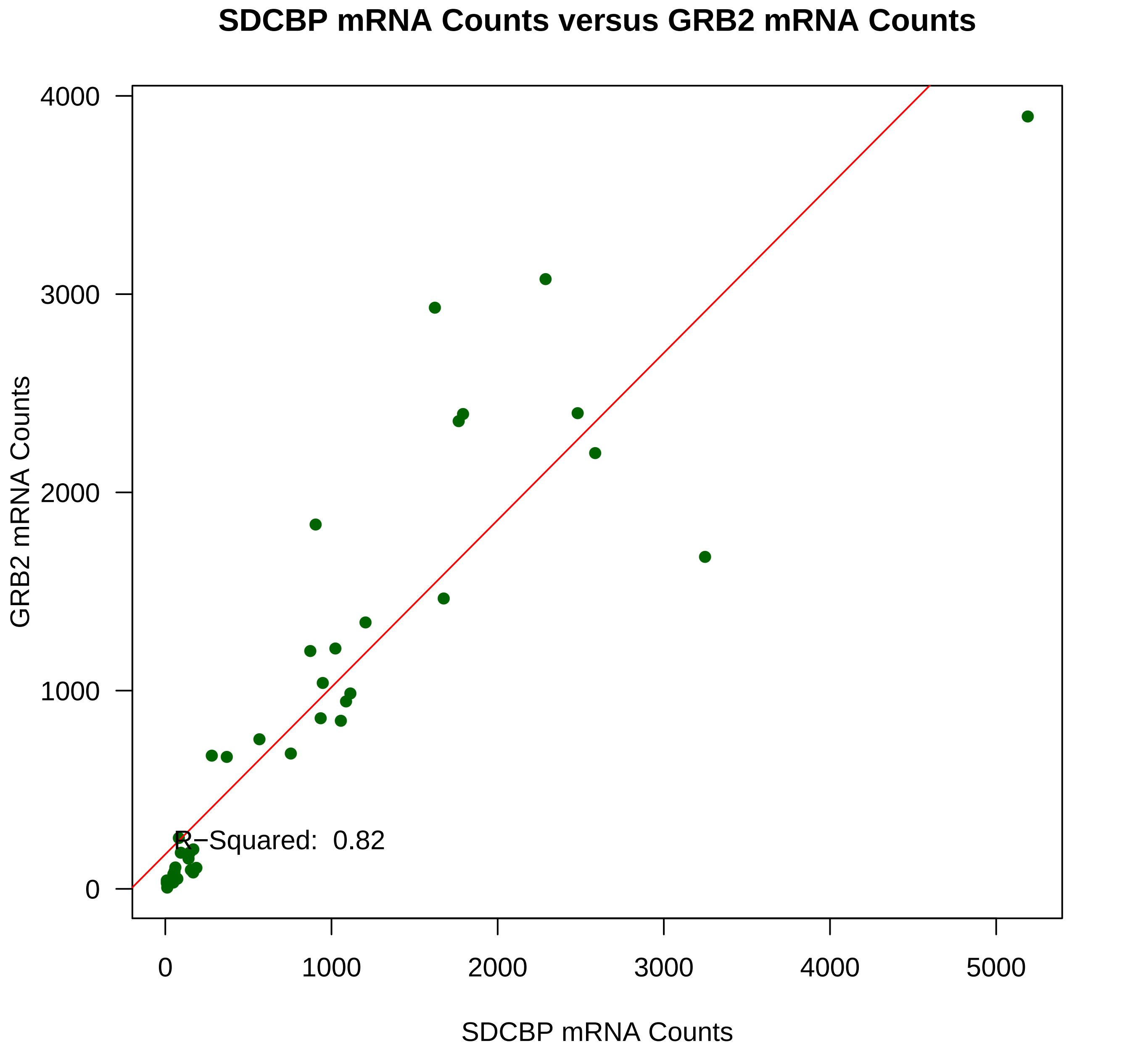

SDCBP mRNA Counts versus GRB2 mRNA Counts
R−Squared: 0.82
GRB2 mRNA Counts
0
1000
2000
3000
4000
SDCBP mRNA Counts
0
1000
2000
3000
4000
5000

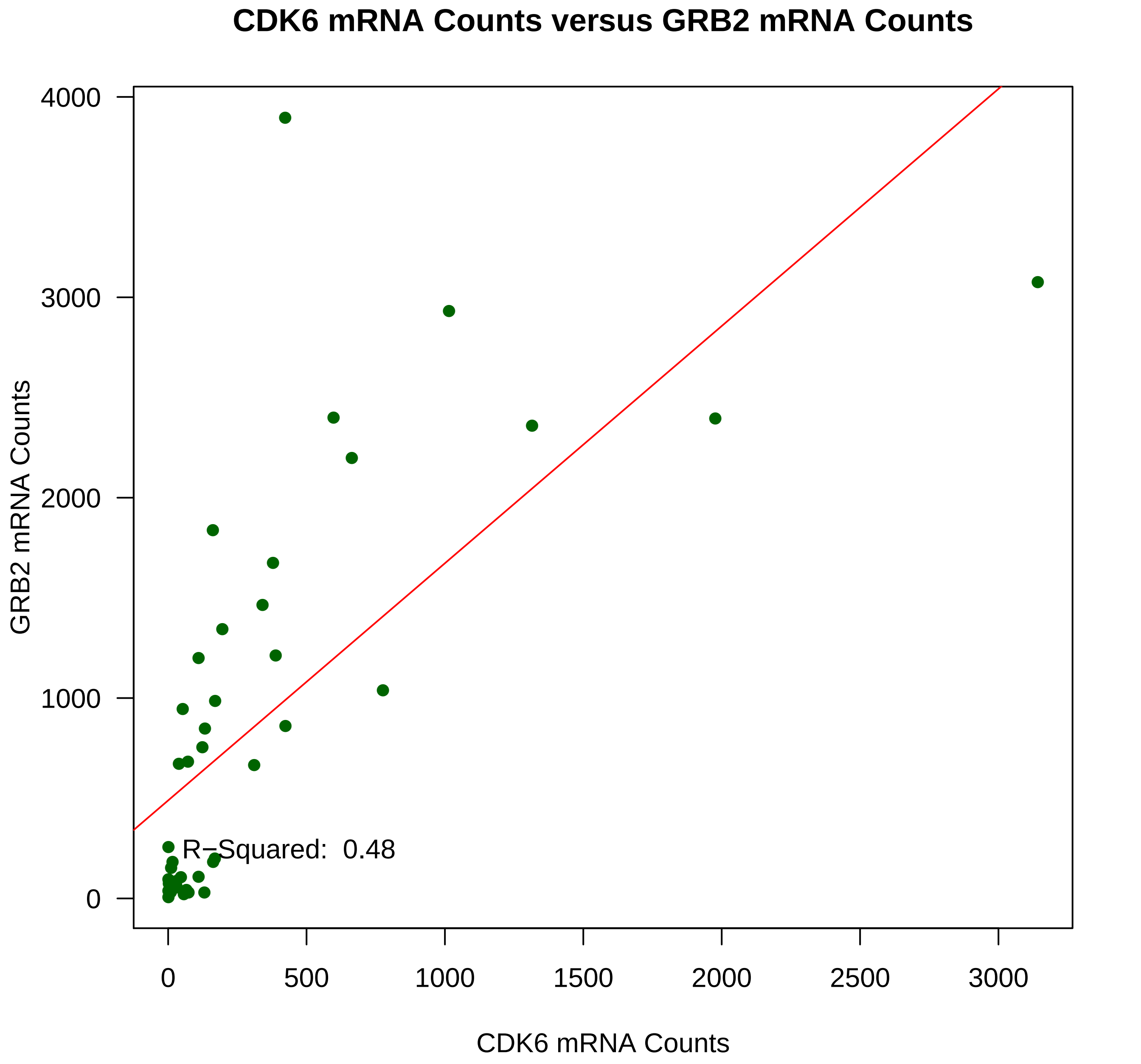

CDK6 mRNA Counts versus GRB2 mRNA Counts
4000
3000
2000
1000
0
GRB2 mRNA Counts
R−Squared: 0.48
0
500
1000
1500
2000
2500
3000
CDK6 mRNA Counts

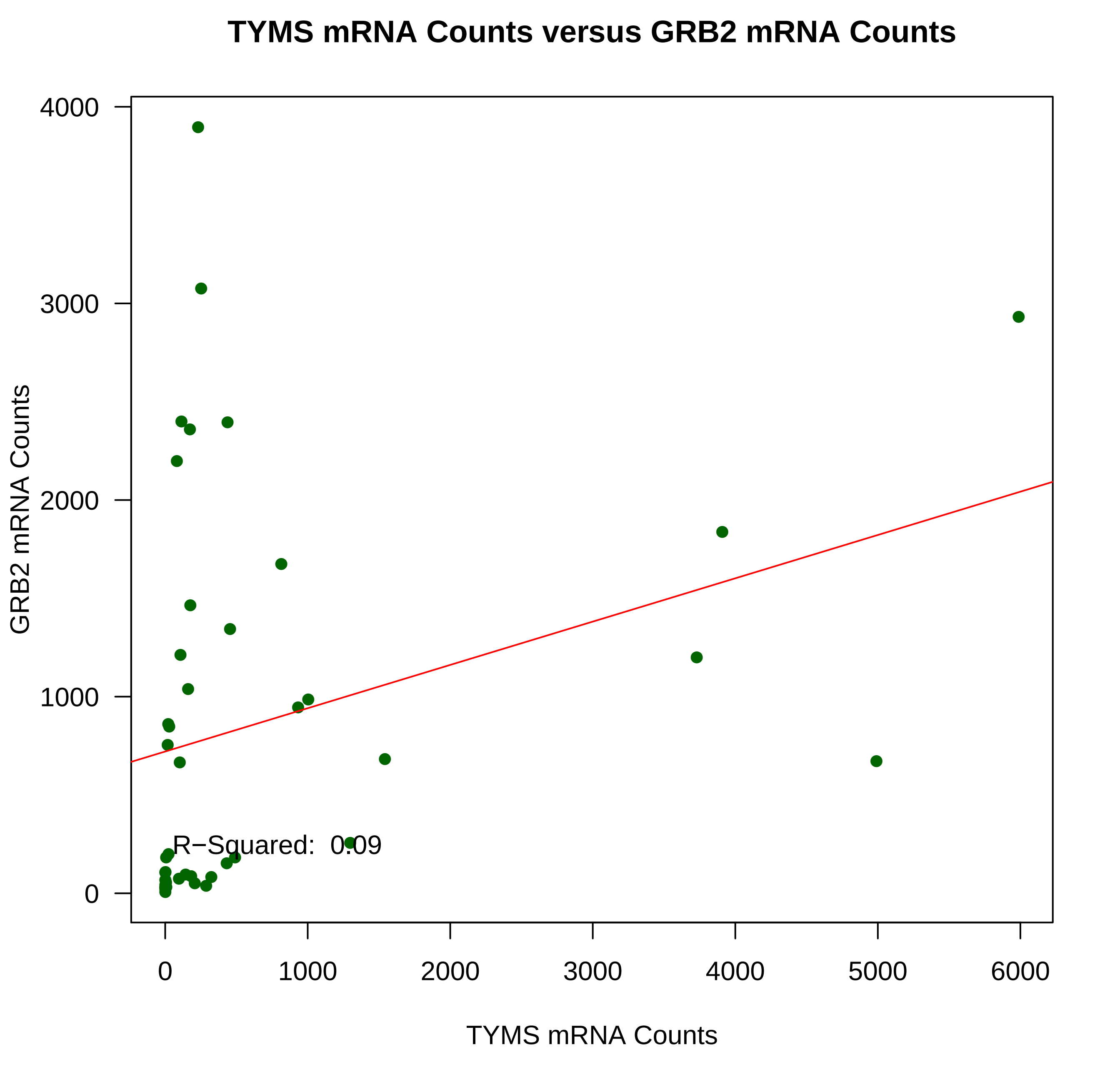
TYMS mRNA Counts versus GRB2 mRNA Counts
GRB2 mRNA Counts
4000
3000
2000
1000
0
R−Squared: 0.09
0
1000
2000
3000
4000
5000
6000
TYMS mRNA Counts

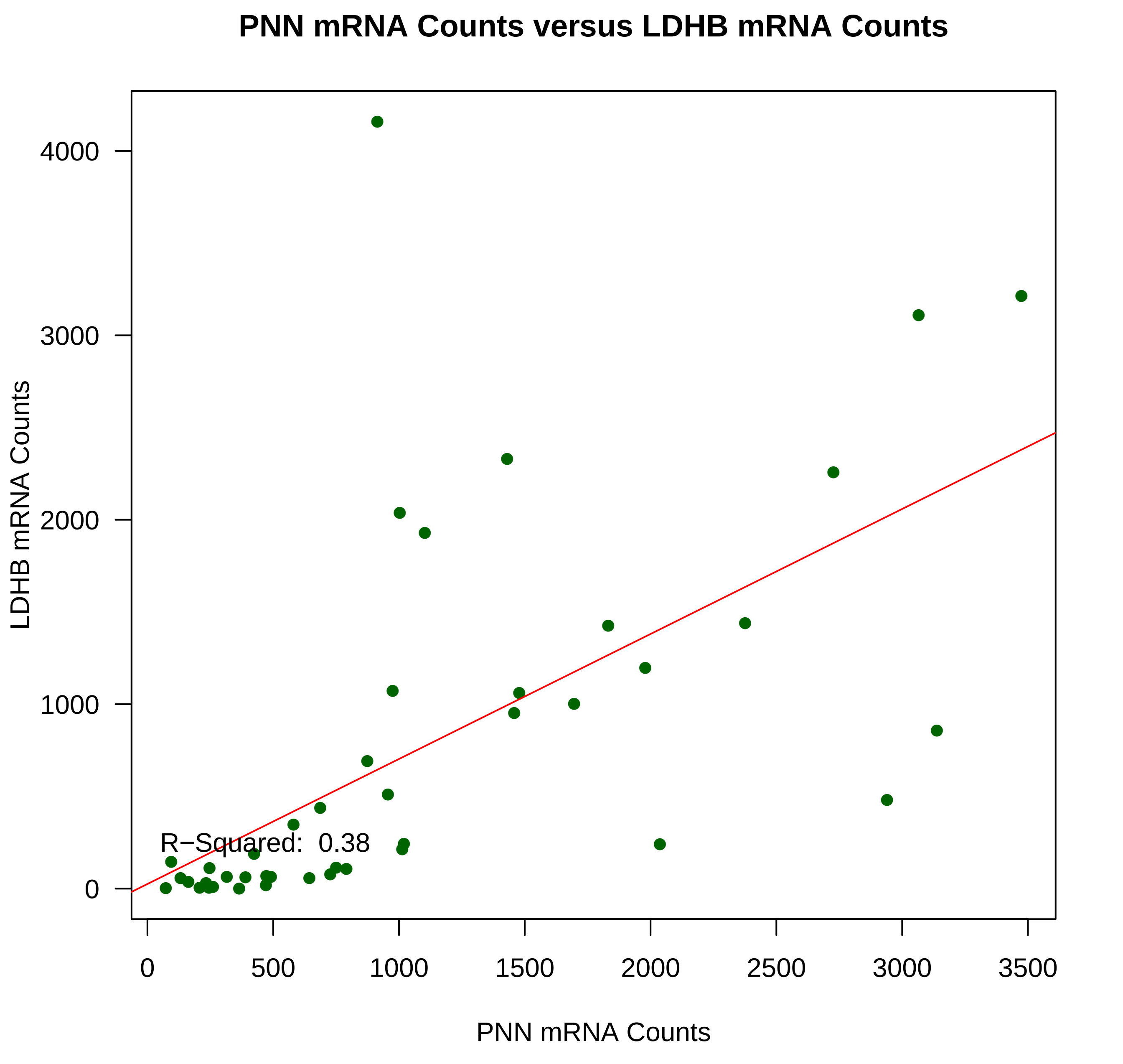

PNN mRNA Counts versus LDHB mRNA Counts
LDHB mRNA Counts
PNN mRNA Counts
R−Squared: 0.38
0
1000
2000
3000
4000
0
500
1000
1500
2000
2500
3000
3500

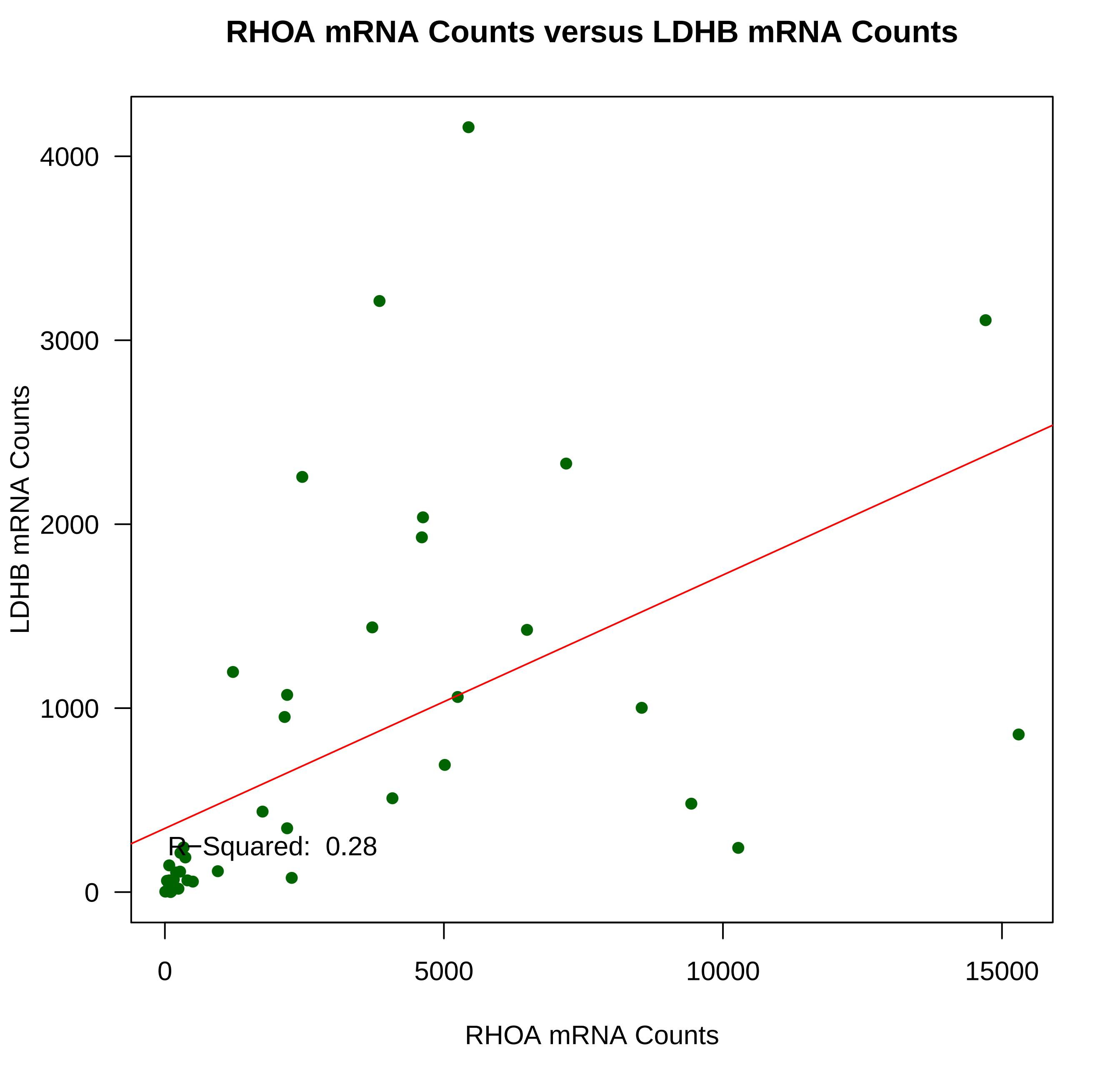

RHOA mRNA Counts versus LDHB mRNA Counts
4000
3000
2000
1000
0
LDHB mRNA Counts
R−Squared: 0.28
0
5000
10000
15000
RHOA mRNA Counts

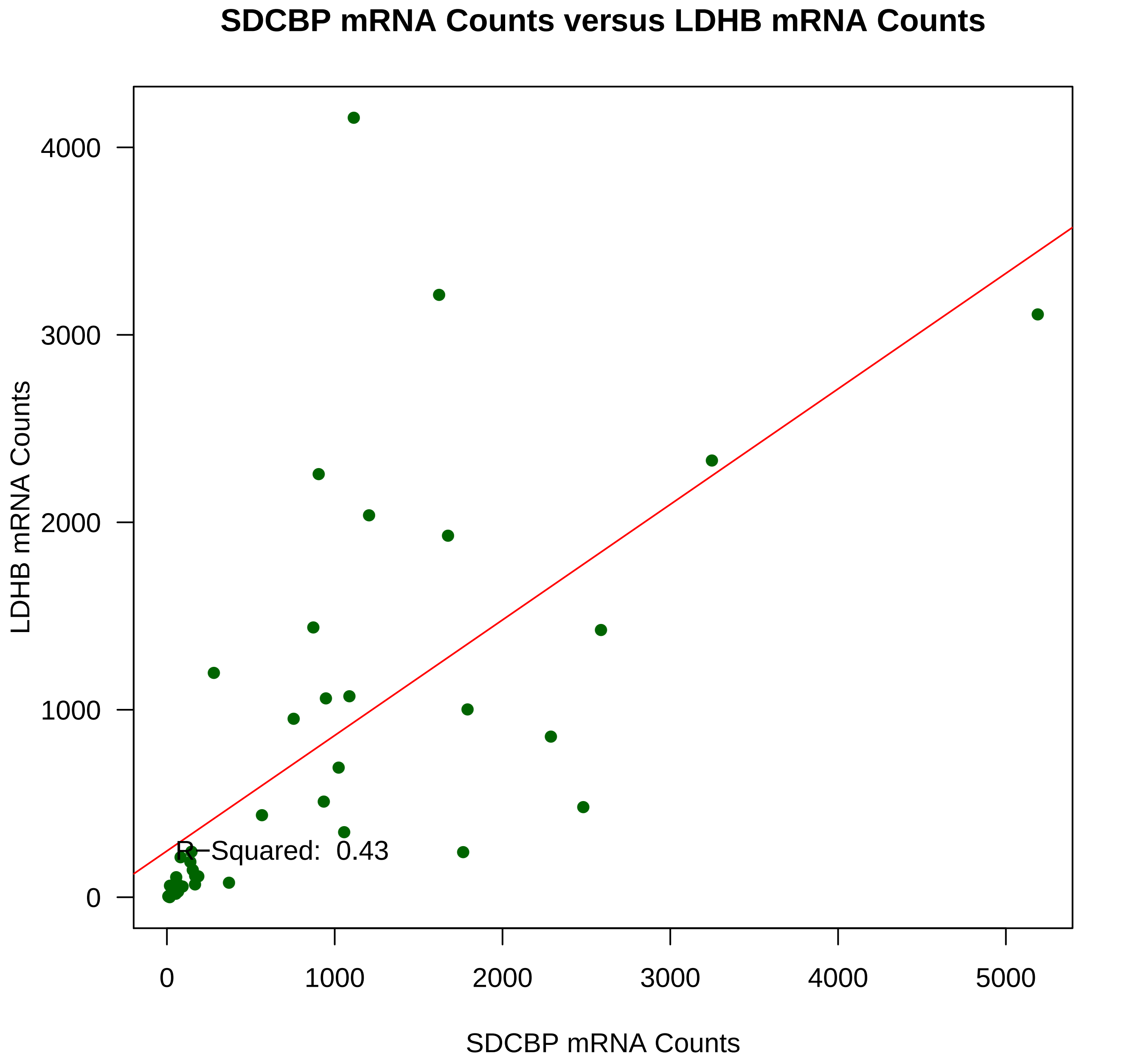

SDCBP mRNA Counts versus LDHB mRNA Counts
LDHB mRNA Counts
0
1000
2000
3000
4000
R−Squared: 0.43
0
1000
2000
3000
4000
5000
SDCBP mRNA Counts

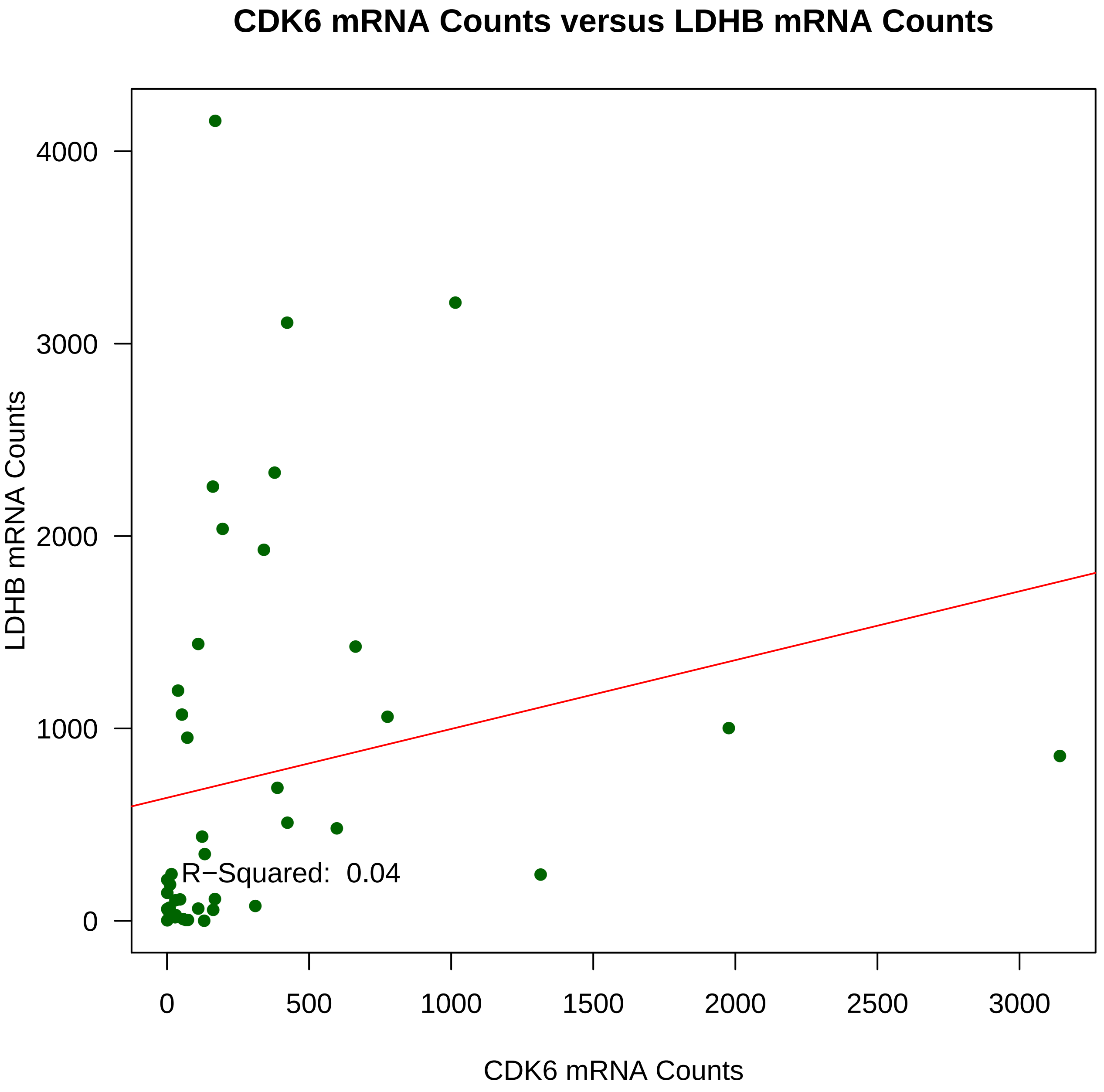
CDK6 mRNA Counts versus LDHB mRNA Counts
LDHB mRNA Counts
4000
3000
2000
1000
0
R−Squared: 0.04
0
500
1000
1500
2000
2500
3000
CDK6 mRNA Counts

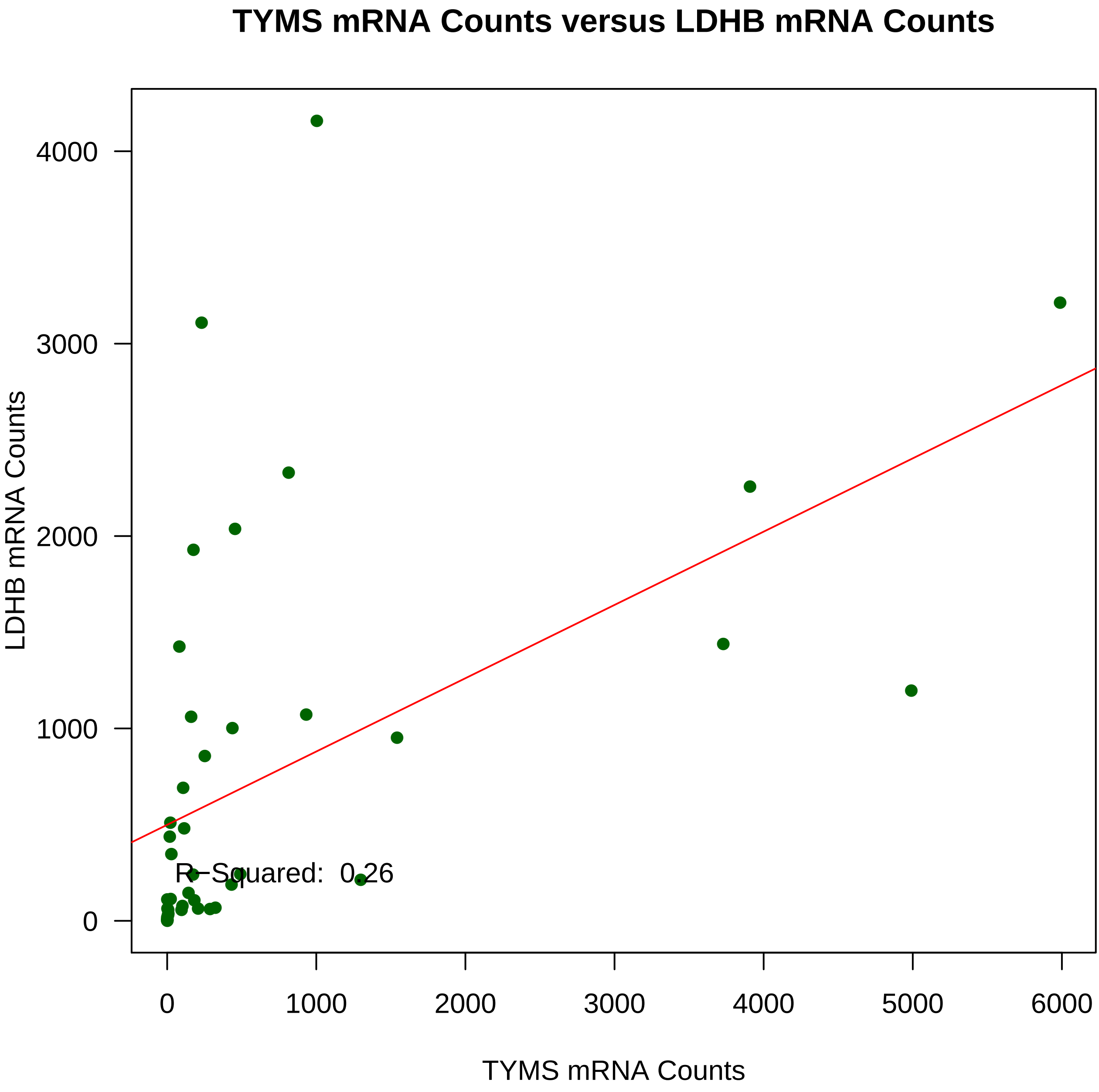
TYMS mRNA Counts versus LDHB mRNA Counts
LDHB mRNA Counts
TYMS mRNA Counts
R−Squared: 0.26
0
1000
2000
3000
4000
5000
6000

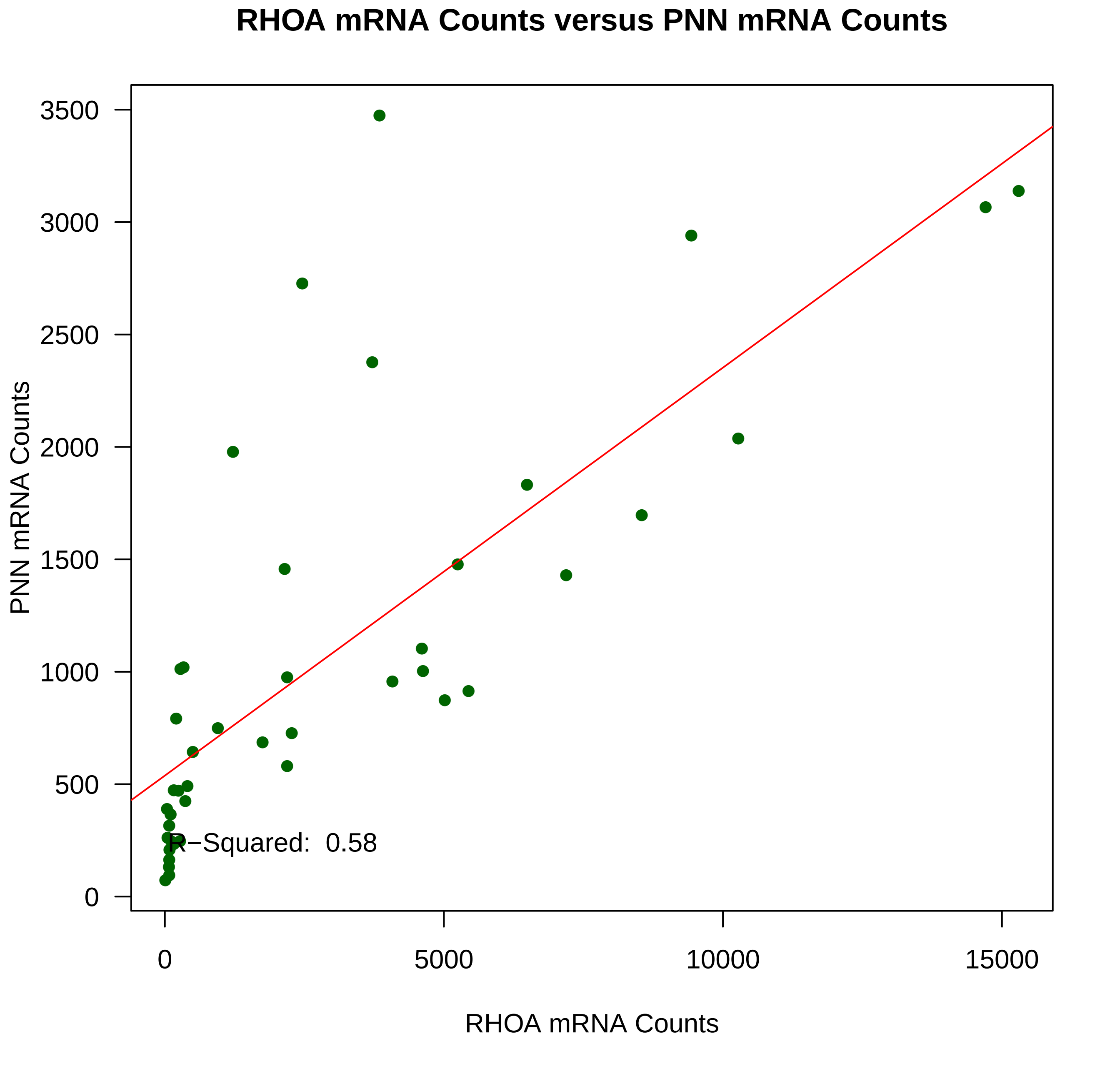

RHOA mRNA Counts versus PNN mRNA Counts
3500
3000
2500
2000
1500
1000
500
0
PNN mRNA Counts
R−Squared: 0.58
0
5000
10000
15000
RHOA mRNA Counts

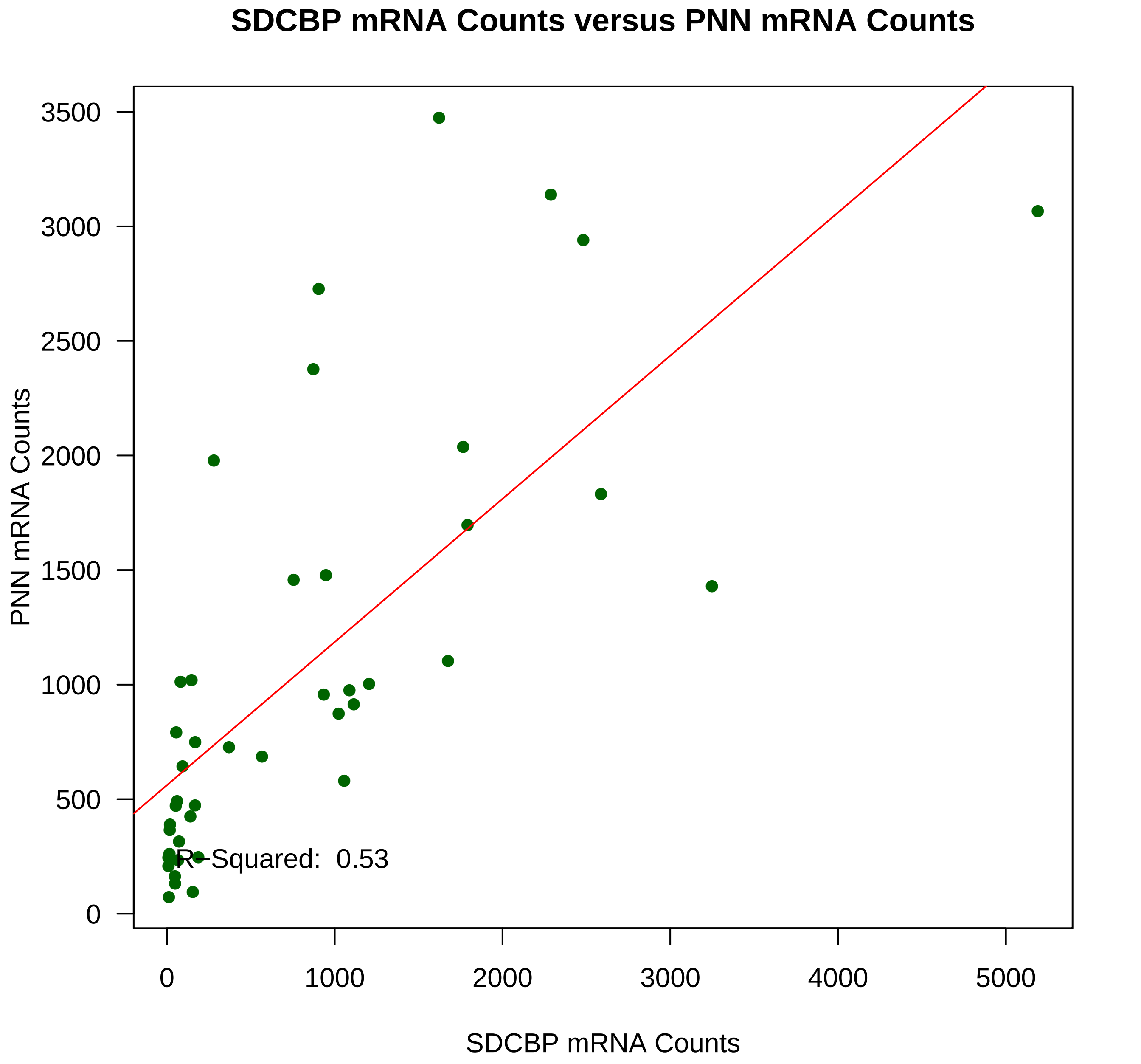
SDCBP mRNA Counts versus PNN mRNA Counts
PNN mRNA Counts
3500
3000
2500
2000
1500
1000
500
0
R-Squared: 0.53
0
1000
2000
3000
4000
5000
SDCBP mRNA Counts

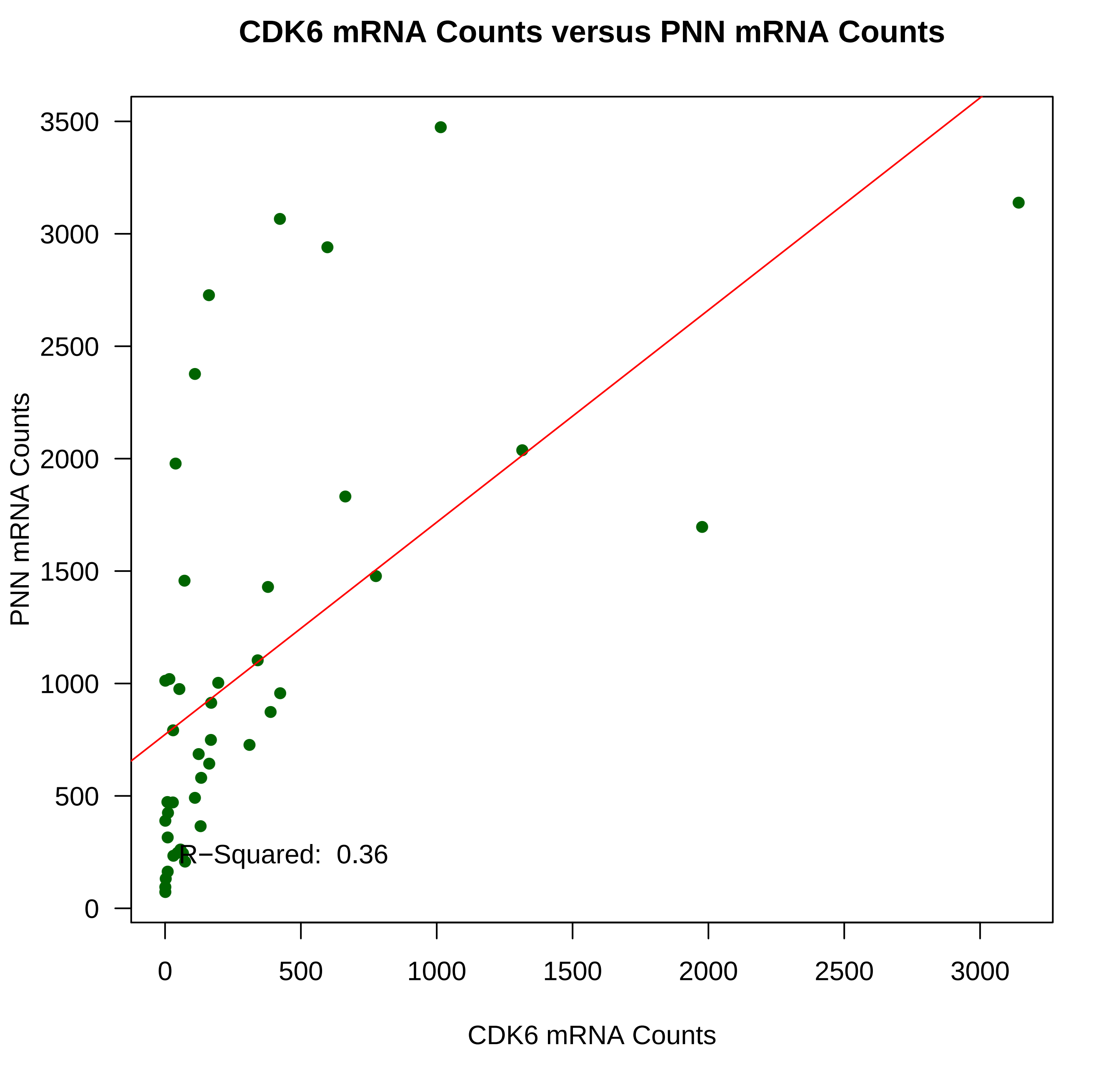
CDK6 mRNA Counts versus PNN mRNA Counts
PNN mRNA Counts
3500
3000
2500
2000
1500
1000
500
0
R−Squared: 0.36
0
500
1000
1500
2000
2500
3000
CDK6 mRNA Counts

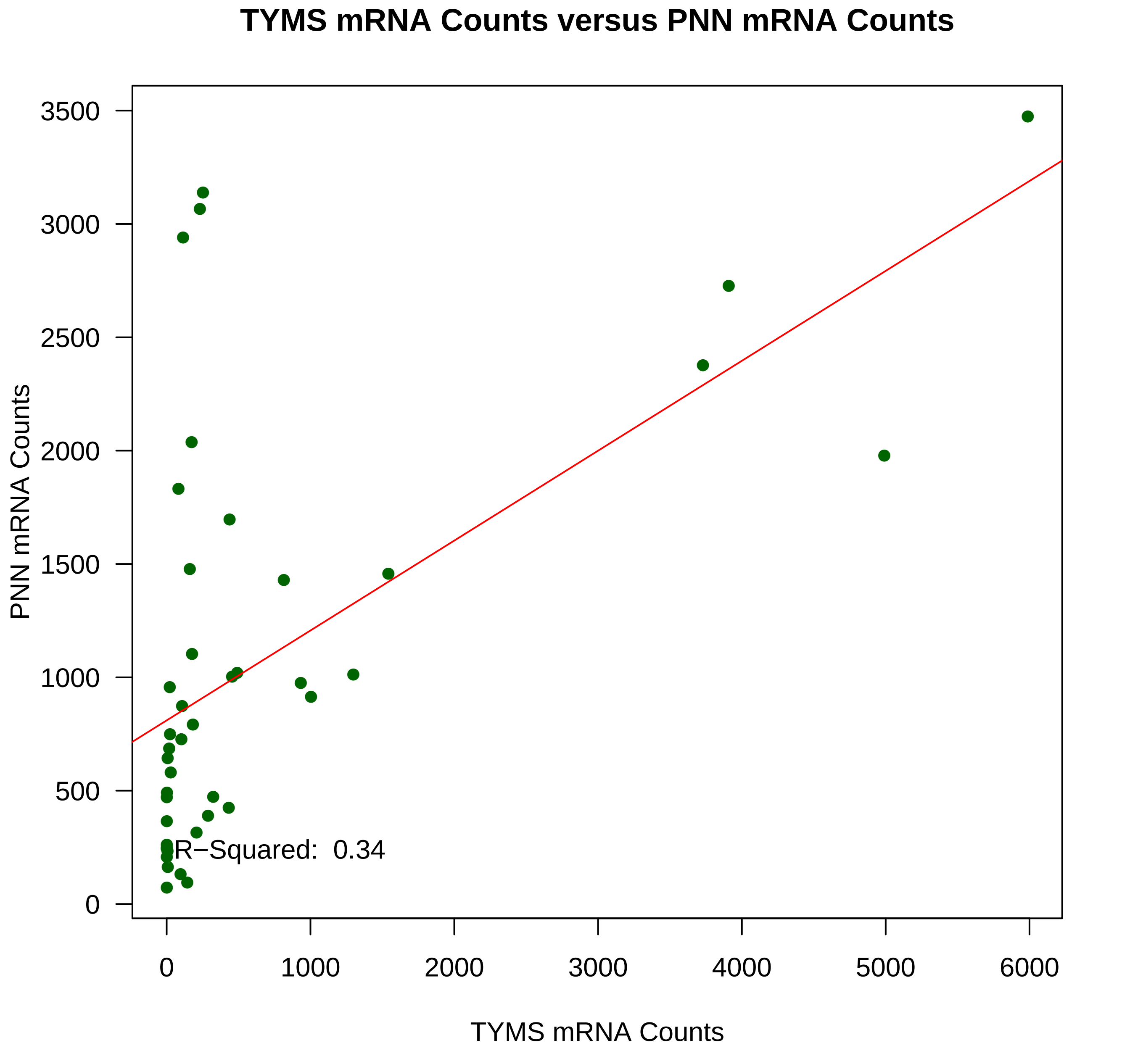

TYMS mRNA Counts versus PNN mRNA Counts
PNN mRNA Counts
3500
3000
2500
2000
1500
1000
500
0
R−Squared: 0.34
0
1000
2000
3000
4000
5000
6000
TYMS mRNA Counts

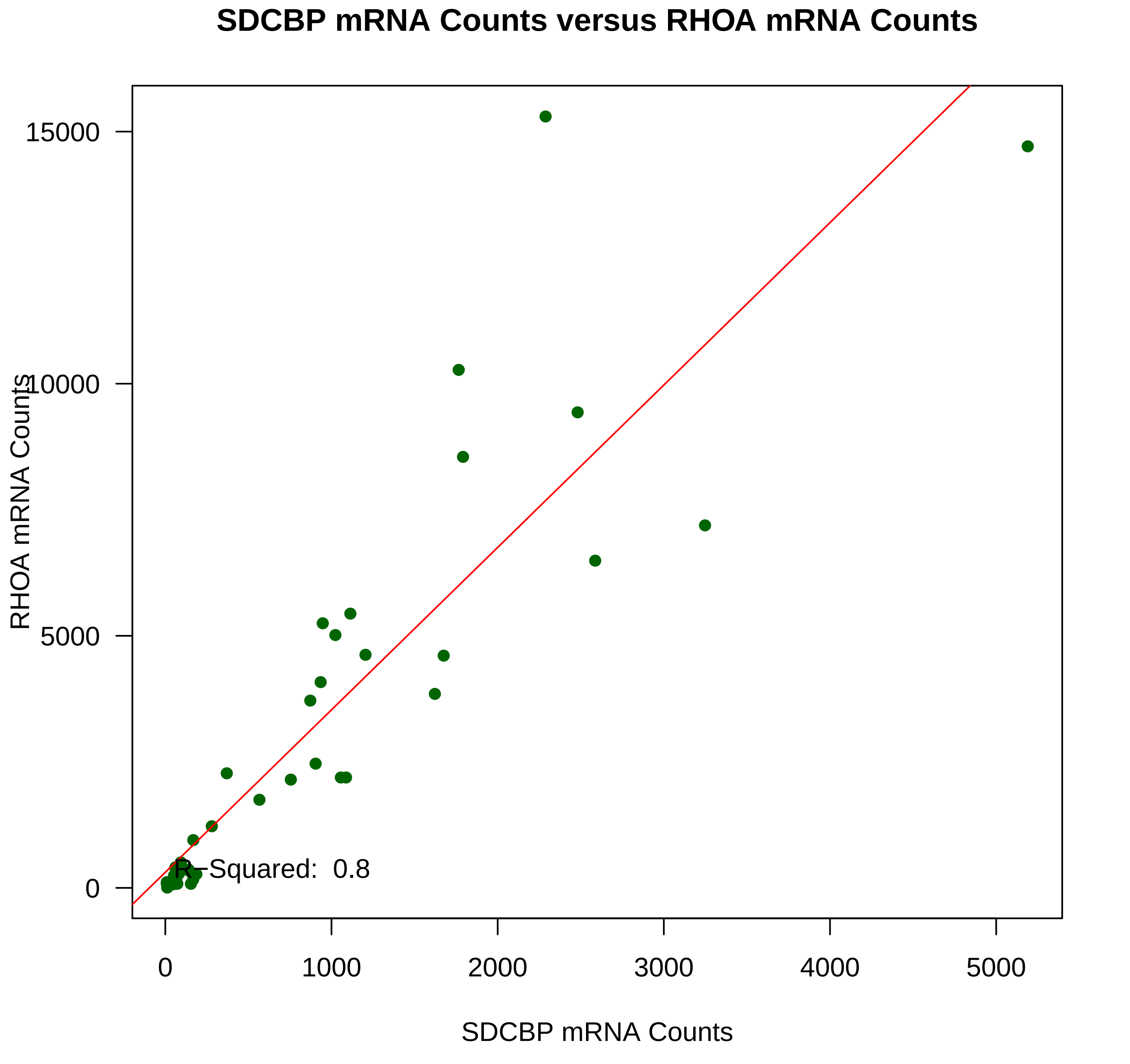

SDCBP mRNA Counts versus RHOA mRNA Counts
RHOA mRNA Counts
15000
10000
5000
0
0
1000
2000
3000
4000
5000
SDCBP mRNA Counts
R−Squared: 0.8

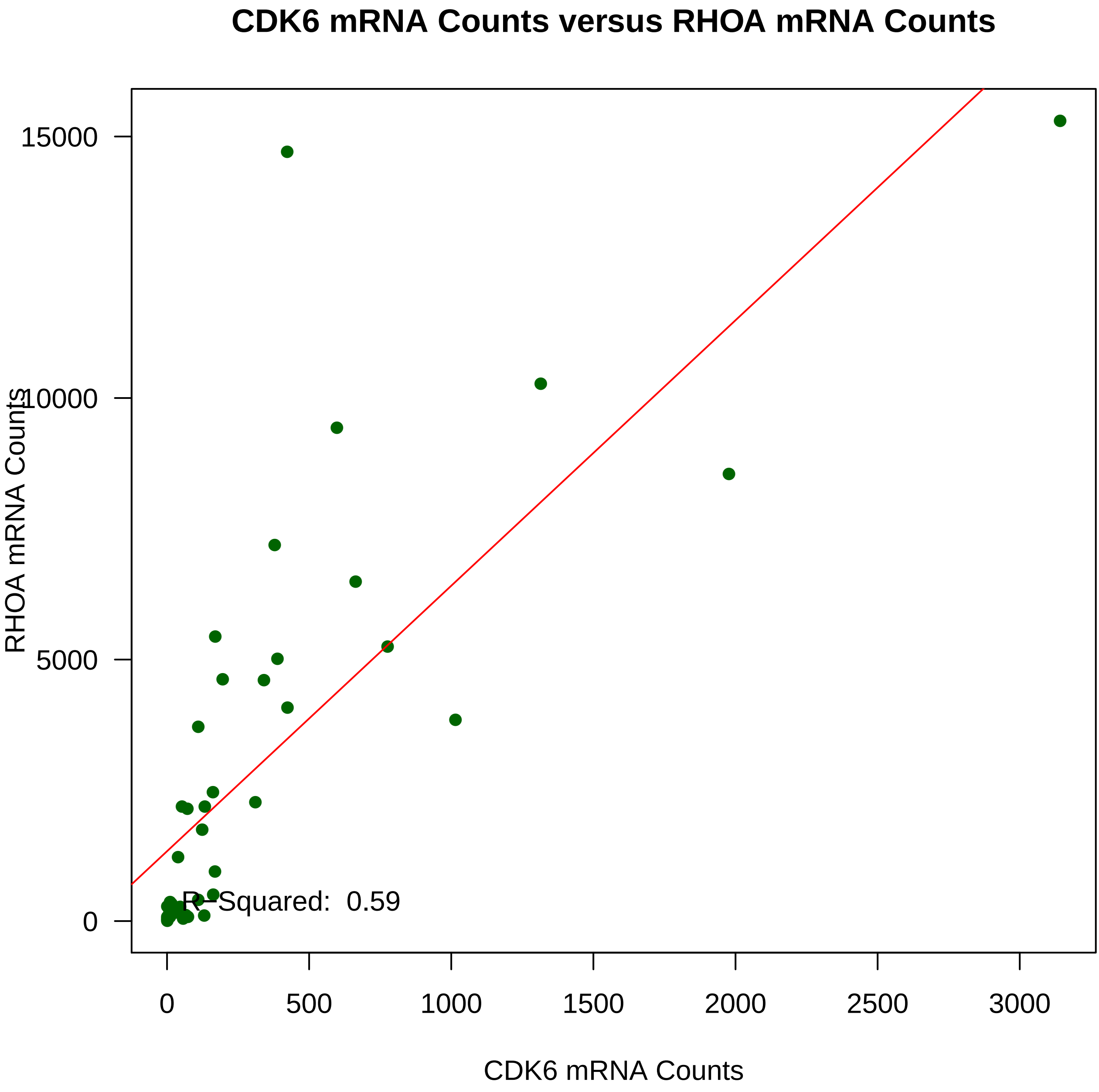
CDK6 mRNA Counts versus RHOA mRNA Counts
RHOA mRNA Counts
15000
10000
5000
0
R−Squared: 0.59
0
500
1000
1500
2000
2500
3000
CDK6 mRNA Counts

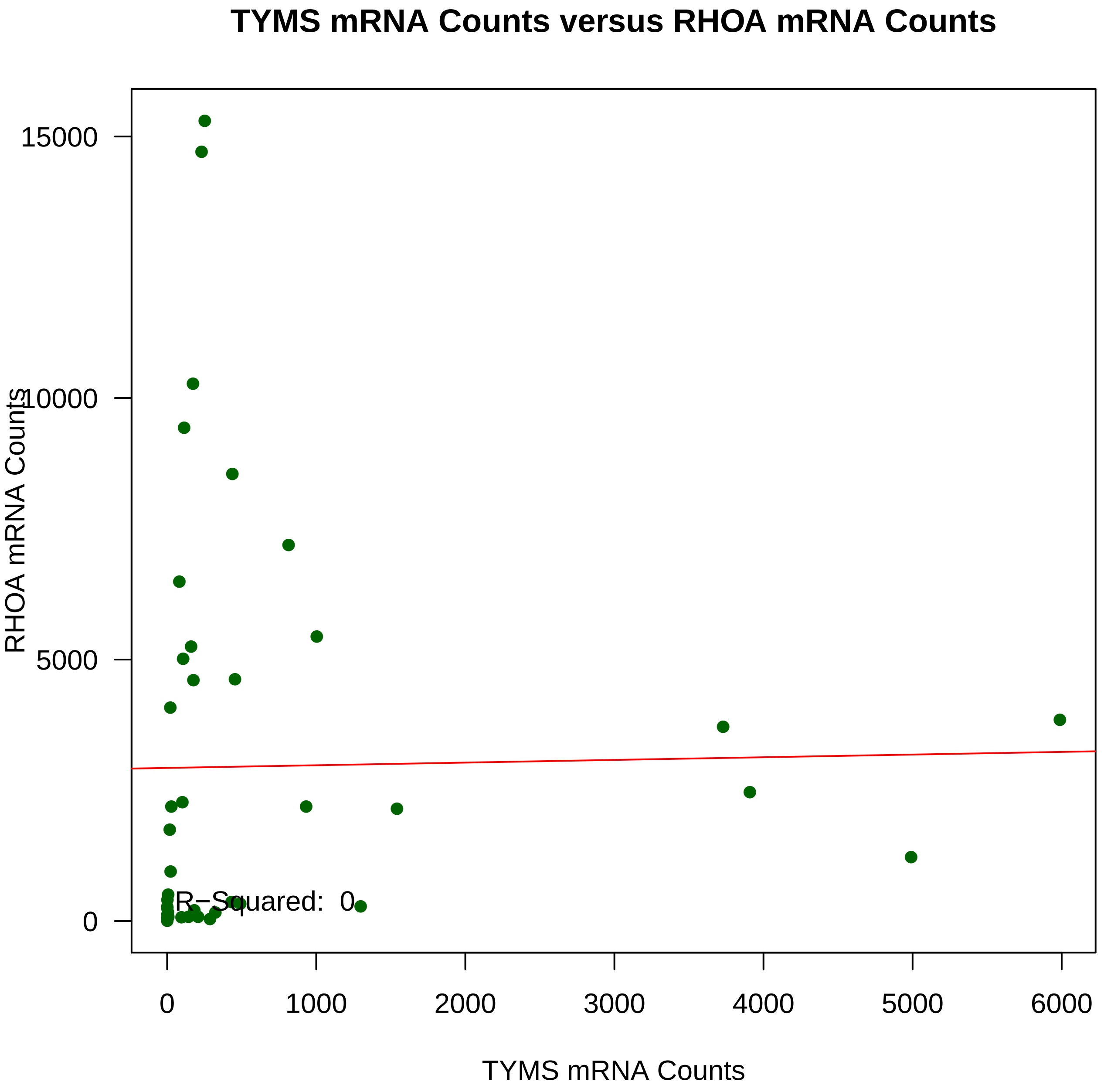

TYMS mRNA Counts versus RHOA mRNA Counts
RHOA mRNA Counts
15000
10000
5000
0
R−Squared: 0
0
1000
2000
3000
4000
5000
6000
TYMS mRNA Counts

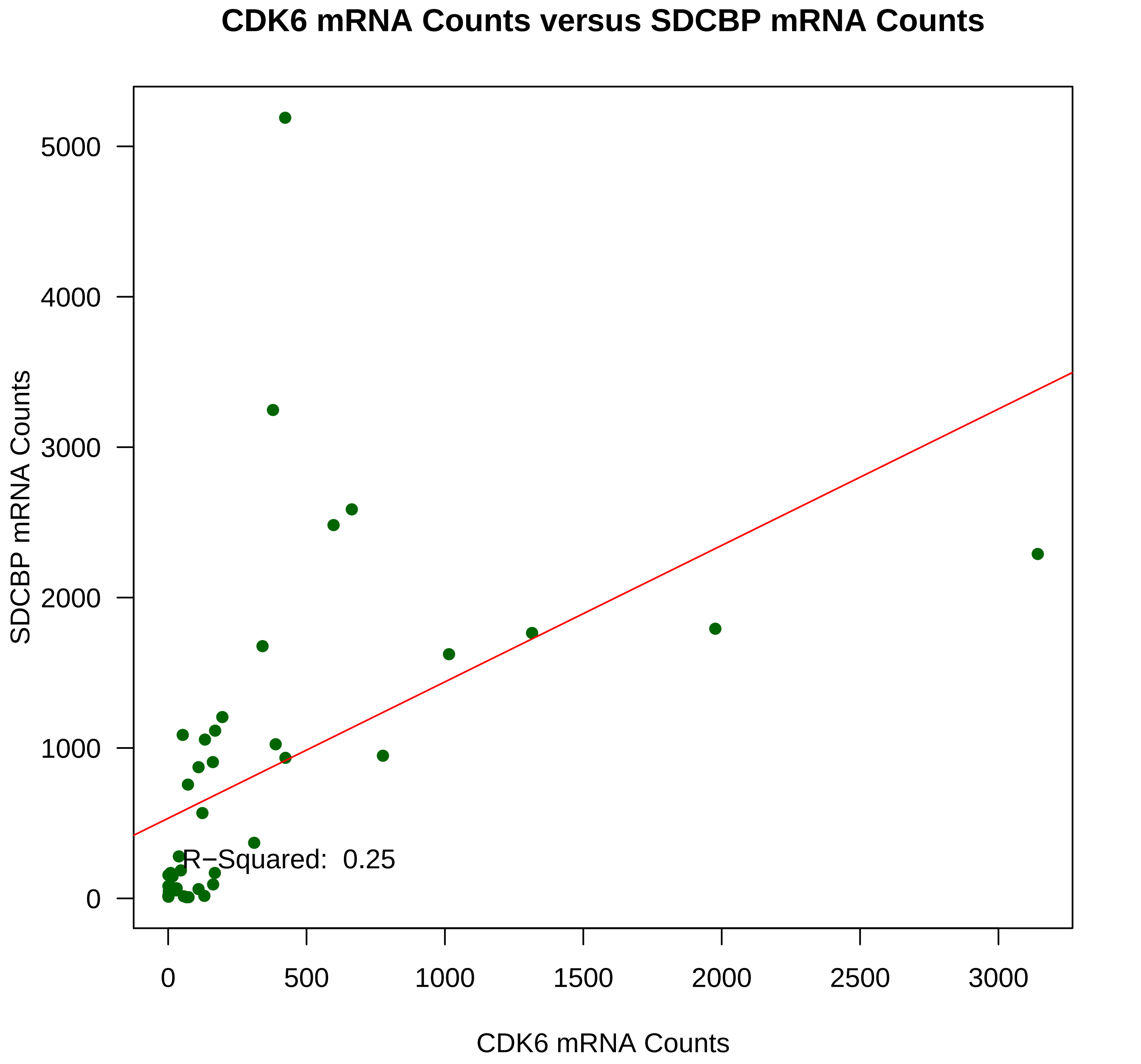

CDK6 mRNA Counts versus SDCBP mRNA Counts
SDCBP mRNA Counts
5000
4000
3000
2000
1000
0
R−Squared: 0.25
0
500
1000
1500
2000
2500
3000
CDK6 mRNA Counts

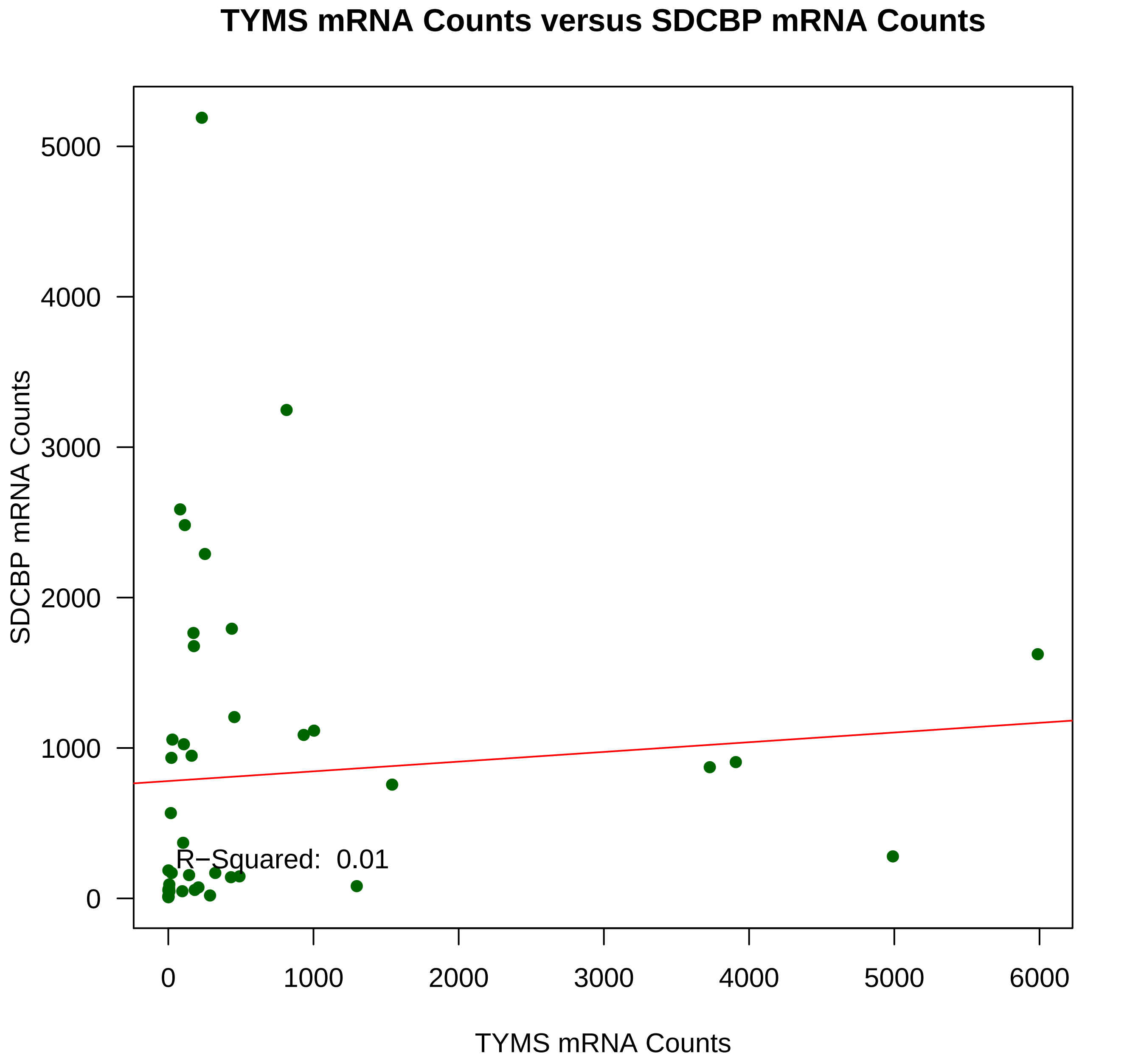
TYMS mRNA Counts versus SDCBP mRNA Counts
SDCBP mRNA Counts
5000
4000
3000
2000
1000
0
R−Squared: 0.01
0
1000
2000
3000
4000
5000
6000
TYMS mRNA Counts

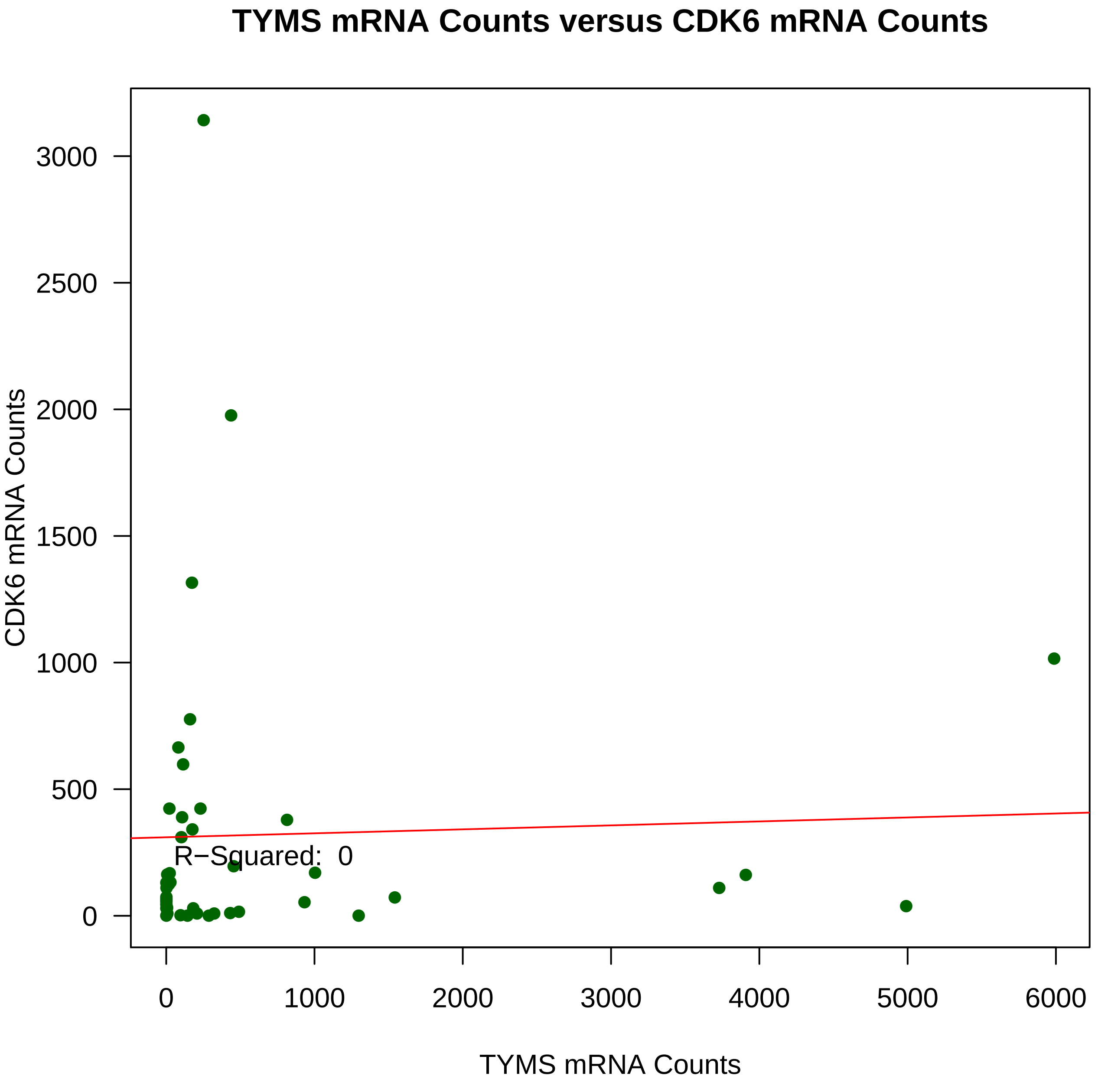

TYMS mRNA Counts versus CDK6 mRNA Counts
3000
2500
2000
1500
1000
500
0
CDK6 mRNA Counts
R−Squared: 0
0
1000
2000
3000
4000
5000
6000
TYMS mRNA Counts
